# CDK4/6 and autophagy inhibitors synergistically induce senescence in Rb positive cytoplasmic cyclin E negative cancers

**DOI:** 10.1038/ncomms15916

**Published:** 2017-06-27

**Authors:** Smruthi Vijayaraghavan, Cansu Karakas, Iman Doostan, Xian Chen, Tuyen Bui, Min Yi, Akshara S. Raghavendra, Yang Zhao, Sami I. Bashour, Nuhad K. Ibrahim, Meghan Karuturi, Jing Wang, Jeffrey D. Winkler, Ravi K. Amaravadi, Kelly K. Hunt, Debu Tripathy, Khandan Keyomarsi

**Affiliations:** 1 Department of Experimental Radiation Oncology, The University of Texas MD Anderson Cancer Center, 6565 MD Anderson Boulevard, Houston, Texas 77030, USA; 2 Graduate School of Biomedical Sciences, The University of Texas MD Anderson Cancer Center, 6767 Bertner Avenue, Houston, Texas 77030, USA; 3 Department of Breast Surgical Oncology, The University of Texas MD Anderson Cancer Center, 1400 Pressler Street, Houston, Texas 77030, USA; 4 Department of Breast Medical Oncology, The University of Texas MD Anderson Cancer Center, 1155 Pressler Street, Houston, Texas 77030, USA; 5 Department of Bioinformatics and Computational Biology, The University of Texas MD Anderson Cancer Center, 1515 Holcombe Boulevard, Houston, Texas 77030, USA; 6 Department of Chemistry, University of Pennsylvania, Philadelphia, Pennsylvania 19104, USA; 7 Division of Hematology Oncology, Perelman School of Medicine, University of Pennsylvania, Philadelphia, Pennsylvania 19104, USA

## Abstract

Deregulation of the cell cycle machinery is a hallmark of cancer. While CDK4/6 inhibitors are FDA approved (palbociclib) for treating advanced estrogen receptor-positive breast cancer, two major clinical challenges remain: (i) adverse events leading to therapy discontinuation and (ii) lack of reliable biomarkers. Here we report that breast cancer cells activate autophagy in response to palbociclib, and that the combination of autophagy and CDK4/6 inhibitors induces irreversible growth inhibition and senescence *in vitro,* and diminishes growth of cell line and patient-derived xenograft tumours *in vivo*. Furthermore, intact G1/S transition (Rb-positive and low-molecular-weight isoform of cyclin E (cytoplasmic)-negative) is a reliable prognostic biomarker in ER positive breast cancer patients, and predictive of preclinical sensitivity to this drug combination. Inhibition of CDK4/6 and autophagy is also synergistic in other solid cancers with an intact G1/S checkpoint, providing a novel and promising biomarker-driven combination therapeutic strategy to treat breast and other solid tumours.

## Abstract (web summary)

CDK4/6-Cyclin D pathway is often deregulated in cancer; therefore specific inhibitors have been developed. Here the authors show that treatment with CDK4/6 inhibitors activate autophagy in breast cancer cells; thus, combination of such inhibitors with autophagy inhibitors results in a synergistic effect on tumour growth.

Deregulation of the cell cycle checkpoint proteins, such as cyclin-dependent kinases CDK4 and CDK6, is a key hallmark of cancer, resulting in uncontrolled cellular proliferation and tumorigenesis. Selective CDK4/6 inhibitors, palbociclib, ribociclib and abemaciclib, have shown promising preclinical and clinical activities in numerous solid tumours^1^. In particular, palbociclib (PD-0332991 or Ibrance) has shown clear benefits in Phase II (PALOMA-1) and III (PALOMA-2 and -3) clinical trials in advanced estrogen receptor-positive (ER+) breast cancers, doubling the progression-free survival (PFS) compared to letrozole or fulvestrant alone^2^,^3^,^4^. Palbociclib was recently approved by the U.S. Food and Drug Administration for both these indications and is currently being evaluated clinically in other solid tumours^5^. Moreover, numerous Phase III clinical trials are currently underway with ribociclib (LEE011) and abemaciclib (LY2835219) in breast and other solid tumours^5^.

Despite these promising clinical advances with CDK4/6 inhibitors, there are three major limitations for this treatment: (i) adverse events (Grade 3&4 neutropenia (56%) and leukopenia (25.2%)), which lead to interruption and/or discontinuation of treatment, possibly attenuating therapeutic benefit^2^, (ii) ∼16% (ref. 3) of the ER+ cancer patients do not respond to palbociclib or exhibit progression within 24 weeks, and half the patients develop clinical resistance with progression within 25 months, resulting in no overall survival benefit and (iii) lack of reliable biomarkers that can be used as prognostic indicators for advanced ER+ breast cancer. Clinical investigation of prognostic biomarkers to-date, have failed to show a correlation to PFS in patients treated with palbociclib^2^,^6^.

Mechanistically, palbociclib induces G1 arrest and cellular senescence *in vitro* and inhibits growth of tumour xenografts *in vivo*^7^,^8^. Palbociclib also induces autophagy in fibroblasts and leukemic cells^9^,^10^. Autophagy is a stress tolerance mechanism in cancer that recycles cellular constituents by engulfing them into a double-membrane vesicle called autophagosome, which eventually fuses with lysosomes to facilitate degradation of the cellular constituents and generation of energy for survival^11^. Autophagy mediates resistance to numerous cancer-targeting agents^12^,^13^, making it a promising co-target and leading to the recent development of numerous clinical trials with autophagy inhibitors, chloroquine (CQ) and hydroxychloroquine (HCQ)^14^.

In this study we address the aforementioned limitations of palbociclib therapy by interrogating the mechanisms by which the drug’s action can be enhanced in breast cancer. Our studies suggest that the selectivity and efficacy of palbociclib is improved through a biomarker-driven combination treatment approach that targets CDK4/6 and autophagy in breast and other solid tumours.

## Results

### CDK4/6 inhibition induces ROS-mediated senescence and autophagy

**Figure 1:**
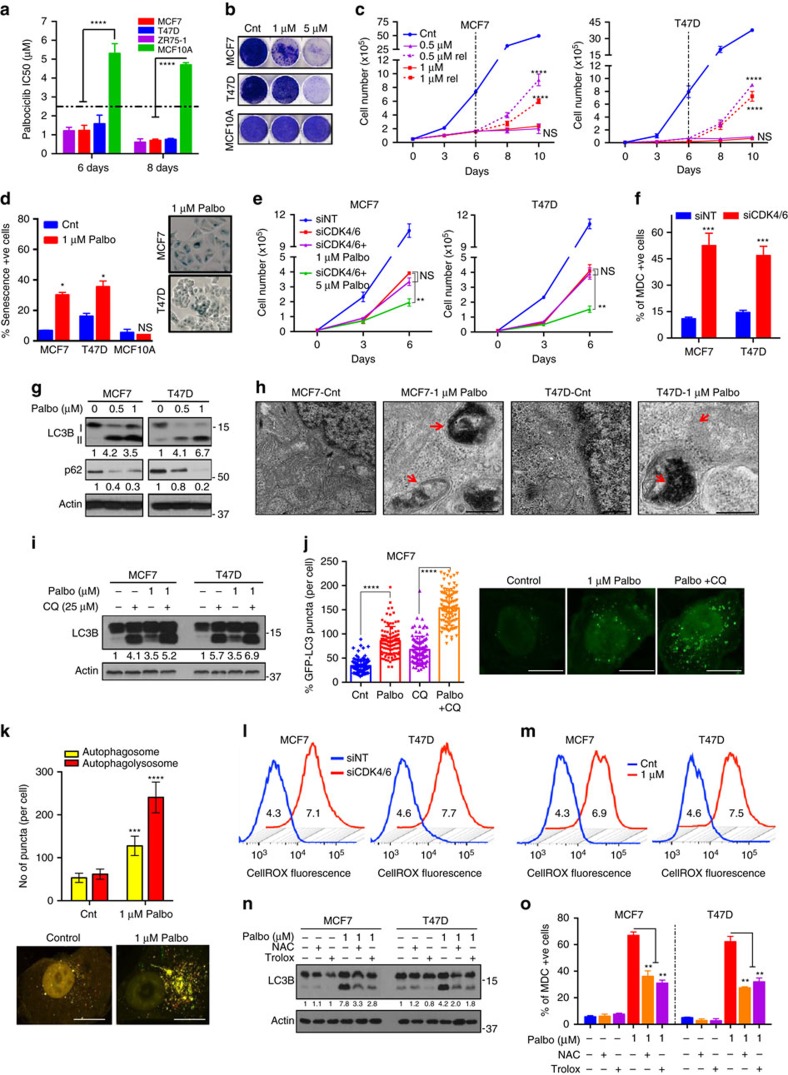
CDK4/6 inhibition induces ROS-mediated senescence and autophagy. (**a**) IC_50_ values of palbociclib in ER+ (MCF7, T47D, ZR75-1) and HMEC (MCF10A) cells treated with increasing concentrations of palbociclib (0.01–12 μM). MCF7, T47D and MCF10A cells were treated with DMSO (Cnt) or palbociclib for 6 days, allowed to recover (rel) and subjected to (**b**) Clonogenic assay, (**c**) Cell counting and (**d**) SA-ß galactosidase activity measurement with representative images. (**e**) Impact of combined siRNA knockdown of CDK4 and CDK6 along with treatment with DMSO, 1 or 5 μM palbociclib for 6 days. (**f**) Measurement of MDC-positive acidic vesicles, including autophagosomes, by flow cytometry in MCF7 and T47D cells treated siRNA against CDK4/6. (**g**) Western blot for LC3B I, II and p62 upon treatment with palbociclib for 6 days. (**h**) Representative TEM microphotograph of cells treated with DMSO (Cnt) or 1 μM palbociclib for 6 days. Red arrows indicate double-membraned autophagosomes. Scale bars, 500 nm. (**i**) Western blot of LC3B and p62 in MCF7 and T47D cells treated with a combination of 25 μM CQ for 1 h and palbociclib for 6 days. (**j**,**k**) Quantification of GFP-LC3 puncta (**j**) and RFP-GFP-LC3 puncta (**k**) and representative images in MCF7 cells treated with 1 μM palbociclib and/or 25 μM CQ for 48 h. (**l**,**m**) Cellular ROS measurement and quantification (MFI) of ROS levels in MCF7 and T47D cells upon transfection with siRNA against CDK4 and CDK6 (**l**) or treatment with palbociclib for 6 days (**m**). (**n**,**o**) LC3B and p62 protein levels (**n**) and MDC +ve cells (**o**) upon combined treatment with 10 mM NAC or 0.1 mM Trolox and 5 μM palbociclib for 6 days. All data represent mean±s.d. from three independent experiments; *P* values were calculated in comparison with cells treated with DMSO (Control) unless indicated. NS: *P*>0.05; *P<0.05; **P<0.01; ***P<0.001; ****P<0.0001.

Analysis of The Cancer Genome Atlas's (TCGA) ER+ breast tumour cohort revealed alterations in the CDK4/CDK6/cyclin D pathway in about 35% of the patients, making them an ideal population for targeting CDK4 and CDK6 (Supplementary Fig. 1a). To interrogate the biological effect of downregulating these proteins, we knocked down CDK4 and/or CDK6 in two ER+ breast cancer cell lines, MCF7 and T47D via shRNA and siRNA (Supplementary Fig. 1b,c and f). Results revealed significant growth inhibition only with combined knockdown of CDK4 and CDK6 (Supplementary Fig. 1d,e and g). Next, we examined the growth-inhibitory potential of the CDK4/6 inhibitor palbociclib in ER+ cell lines (MCF7, T47D, ZR75-1) and in an immortalized non-transformed human mammary epithelial cell line (MCF10A). While palbociclib inhibited growth of ER+ cells in a time- and dose-dependent manner, MCF10A was more resistant to this drug (Fig. 1a,b and Supplementary Fig. 2a–c). To examine their ability to recover from palbociclib-mediated growth inhibition, cells were treated for 6 days and cultured in the absence of drug for 4 days (Supplementary Fig. 2d). Treatment with palbociclib at doses of 1 μM or less resulted in reversible G1 arrest and growth inhibition, while only higher doses (>2.5 μM) resulted in irreversible inhibition of growth and cell cycle progression in the ER+ cells but not in MCF10A (Fig. 1c and Supplementary Fig. 2e–h). Palbociclib did not trigger apoptosis (Supplementary Fig. 3a–d), but significantly increased senescence-associated ß-galactosidase (SA-ß gal) activity and cellular complexity in a dose-dependent manner, both characteristics of cells undergoing senescence (Fig. 1d and Supplementary Fig. 3e,f). Further, western blot analysis showed a dose-dependent decrease in known palbociclib effectors, pRb, FOXM1 (ref. 8) and total Rb levels, a consequence of G1 arrest, as previously reported in breast cancer^15^,^16^ (Supplementary Fig. 3g). To confirm the specificity of palbociclib-mediated growth inhibition and examine potential off-target effects, MCF7 and T47D cells with shRNA- or siRNA-mediated CDK4 and/or CDK6 knockdown were treated with palbociclib. Combined knockdown of CDK4 and CDK6 was able to recapitulate the drug effect at 1 μM but not 5 μM, indicating that the irreversible grown inhibition and high levels of senescence observed at 5 μM could be due to off-target effects at higher palbociclib concentrations (Fig. 1e and Supplementary Fig. 3h).

A stress response signal mediated by cell cycle arrest is autophagy, a catabolic process that can degrade ROS and mediate reversal of G1 arrest and senescence^12^,^14^,^17^. Hence, it was hypothesized that CDK4/6 inhibition-mediated cell cycle arrest (on-target effect at 1 μM palbociclib) triggers autophagy as a stress response, which prevents the induction of senescence at these doses. To test this hypothesis, we assessed autophagy by: (i) monodansylcadavarine (MDC) staining^18^, (ii) transmission electron microscopy (TEM)^19^, (iii) GFP-LC3 puncta, (iv) western blot analysis of LC3B-II (autophagosomal surface protein^20^) and p62 (SQSTM1, an autophagic substrate^21^) and (v) reverse-phase protein array (RPPA) analysis of key autophagy proteins. siRNA against CDK4/6 or palbociclib treatment (low doses) of MCF7 and T47D cells significantly increased levels of MDC, LC3B-II and other key autophagy proteins (Atg-7, Beclin-1, BNIP3), while decreasing BCl2 (a known inhibitor of autophagy^22^) and p62 at low doses (Fig. 1f,g and Supplementary Fig. 4a–d). Further, TEM and GFP-LC3 assays showed significant accumulation of double-membrane electron-dense autophagosomes and GFP-LC3 puncta, respectively, in Palbociclib (1 μM)-treated cells (Fig. 1h,j and Supplementary Figs 4e, 5d). We next examined the presence of an intact autophagic flux following palbociclib treatment by: (i) flux ratio (LC3B-II to LC3B-I and LC3B-II to p62), (ii) treatment with lysosomal block CQ^23^, (iii) GFP-LC3 puncta with CQ treatment and (iv) RFP-GFP-LC3 dual-reporter assay^24^. Treatment with low-dose (1 μM) palbociclib, exhibited higher flux ratios (Supplementary Fig. 5a), elevated MDC and LC3B-II levels with CQ treatment (Fig. 1i and Supplementary Fig. 5b,c), significantly higher GFP-LC3 puncta with CQ treatment (Fig. 1j and Supplementary Fig. 5d) and significant increase in RFP+GFP+ puncta (autophagosomes) and RFP+ puncta (autophagolysosomes) (Fig. 1k and Supplementary Fig. 5e), all demonstrative of an intact autophagic flux.

Another stress response signal associated with cell cycle arrest, autophagy and senescence is oxidative stress induced by reactive oxygen species (ROS), which also mediates senescence^25^,^26^. This led us to hypothesize that CDK4/6 inhibition mediated by on-target effects of palbociclib (1 μM) may induce ROS, which in turn triggers autophagy. Consistent with this, siRNA knockdown of CDK4/6 or palbociclib treatment increased cellular ROS levels in a dose-dependent manner in ER+ cells but not in MCF10A as measured by CellROX assay and RPPA (Fig. 1l,m and Supplementary Fig. 6a–c). To examine the dependence of autophagy on ROS, we ablated ROS in palbociclib-treated cells by treating with N-acetyl-L-cysteine (NAC) or trolox^27^,^28^, and observed significant decreases in MDC and LC3B-II expression (Fig. 1n,o and Supplementary Fig. 6d), suggesting that palbociclib-induced autophagy is dependent on ROS production.

Collectively, these results demonstrate that palbociclib induces ROS, leading to intact autophagy and reversible G1 arrest and growth inhibition at low concentrations (≤1 μM; on-target effect) (Supplementary Fig 6e).

### Autophagy inhibition sensitizes breast cancer to CDK4/6 inhibition

**Figure 2:**
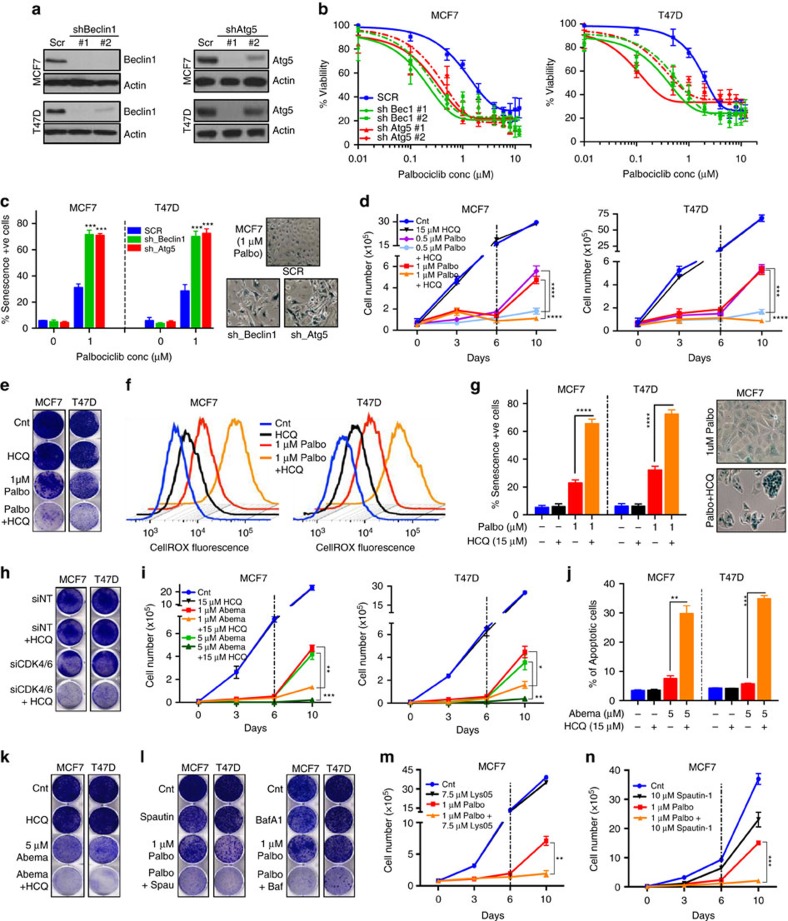
Autophagy inhibition sensitizes cells to CDK4/6 inhibition. (**a**) Western blot for Beclin-1 and Atg-5 in MCF7 and T47D cells after transfection with Scrambled (Scr), Beclin-1 or Atg5 shRNA. MCF7 and T47D cells with Beclin-1 or Atg5 knocked down were treated with palbociclib for 6 days and subjected to (**b**) dose–response assay after 6 days of recovery and (**c**) SA-ß galactosidase measurement. *P* values calculated in comparison with SCR −1 μM palbociclib. MCF7 and T47D cells were treated with combination of palbociclib and 15 μM HCQ for 6 days. Cells were allowed to recover for 4 or 6 days in drug-free media and subjected to (**d**) Cell counting, (**e**) clonogenic assay, (**f**) cellular ROS measurement and (**g**) SA-ß galactosidase measurement. (**h**) Clonogenic assay to measure impact of combined siRNA against CDK4/6 and treatment with 15 μM HCQ for 6 days. MCF7 and T47D cells were treated with 5 μM abemaciclib combined with 15 μM HCQ for 6 days and recovery for 4 or 6 days and subjected to (**i**) cell counting (**j**) measurement of total apoptotic cells (early apoptosis: Annexin V+/propidium iodide− and late apoptosis: Annexin V+/propidium iodide+) and (**k**) clonogenic assay. (**l**) Clonogenic assay in MCF7 and T47D cells treated with 10 μM spautin-1 or 1 nM bafilomycin A1 (Baf-A1) combined with 1 μM palbociclib for 6 days and recovery for 6 days. (**m**,**n**) Cell counting to assess growth of cells treated with 1 μM palbociclib and 7.5 μM Lys-05 (**m**) or 10 μM Spautin-1 (**n**) for 6 days and recovery for 4 days. All data represent mean±s.d. from three independent experiments; NS: *P*>0.05; *P<0.05; **P<0.01; ***P<0.001; ****P<0.0001.

To test our hypothesis that it is autophagy that protects ER+ breast cancer cells from palbociclib-induced senescence at low doses, we first downregulated two crucial autophagy genes, Beclin-1 and Atg-5 (refs 29, 30) (Fig. 2a and Supplementary Fig. 7a). This had no effect on cell viability by itself, but significantly increased the sensitivity of MCF7 and T47D cells to palbociclib (Fig. 2b and Supplementary Fig. 7b,c). Specifically, Beclin-1 or Atg-5 knockdown induced irreversible growth inhibition, irreversible G1 arrest and elevated senescence, even in cells treated with low concentrations of palbociclib (Fig. 2c and Supplementary Fig. 7d–f), suggesting that ablation of autophagy significantly augments the drug’s ability to induce senescence.

We next interrogated whether pharmacological inhibition of autophagy would synergize with palbociclib to induce senescence. Treatment with the autophagy inhibitor HCQ, in combination with low-dose palbociclib induced long-term sustained growth inhibition, irreversible G1 arrest, significant increase in ROS levels and cellular senescence, without inducing apoptosis, when compared to palbociclib alone (Fig. 2d–g and Supplementary Fig. 8a–g). This suggests that autophagy degrades ROS, preventing the induction of senescence at low concentrations (Supplementary Fig. 8j). Moreover, in MCF7 and T47D cells with CDK4 and CDK6 knocked down via siRNA and shRNA, HCQ treatment significantly increased growth inhibition compared to CDK4/6 knockdown alone, validating the synergy between CDK4/6 and autophagy inhibition (Fig. 2h and Supplementary Fig. 8h,i).

Next, we examined whether the other clinically available CDK4/6 inhibitors, abemaciclib and ribociclib (Supplementary Fig. 9a,b) could also synergize with autophagy inhibition. Results revealed that combined treatment with ribociclib or abemaciclib and HCQ yielded significant sustained growth inhibition (Fig. 2i,k and Supplementary Fig. 9c,d). Unlike palbociclib, co-treatment with abemaciclib and HCQ also induced significant apoptosis when compared to abemaciclib alone (Fig. 2j and Supplementary Fig. 9e). Finally, four additional autophagy-inhibiting drugs, Lys05 (ref. 31), CQ, bafilomycin A1 (ref. 32) and spautin-1 (ref. 33) (Supplementary Fig. 9f), all synergized with low-dose palbociclib, resulting in irreversible growth inhibition (Fig. 2l–n and Supplementary Fig. 9g–k).

Taken together, these results demonstrate that inhibition of autophagy via genetic ablation or pharmacological agents induces a synergistic response in combination with a CDK4/6 inhibitor, resulting in sustained growth inhibition and senescence (or apoptosis in the case of abemaciclib).

### Palbociclib synergizes with autophagy inhibition *in vivo*

**Figure 3:**
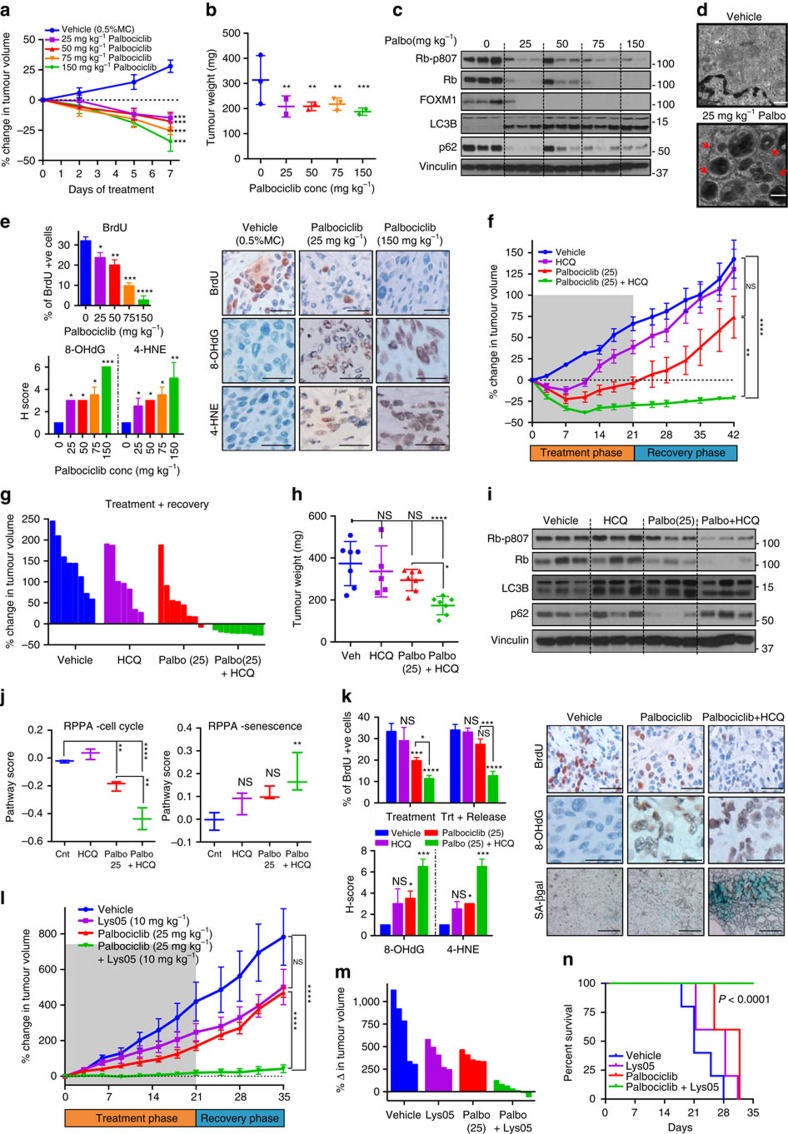
Palbociclib synergizes with autophagy inhibition *in vivo*. (**a**) Percentage change in tumour volume (normalized to Day 0) upon treatment with vehicle or palbociclib (*n*≥4 tumours per group) daily for 7 days. Tumours were then harvested for (**b**) Tumour weight measurement, (**c**) western blot of cell cycle (phospho-Rb, Rb, FOXM1) and autophagy (LC3B, p62) proteins, (**d**) TEM analysis (red arrows indicate double-membraned autophagosomes; Scale bars, 500 nm) and (**e**) BrdU, 8-OHdG and SA-ß gal staining (Scale bars, 50 μm). (**f**,**g**) Percentage change in mean (**f**) or individual (**g**) tumour volumes (normalized to Day 0) upon treatment with Vehicle, 25 mg kg^−1^ palbociclib, 60 mg kg^−1^ HCQ or combination of palbociclib (25 mg kg^−1^) and HCQ daily for 21 days (treatment phase) and recovery for 21 days (recovery phase) (*n*≥8 tumours per group). Data represented as mean±s.e.m. Tumours were harvested for (**h**) tumour weight measurement at end of treatment+recovery phase (*n*≥6 for each group), (**i**) western blot of cell cycle (phospho-Rb, Rb) and autophagy (LC3B, p62) proteins, (**j**) RPPA analysis and Pathway score of proteins in the cell cycle (*n*=10) and senescence (*n*=13) pathways (Error bars represent maximum and minimum values) and (**k**) BrdU, 8-OHdG and SA-ß gal staining with representative images end of recovery phase and quantitation (Scale bars, 50 μm). (**l**,**m**) Percentage change in mean (**l**) or individual (**m**) tumour volumes (normalized to Day 0) upon treatment with Vehicle, 5 mg kg^−1^ palbociclib, 10 mg kg^−1^ Lys-05 or combination of palbociclib and Lys-05daily for 21 days (treatment phase) and recovery phase of 14 days. Data represented as mean±s.e.m. *n*≥5 for each group. (**n**) Kaplan–Meier survival curve with death and tumours exceeding 1,000 mm^3^ as end point upon treatment as in (**l**). *n*≥5 for each group. All data are represented as mean±s.d. and *P* values were calculated in comparison to mice treated with vehicle unless indicated. NS: *P*>0.05; *P<0.05; **P<0.01; ***P<0.001; ****P<0.0001.

To interrogate whether palbociclib induces autophagy *in vivo*, we treated mice with MCF7-T cell line (MCF7 cells passaged through mice and verified to behave similarly to MCF7 cells; Supplementary Fig. 10a–f) orthotopic xenografts with increasing concentrations (25–150 mg kg^−1^ per day) of palbociclib for 7 days (Supplementary Fig. 10g). Palbociclib treatment significantly decreased tumour volume (Fig. 3a,b and Supplementary Fig. 10h), while increasing ROS (measured by 8-hydroxydeoxyguanosine^34^ and 4-hydroxynonenal^35^) and SA-ß gal activity, and lowering expression of bromo-2′-deoxyuridine (BrdU), pRb and FOXM1 in a dose-dependent manner (Fig. 3c,e and Supplementary Fig. 10i). Consistently, RPPA analysis of xenograft tumours showed dose-dependent downregulation of cell cycle proteins and upregulation of senescence proteins (Supplementary Fig. 10l–n). While treatment with palbociclib at 25 and 50 mg kg^−1^ was well tolerated, mice treated with 75 or 150 mg kg^−1^ palbociclib exhibited significant loss in body weight, suggesting potential toxicity at these concentrations and emphasizing the need to optimize the utility of lower doses (Supplementary Fig. 10j,k). Notably, western blot, RPPA and TEM analysis of the tumours treated with the lowest dose (25 mg kg^−1^ per day) of palbociclib showed higher levels of LC3B-II, Atg-7 and lower levels of p62, as well as the presence of double-membrane electron-dense autophagosomes (Fig. 3c,d and Supplementary Fig. 10l,o–q), demonstrating the induction of intact autophagy.

We next interrogated the synergy between autophagy inhibitor and low-dose palbociclib by treating xenograft tumour-bearing mice with (i) vehicle, (ii) HCQ (60 mg kg^−1^ per day), (iii) low-dose palbociclib (25 mg kg^−1^ per day) or (iv) palbociclib+HCQ for 21 days (treatment phase) with an additional recovery period of 21 days (recovery phase; Supplementary Fig. 11a). The mice treated with the combination of palbociclib+HCQ had significantly smaller tumour volumes, which remained small even after treatment was stopped, while the tumour volumes in the other arms increased during both treatment and recovery phases (Fig. 3f–h and Supplementary Fig. 11b–d). This suggests that co-treatment with HCQ enabled the induction of sustained tumour growth inhibition even at a palbociclib dose that is one-fifth of that (150 mg kg^−1^) used in most studies^36^,^37^,^38^. Moreover, palbociclib+HCQ *in vivo* treatment resulted in significantly higher ROS (8-OHdG, 4-HNE) and SA-ß gal activity, and decreased BrdU and pRb expression at the end of both treatment and recovery phases, while palbociclib alone exhibited changes only at the end of the treatment phase (Fig. 3i,k and Supplementary Fig. 11f,g). RPPA analysis of the tumours demonstrated a significant decrease in cell cycle and an increase in senescence proteins in the tumours of mice that received the combination compared to those that received no treatment or palbociclib only (Fig. 3j and Supplementary Fig. 11e). Further, the drug combination increased LC3B-II levels with no decrease in p62, compared to palbociclib alone, confirming the inhibition of autophagic flux by HCQ (Fig. 3i). Finally, mice that received palbociclib+HCQ showed no significant changes in body weight or blood counts, suggesting that this combination is well tolerated (Supplementary Fig. 11h–k).

To further confirm the synergy *in vivo,* we utilized another autophagy inhibitor, Lys05 (ref. 31) (a more potent inhibitor of autophagy compared to HCQ), which showed no significant toxicity as a single agent (Supplementary Fig. 12a–d). Tumour-bearing mice were treated with vehicle, 10 mg kg^−1^ per day Lys05, 25 mg kg^−1^ per day palbociclib or the combination of palbociclib and Lys05 for 21 days (treatment phase) with a recovery phase of 14 days. Treatment with the combination of palbociclib+Lys05 significantly decreased tumour volume during both the treatment and recovery phases, resulting in significantly smaller tumours and prolonged survival compared to vehicle or single-treatment controls (Fig. 3l–n and Supplementary Fig. 12e–g).

Collectively, these results demonstrate that autophagy inhibition synergizes with low doses of palbociclib to induce irreversible tumour growth inhibition *in vivo*.

### G1/S checkpoint predicts response to palbociclib and HCQ

**Figure 4:**
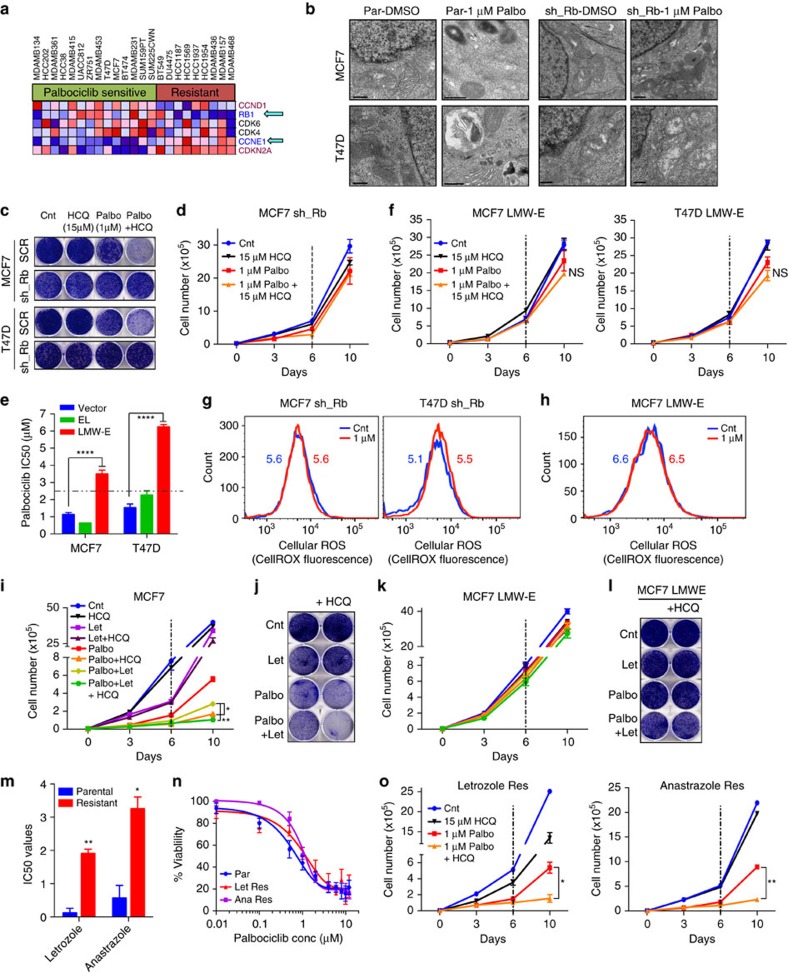
G1/S checkpoint predicts response to combination of palbociclib and autophagy inhibition. (**a**) Heat map constructed using the Gene Set Enrichment Analysis program, denoting expression of genes from the Biocarta_Cell_Cycle gene set. (**b**) Representative TEM microphotograph of parental (Par) and Rb-knockdown MCF7 and T47D cells treated with DMSO or 1 μM palbociclib for 6 days. Scale bars, 500 nm. Rb-knockdown MCF7 and T47D cells were treated with the combination of 1 μM palbociclib and 15 μM HCQ for 6 days, allowed to recover for 6 or 4 days and subjected to (**c**) Clonogenic assay and (**d**) Cell counting. MCF7 and T47D cells overexpressing vector (Vec), full-length cyclin E (EL) or LMWE were treated with palbociclib for 6 days and subjected to (**e**) Dose–response studies to measure Palbociclib IC_50_ values and (**f**) Cell counting in combination with 15 μM HCQ. (**g**,**h**) Measurement of cellular ROS levels and quantification (MFI; numbers on graph) in Rb-knockdown (**g**) LMWE overexpressing (**h**) MCF7 and T47D cells treated palbociclib for 6 days. (**i**,**j**) Cell counting (**i**) and clonogenic assay (**k**) to assess proliferation upon treatment with 1 μM palbociclib, 1 μM letrozole and 15 μM HCQ in the indicated combinations. (**k**,**l**) Impact of overexpressing LMWE in MCF7 aromatase-expressing cells measured by cell counting (**k**) and clonogenic assay (**l**) following treatment with 1 μM palbociclib, 1 μM letrozole and 15 μM HCQ in the indicated combinations. (**m**) IC_50_ values of letrozole and anastrazole in parental versus letrozole or anastrazole resistant cells, respectively. MCF7 parental, letrazole and anastrazole resistant cells were treated with palbociclib for 6 days and subjected to (**n**) Dose–response studies to measure Palbociclib IC_50_ values and (**o**) Cell counting in combination with 15 μM HCQ. All data represent mean±s.d. from three independent experiments; *P* values were calculated in comparison to cells treated with DMSO (Control) or parental unless indicated. NS: *P*>0.05; *P<0.05; **P<0.01; ***P<0.001; ****P<0.0001.

To identify biomarker(s) of response to palbociclib and the palbociclib+HCQ combination, we examined the gene expression of cell lines known to be sensitive or resistant to palbociclib^7^ (Supplementary Fig. 13a,b). This produced a distinct signature within the Biocarta_Cell_Cycle Pathway gene set, with a striking correlation between palbociclib sensitivity and expression of Rb and cyclin E (Fig. 4a), leading us to hypothesize that these proteins may be effective biomarkers to predict response to palbociclib and the combination of CDK4/6 and autophagy inhibitors.

Ablation of Rb in MCF7 and T47D cells (Supplementary Fig. 13c,d) reduced sensitivity to palbociclib, mediating resistance to the induction of growth inhibition, G1 arrest and senescence (Supplementary Fig. 13e–l). Further, knockdown of Rb resulted in no significant increase in MDC staining or autophagic vesicles with palbociclib, and no growth-inhibitory effect upon treatment with HCQ (Fig. 4b–d and Supplementary Fig. 13m,n), demonstrating a role for Rb in palbociclib-induced autophagy. Since Rb loss is not common in ER+ breast cancer, and hence may not be sufficient to predict patient response to palbociclib^39^, we also interrogated the effect of cyclin E. We have previously shown that post-translational modification (cleavage) of full-length cyclin E generates an oncogenic form termed low-molecular-weight isoforms of cyclin E (LMWE)^40^, which is uniquely expressed in tumours, hyperphosphorylates Rb, mediates resistance to letrozole and is a strong prognostic indicator in breast cancer^41^,^42^,^43^,^44^. Over-expression of LMWE, but not empty vector or full-length cyclin E (Supplementary Fig. 14a), significantly reduced sensitivity to palbociclib, and mediated resistance to palbociclib-induced growth inhibition, G1 arrest, and senescence (Fig. 4e and Supplementary Fig. 14b–h). Moreover, treatment with HCQ had no impact on the growth of palbociclib-treated LMWE-overexpressing cells (Fig. 4f and Supplementary Fig. 14i). Finally, knockdown of Rb or induction of LMWE abolished the ability of low-dose (1 μM) palbociclib to induce an increase in cellular ROS levels (Fig. 4g,h and Supplementary Fig. 14j–l). This suggests that intact Rb and cyclin E are required for palbociclib-induced senescence, autophagy and ROS (Supplementary Figs 13o, 14m).

Since palbociclib is currently used clinically in combination with letrozole, we next interrogated how this compares to our proposed combination of palbociclib+HCQ. Treatment of MCF7 aromatase-expressing cells with palbociclib+HCQ had a significantly higher growth-inhibitory effect compared to palbociclib and letrozole alone or in combination (Fig. 4i,j). Moreover, treatment with palbociclib+letrozole also induced autophagy (Supplementary Fig. 15a), making the triple combination of palbociclib, letrozole and HCQ more effective than palbociclib+letrozole (Fig. 4i,j). Further, over-expression of LMWE, but not empty vector-mediated resistance to the combination of palbociclib+letrozole and the triple drug combination (Fig. 4k,l and Supplementary Fig. 15b–d).

Finally, we examined the effect of palbociclib and autophagy inhibition in cells made resistant to aromatase inhibitors, letrozole and anastrazole. Cells that are ten-fold more resistant to letrozole and anastrazole (Fig. 4m) remained sensitive to palbociclib as a single agent (Fig. 4n and Supplementary Fig. 15e,f), induced autophagy in response to palbociclib, and were responsive to the combination of palbociclib and autophagy inhibition (Fig. 4o and Supplementary Fig. 15g,h), highlighting the clinical utility of the drug combination in the aromatase inhibitor resistance setting.

Taken together, these results reveal the importance of an intact G1/S transition for palbociclib response and provide a rationale for utilizing Rb in combination with LMWE as biomarkers of response to the combination of CDK4/6, aromatase and autophagy inhibitors in ER+ breast cancer.

### Palbociclib synergizes with autophagy inhibition in solid tumours

**Figure 5:**
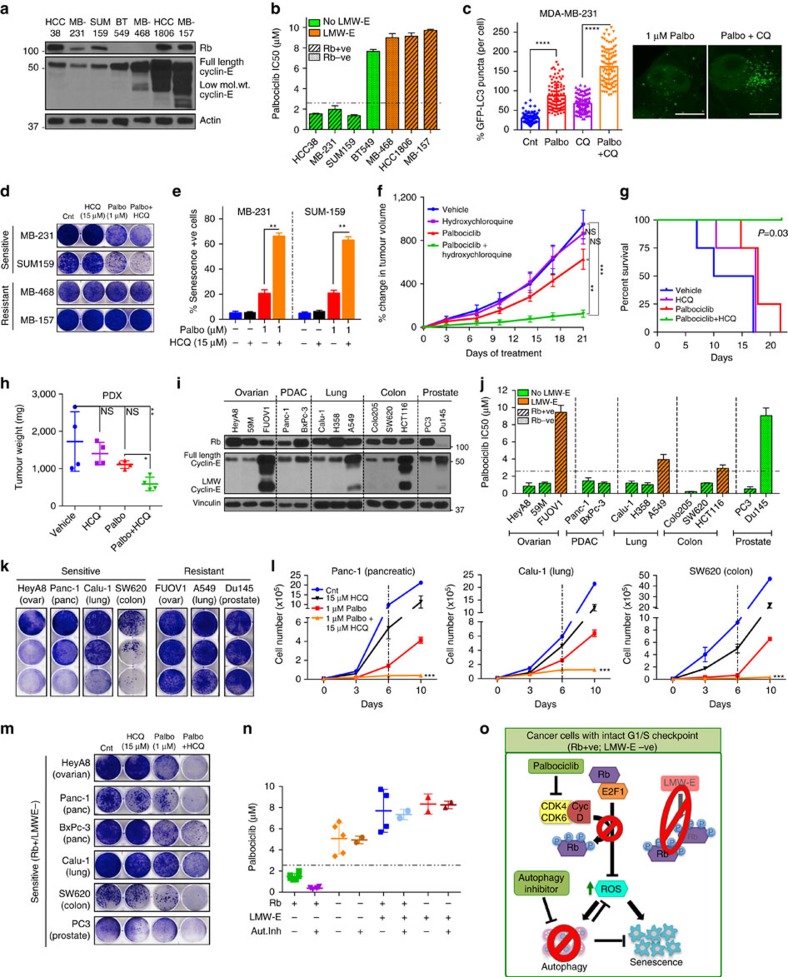
Synergism between palbociclib and autophagy inhibition in other solid tumours. (**a**) Western blot for Rb and Cyclin E in TNBC cell lines. (**b**) IC_50_ values from drug response experiments in TNBC cell lines treated with palbociclib for 6 days. (**c**) Quantification of GFP-LC3 puncta and representative images in MDA-MB-231 cells treated with 1 μM palbociclib and/or 25 μM CQ for 48 h. TNBC cell lines were treated with combination of 1 μM palbociclib and 15 μM HCQ for 6 days and subjected to (**d**) Clonogenic and (**e**) SA-ß galactosidase assays. (**f**) Percentage change in volume (normalized to Day 0) of PDX tumours upon treatment with Vehicle, 25 mg kg^−1^ palbociclib, 60 mg kg^−1^ HCQ or combination of palbociclib (25 mg kg^−1^) and HCQ daily for 21 days. Data represented as mean±s.e.m. *n*=4 for each group. (**g**,**h**) Kaplan–Meier survival curve with death and tumours exceeding 1,000 mm^3^ as end point (**g**) and weight (**h**) of PDX tumours treated as in (**f**). *n*=4 for each group. (**i**) Western blot of Rb and Cyclin E in ovarian, pancreatic (PDAC), lung, colon and prostate cancer cell lines. (**j**,**k**) IC_50_ values from drug response experiments (**j**) and Clonogenic assay (**k**) in the mentioned cancer cell lines treated with palbociclib for 6 days and recovery for 6 days. (**l**,**m**) Cell counting (**l**) and clonogenic assay (**m**) in cancer cell lines treated with 1 μM palbociclib and 15 μM HCQ for 6 days and recovery for 4 or 6 days respectively. *P* value calculated in comparison with 1 μM palbociclib. (**n**) Correlation between palbociclib IC_50_ values (from dose–response studies in all cancers) and levels of Rb and cyclin E proteins, with and without inhibition of autophagy (Beclin-1/Atg5 knockdown or HCQ treatment). (**o**) Schematic depicting the mechanism by which palbociclib inhibits growth of Rb+/LMWE− breast cancer cells by regulating ROS, autophagy and senescence. All data represent mean±s.d. from three independent experiments; NS: *P*>0.05; *P<0.05; **P<0.01; ***P<0.001; ****P<0.0001.

We next asked whether autophagy inhibition would be synergistic with palbociclib in ER−ve breast cancer and other solid tumours, and if LMWE and Rb can predict drug response. To this end, we first examined seven triple-negative breast cancer (TNBC) cell lines for their expression of Rb and LMWE (Fig. 5a). The Rb+ and LMWE− cell lines (HCC38, MDA-MB-231, SUM-159) were significantly more sensitive to palbociclib (Fig. 5b and Supplementary Fig. 16a–c) than the Rb− or LMWE+ cell lines, mediating dose-dependent sustained growth inhibition, G1 arrest and senescence (Supplementary Fig. 16d–h). Moreover, palbociclib treatment-induced autophagy with an active flux in Rb+/LMWE− TNBC cells (Fig. 5c and Supplementary Fig. 17a,b). Treatment with the autophagy inhibitor HCQ further sensitized Rb+/LMWE− cells to palbociclib, inducing irreversible growth inhibition and elevated senescence even at low doses (Fig. 5d,e and Supplementary Fig. 17d). In contrast, cell lines with a deregulated G1/S checkpoint displayed resistance to palbociclib-mediated senescence and autophagy, and the combination of palbociclib and HCQ (Fig. 5a,b,d and Supplementary Figs 16, 17a–d).

To examine this synergy between palbociclib and autophagy inhibition *in vivo*, we treated Rb+ LMWE− TNBC patient-derived xenograft (PDX) tumours with vehicle, HCQ, palbociclib (25 mg kg^−1^) or palbociclib+HCQ (Supplementary Fig. 17e). Treatment with the drug combination significantly decreased tumour growth and prolonged survival compared to vehicle or either drug alone (Fig. 5f–h and Supplementary Fig. 17f–h).

While palbociclib is currently approved only for ER+/Her2− breast cancer, numerous studies have shown that CDK4/6 inhibitors have activity in other cancers^5^,^37^,^38^. To examine whether the combination of palbociclib and autophagy inhibitor is effective in other solid tumours, we examined several ovarian, pancreatic, lung, colorectal and prostate cancer cell lines with varied expression levels of Rb and cyclin E (Fig. 5i). Only cell lines with intact Rb and no LMWE (HeyA8, 59M, Panc-1, BxPc-3, Calu-1, H358, Colo-205, SW-620 and PC3) exhibited significant dose-dependent growth inhibition in response to palbociclib (Fig. 5j,k and Supplementary Fig. 18a,b). Moreover, treatment with the autophagy inhibitor further sensitized the Rb+/LMWE− cell lines to palbociclib (1 μM) and induced irreversible growth inhibition (Fig. 5l,m and Supplementary Fig. 18c,d).

Correlation between the half-maximal inhibitory concentration (IC_50_) of palbociclib and expression of Rb and LMWE across all cancer cell lines revealed that cell lines with an intact G1/S transition (Rb+/LMWE−) were significantly more sensitive to the CDK4/6 inhibitor and that this sensitivity was further increased by autophagy inhibition (Fig. 5n and Supplementary Fig. 18e). Taken together, these results demonstrate that the combination of CDK4/6 and autophagy inhibitors can be utilized to effectively treat solid tumours, with Rb and cyclin E as biomarkers of response (Fig. 5o).

### Rb and cytoplasmic cyclin E: prognosticators of palbociclib in breast cancer

**Figure 6:**
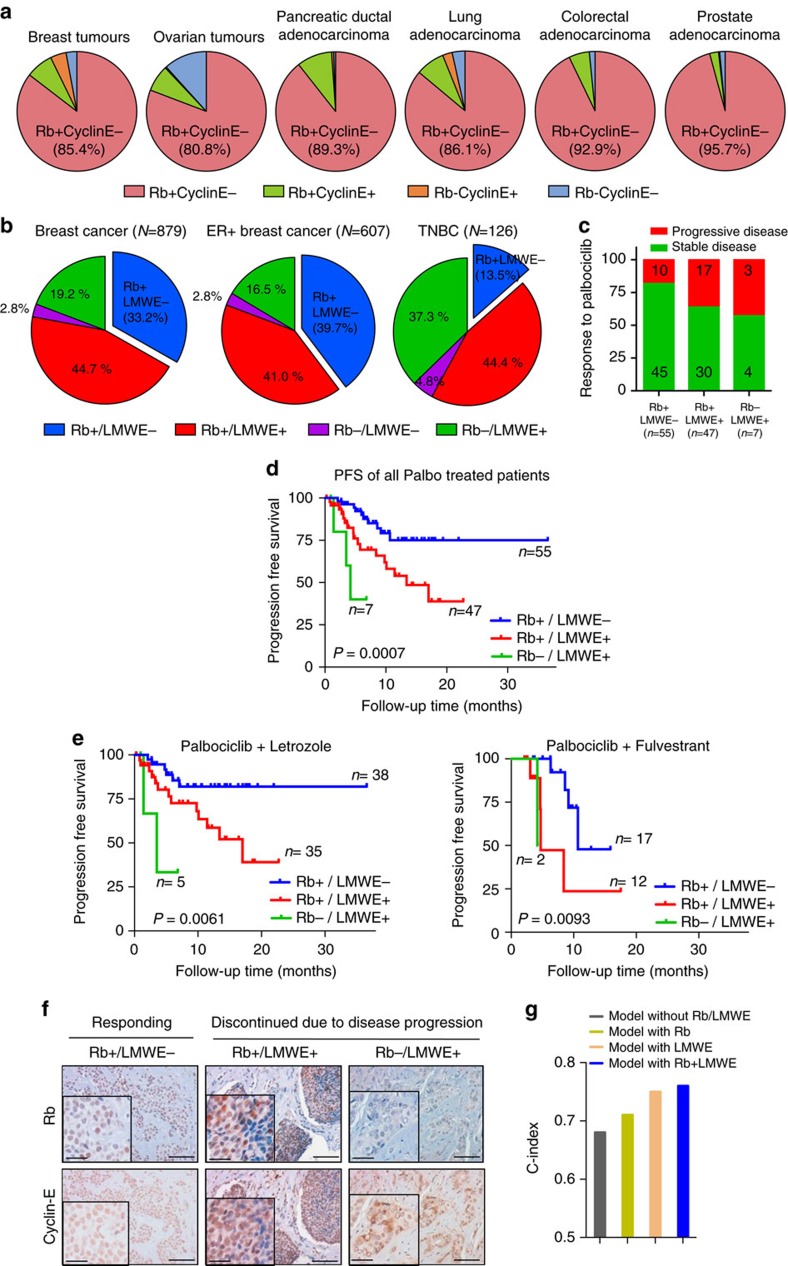
Rb and LMWE are prognostic markers of palbociclib in breast cancer patients. (**a**) Alterations in Rb and cyclin E RNA levels in tumours from patients with breast (*n*=817), ovary (*n*=557), lung (*n*=230), pancreas (*n*=178), colon (*n*=379) or prostate (*n*=333) cancer taken from the TCGA database. (**b**) Percentage of breast cancer patients exhibiting alterations in Rb and LMWE as determined via immunohistochemical staining of tumour samples from the NCI patient cohort (*n*=879). (**c**) Proportion of 109 breast patients treated with palbociclib with Rb+/LMW−, Rb+/LMWE+ or Rb−/LMWE+ status and their disease progression (response to palbociclib). (**d**,**e**) Kaplan–Meier curves showing PFS duration (in months) among 109 patients with advanced ER+ breast cancer classified on the basis of their tumoral expression of Rb and cyclin E (**d**) and separated based on letrozole and fulvestrant (**e**). Survival curves were censored at disease progression or date of last follow-up. (**f**) Representative images from immunohistochemical analysis of Rb and LMWE in tumours from patients with ER+ breast cancer treated with palbociclib, classified on the basis of response. Scale bars equal 50 μm and insert scale bars equal 20 μm. (**g**) C-index of the multivariate cox model with progesterone receptor, prior therapy for metastatic disease only (without Rb) and the addition of Rb and LMWE.

TCGA analysis revealed that 85, 81, 89, 86, 93 and 96% of breast, ovarian, pancreatic, lung, colorectal and prostate cancers, respectively, are positive for Rb and have low/no expression of cyclin E (Fig. 6a and Supplementary Fig. 19a). However, these analyses are based on RNA levels, which do not differentiate between full-length cyclin E (nuclear) and LMWE (cytoplasmic), nor can accurately predict Rb protein expression (Supplementary Fig. 19b). For these studies we evaluated the subcellular localization of cyclin E and scored the samples either as cytoplasmic negative or positive. LMWE positive refers to cytoplasmic staining of cyclin E. Hence, we examined LMWE and Rb protein expression in tissue microarray samples from a cohort of 879 early stage breast cancer patients from the NCI Cancer Diagnosis Program^41^,^44^. LMWE and Rb staining of formalin-fixed paraffin-embedded slides from this cohort revealed that 33% of all tumour samples were Rb+ and LMWE−, while 40% of the ER+ subtype were Rb+/LMWE− (Fig. 6b, Supplementary Fig. 19c). Further, Kaplan–Meier curves showed that the patient groups with deregulated cyclin E (LMWE+) had worse overall prognosis, emphasizing the prognostic ability of LMWE in breast cancer (Supplementary Fig. 19d). These results suggest that over 35% of all breast cancer patients (regardless of ER status) may benefit from treatment with the palbociclib+HCQ combination, highlighting the clinical utility of this regimen.

Next, to directly test the utility of Rb and LMWE (cytoplasmic cyclin E) protein as prognostic biomarkers in metastatic breast cancer, we utilized a cohort of 109 patients with advanced ER+ breast cancer who were/are currently being treated with the combination of palbociclib and letrozole or fulvestrant at MD Anderson Cancer Center (Supplementary Fig. 20a). Archival, pre-treatment specimens from the primary tumour and/or local or metastatic recurrence were obtained from each patient and subjected to cyclin E and Rb staining and scoring (Fig. 6f and Supplementary Fig. 20d). The majority (102/109) of the samples were positive for Rb, while only 49.5% (54/109) were positive for LMWE (Supplementary Fig. 20b). Analysis of clinical and pathologic variables revealed that the patients with LMWE or Rb loss exhibited higher rates of progression at 6 and 12 months (assessed by Kaplan–Meier methods) with the heavily treated fulvestrant group experiencing greater progression rate at 12 months as expected (Supplementary Tables 1,2). Further, Rb and LMWE varied significantly with progression rates for the palbociclib+letrozole and palbociclib+fulvestrant treatment groups as single (Rb: *P*=0.02, LMWE: *P*=0.01 and Rb: *P*=0.03, LMWE: *P*=0.009, respectively) and combined variables (*P*=0.006 and *P*=0.009, respectively) (Supplementary Tables 1,2). Univariate Cox proportional hazards model showed that Rb and LMWE (as single and combined variables) were the only factors significantly associated with the progression-free interval with both palbociclib+letrozole and palbociclib+fulvetrant, with hazard ratios of 0.2 and 0.09 (Rb), 3.2 and 5.2 (LMWE), and 9.2 and 23.8 (Rb+LMWE), respectively (Supplementary Table 3). Further, 84.2% of Rb+/LMWE− exhibited stable disease in response to palbociclib+letrozole and 82.4% with palbociclib+fulvestrant, compared to 61.4 and 58.3% in the other patient groups, respectively (Fig. 6c and Supplementary Fig. 20c). Kaplan–Meier PFS plots for the 109-patient cohort separated by letrozole (*n*=78) or fulvestrant (*n*=31) treatment and Rb/LMWE expression revealed that the patients with Rb+ and LMWE− tumour had the longest PFS time (median >36.5 months overall and with letrozole; 10.7 months with fulvestrant), compared to Rb+/LMWE+ patients (median=13.4 months; 17 months with letrozole and 4.7 months with fulvestrant) and Rb−/LMWE+ patients who had the shortest PFS time (median=4.2 months; 3.5 months with letrozole and 4.2 months with fulvestrant) (Fig. 6d,e). The concordance indices (C-indices) for the multivariate model also showed substantial gains when Rb and LMWE were included as single or combined variables, compared to the model without Rb and cyclin E. (Fig. 6g and Supplementary Table 4). Collectively, these results provide evidence that LMWE and Rb are reliable prognostic biomarkers in advanced ER+ breast cancer patients.

## Discussion

We report here that cancer cells activate autophagy in response to palbociclib, and that blockade of autophagy significantly improves the efficacy of CDK4/6 inhibition *in vitro* and *in vivo* in cancers with an intact G1/S transition (Supplementary Fig. 21).

While research has shown opposing roles for autophagy—as a pro-survival and a pro-death mechanism—numerous recent studies have highlighted the importance of autophagy as a mediator of drug resistance, specifically in breast cancer^13^,^45^,^46^. These studies have shown an association between high expression of autophagy proteins like LC3B and tumour aggressiveness or residual disease post chemotherapy, thus providing strong rationale for using autophagy inhibitors to combat chemoresistance. Further, a recent study has shown that cyclin D1 can upregulate autophagy, which when downregulated, results in an increase in senescence^47^. Thus, results from our study corroborates these findings and provides strong *in vitro* and *in vivo* evidence that autophagy inhibitors can be utilized to combat resistance to cell-cycle-targeted therapies, such as CDK4/6 inhibitors.

Although our results show that CDK4/6 inhibition induces ROS, its molecular mechanism remains unclear. Cyclin D1 has been shown to bind to and phosphorylate Nrf1, a regulator of mitochondrial biogenesis and ROS, in a CDK-dependent manner^48^. Hence, it is possible that CDK4/6-cyclin D1 inhibition via palbociclib increases Nrf1 levels, thus increasing ROS activity. The levels of ROS and the subsequent induction of senescence, in turn, might be controlled by c-jun through a previously elucidated mechanism involving the ROS genes, MnSOD and catalase^49^. Alternatively, the induction of ROS might be mediated directly by the Rb targets FOXM1 and BIRC5 (survivin), which decrease in response to palbociclib and have been shown to negatively regulate oxidative stress^50^,^51^.

A recent study revealed that palbociclib has kinase targets apart from CDK4 and CDK6, namely PIK3CD and PIK3R4 (ref. 52). PIK3R4 (Vps15) is a class III phosphatidylinositol 3-kinase shown to be required for autophagic clearance of proteins. Defects in Vps15 lead to dysfunctional lysosomes^53^,^54^, similar to those observed in our study in response to high doses of palbociclib (5 μM or 150 mg kg^−1^). Hence, it is likely that palbociclib inhibits these secondary targets at higher concentrations, accounting for the disruption of autophagic flux observed at these doses, and the observed off-target effects with siRNA against CDK4/6. This hypothesis might also explain why treatment with other CDK4/6 inhibitors failed to elicit such a response, given that these secondary targets are unique to palbociclib^52^.

Identification of reliable biomarkers for palbociclib has proven challenging. While previous *in vitro* studies showed that Rb, cyclin D and p16 could predict response to palbociclib^55^,^56^,^57^, results from Phase II/III trials showed no significant correlation between drug response and the expression of p16 (ref. 2), Ki67, *CCND1* amplification^58^, *PIK3CA* or *ESR1* (ref. 59) mutational status, leaving no established prognostic or predictive biomarkers^6^. Here, we use a dual biomarker strategy and show that Rb and LMWE proteins are reliable prognostic biomarkers in advanced ER+ breast cancers. Future clinical trial investigations in early stage breast cancer patients, in the neoadjuvant setting, where patients are treated with either palbociclb+letrozole or letrozole alone, would reveal the predictive utility of these proteins for palbociclib treatment. Thus, we propose that a simple immunohistochemical assay for Rb and LMWE can be used clinically to identify patients who are likely to have a response to palbociclib and its combination with autophagy inhibitor.

While previous preclinical studies have shown that a small percentage of TNBC cell lines respond to palbociclib^7^,^60^, this has been overlooked thus far, possibly because of the lack of a definitive biomarker to select TNBC patients. The *in vitro* and *in vivo* data presented here provide a rationale to expand the scope of CDK4/6 inhibitor treatment to TNBC patients, given that Rb and LMWE are used to identify the potentially most responsive patients.

We also propose future clinical trials that are biomarker integrated and utilize HCQ (or other autophagy inhibitors such as Lys05) to potentiate the action of palbociclib. Given that HCQ is well tolerated and is currently in clinical trials to reverse hormonal and cytotoxic drug resistance^14^,^61^, we first propose a Phase II clinical trial (in the neoadjuvant setting) in postmenopausal advanced ER+/HER2− breast cancer patients, treating them with the combination of low-dose palbociclib (75 mg kg^−1^ per day), HCQ and letrozole using surrogate biomarkers such as Ki67 staining. Eligible patients will be selected on the basis of Rb and LMWE expression assessed from baseline biopsy specimens. Results from this clinical trial would support initiation of a definitive Phase III trial for the triple combination, possibly even extending this treatment to other tumours. This would also support the utility of palbociclib+HCQ combination to treat RB+/LMWE− ER+ breast cancer in the neoadjuvant setting. We predict that the combination of continuous low-dose palbociclib and HCQ would be more beneficial than standard-dose palbociclib (21 days on, 7 days off), allowing us to minimize palbociclib-mediated toxicities, avoid proliferative bursts that occur when palbociclib is stopped^62^ and prolong overall patient survival—a goal that has not yet been met with currently approved palbociclib treatment combinations.

In summary, this study addresses the current limitations of CDK4/6 inhibitor treatment and proposes a biomarker-driven clinical trial that evaluates the ability of autophagy inhibitors to improve the selectivity and efficacy of CDK4/6 inhibitors in clinic.

## Methods

### Cell lines

All cell lines used in this study were obtained from ATCC. MCF7, T47D, ZR75-1, MCF7-T, MDA-MB-231, HCC38, HCC1806, MDA-MB-157, HeyA8, 59M and FUOV1 were maintained in minimum essential medium Eagle alpha modification (alpha MEM) supplemented with 10% fetal bovine serum (FBS), 10 mM HEPES, nonessential amino acids, 2 mM l-glutamine, sodium pyruvate and hydrocortisone. 293T and MDA-MB-468 cells were cultured in Dulbecco modified Eagle medium (DMEM) supplemented with 10% FBS. SUM-159 and BT-549 cells were cultured in a 1:1 mixture of alpha MEM and nutrient F-12 Ham medium supplemented with 10% FBS and 5% insulin. Calu-1, H358, A549, PC3 and Du145 cells were cultured in RPMI-1640 medium supplemented with 10% FBS. The pancreatic cancer cell lines (Panc-1 and BxPC-3) were a gift from the laboratory of Dr Anirban Maitra (MD Anderson Cancer Center) and were cultured in DMEM and RPMI 1640, respectively, supplemented with 10% FBS. The colon cancer cell lines (Colo-205, SW-620 and HCT-116) were obtained from the laboratory of Dr Jae-II Park (MD Anderson Cancer Center) and cultured in DMEM supplemented with 10% FBS.

The MCF7 aromatase-expressing cells were maintained in DMEM supplemented with 10% tet-free FBS + 4 μg ml^−1^ blasticidin, 1 μg ml^−1^ puromycin (InvivoGen, San Diego, CA) and 400 μg ml^−1^ G418. Before experimental set-up, they were estrogen deprived for 4 days using phenol red free IMEM supplemented with 10% charcoal-dextran-treated tet-free FBS. During the experiment, the cells were maintained in estrogen deprivation condition in the presence of 25 nM 4-Androstene-3,17-dione. To induce the expression of empty vector and LMWE, the cells were treated with 5 ng ml^−1^ Doxycyclin (Sigma) for 24 h before the start of the experiment. The aromatase resistant cell lines were maintained in phenol red DMEM supplemented with 10% charcoal-dextran-treated FBS+25 nM 4-Androstene-3,17-dione+400 μg ml^−1^ G418. All cell lines were maintained free of mycoplasma contamination and authenticated on a regular basis by karyotype and short tandem repeat analysis at MD Anderson’s Characterized Cell Line core facility.

### Antibodies and drugs

Antibodies against p-Rb (Ser807/811 - #9308, 1:1,000), FOXM1 (#3948, 1:1,000), total Rb (4H1 - #9309, 1:1,000), PARP (#9542, 1:1,000), caspase-7 (#9492, 1:1,000), LC3B (#2775, 1:1,000), p62/SQSTM1 (#5114, 1:1,000), Beclin-1 (#3738, 1:1,000), Atg-5 (#2630, 1:1,000) and p53 (#9282, 1:1,000) were purchased from Cell Signaling Technology (Danvers, MA). Antibodies against CDK4 (C-22—sc260, 1:1,000), CDK6 (C-21—sc177, 1:1,000), cyclin E (HE-12—sc247,1:200) and Mdm2 (sc812, 1:1,000) were purchased from Santa Cruz Biotechnology (Dallas, TX); antibodies against actin (C-4—MAB1501, 1:5,000) and vinculin (V9131, 1:10,000) were purchased from Millipore and Sigma-Aldrich (St Louis, MO), respectively.

Palbociclib was obtained from Pfizer, Inc (San Diego, CA). It was diluted in dimethyl sulfoxide (DMSO) *for in vitro* use and in 0.5% methylcellulose (Sigma-Aldrich, St Louis, MO) for *in vivo* use and administered to mice via oral gavage. HCQ sulfate was purchased from Sigma-Aldrich and Selleckem. Lys-05 was provided by Dr Ravi Amaravadi (University of Pennsylvania). HCQ and Lys-05 were diluted in sterile water for *in vitro* use and in sterile phosphate-buffered saline solution (PBS) for *in vivo* use and administered to mice via intraperitoneal injection. N-acetyl-L-cysteine (NAC), trolox [(±)-6-hydroxy-2,5,7,8-tetramethylchromane-2-carboxylic acid], bafilomycin A1, spautin-1 and CQ diphosphate were all purchased from Sigma-Aldrich. Ribociclib was purchased from Selleckem and abemaciclib from MedChem Express; NAC and CQ were diluted in sterile water. Trolox, bafilomycin A1, spautin-1, ribociclib and abemaciclib were diluted in DMSO.

### siRNA knockdown

siRNA knockdown of CDK4 and CDK6 were generated as described previously^63^. ON-TARGETplus SMARTpool siRNA for CDK4 (L-003238-00-0005) and CDK6 (L-003240-00-0005) were purchased from Dharmacon. Briefly, cells were plated on 6-well or 12-well plates and tranfected with siRNA targeting CDK4 and/or CDK6 using JetPRIME transfection reagent (Polyplus transfection, New York, NY), as per manufacturer’s protocol. Non-coding siRNA pool (siNT) was used as a negative control for comparison. Cells were collected 72 h post transfection for western blot analysis, MDC analysis, CellROX assay and on days 3 and 6 for cell counting.

### shRNA knockdown and cyclin E over-expression

Stable shRNA knockdown cells were generated as described previously^63^. All shRNA constructs were purchased from Thermo Scientific (Open Biosystems, Waltham, MA). For single knockdown of CDK4, CDK6, Beclin-1, Atg-5 or Rb, GIPZ lentiviral shRNA was used [CDK4: V3LHS_641689, V3LHS_641690; CDK6: V2LHS_112906, V3LHS_404083; Beclin-1: V3LHS_349514, V3LHS_349513; Atg-5: V2LHS_67978, V3LHS_301131; Rb: V2LHS_130611, V2LHS_130606, V3LHS_340829]. For dual knockdown of CDK4 and CDK6, TRIPZ inducible shRNA for CDK6 was utilized for better selection of knockdowns. To generate lentivirus-expressing shRNA, HEK 293T cells were transfected with pCMVdeltaR8.2, pMD2.G (produced by the Didier Trono laboratory and made available through the Addgene repository) and pGIPZ vector (scrambled shRNA or shRNA against gene of interest) using LipoD293 (SignaGen) or polyethylenimine transfection reagent according to manufacturer’s protocol. After 48 h of transfection, the virus-containing medium was collected, filtered through a 0.45-μm filter and added to the cells of interest in the presence of 8 μg ml^−1^ of polybrene (Millipore). GFP or RFP expression was confirmed and the lentivirus-infected cells were selected with 1 μg ml^−1^ puromycin (InvivoGen, San Diego, CA). MCF7 and T47D cells overexpressing different isoforms of cyclin E (vector, full-length cyclin E (EL) or LMWE) were generated previously^64^ and verified in this study by western blot.

### Dose–response studies

For dose–response studies, 1,000 to 3,000 cells (depending on plating efficiency of each cell line;) were plated in each well of a 96-well plate and treated with increasing concentrations (0.01–12 μM) of palbociclib, ribociclib or abemaciclib for 1, 2, 4, 6 or 8 days. The medium was replaced with drug-containing medium every other day. At completion of drug treatment, cultures were continued in drug-free medium (also replaced every other day) until day 12, after which they were stained with 0.5% crystal violet solution. The plates were then solubilized with a solution of 0.1% sodium citrate in 50% ethanol, and absorbance was measured at 570 nm using the Epoch Microplate Spectrophotometer (BioTek Instruments, Inc, Winooski, VT). Values were normalized to those of their no treatment controls and analysed in GraphPad Prism by non-linear regression to obtain the IC_50_ values as used in Fig. 1a.

### Cellular proliferation assay

For cell proliferation studies, 7,500 to 15,000 cells (depending on plating efficiency of each cell line; data not shown) were plated in each well of six-well plates and treated with the indicated agents for 6 days and cells were allowed to recover for 4 days in the absence of drug to examine reversibility. The medium was replaced every other day during the course of the experiment, either with drug-containing medium (days 2 and 4) or drug-free medium (days 6 and 8). Cells were then collected and counted using the BioRad TC20 Automated Cell Counter on days 0, 3, 6 and 10.

For clonogenic-/colony-formation assay, 5,000 to 10,000 cells (depending on the plating efficiency of each cell line; data not shown) were plated in each well of six-well plates, treated for 6 days and allowed to recover for 6 days in the absence of drug. Cells were then washed with PBS and stained with a 0.5% crystal violet solution in 25% methanol for 10 min. Plates were then scanned to obtain pictures.

### Cell cycle and BrdU analyses

Cells were plated (1 × 10^5^ cells per plate) on 10-cm plates and treated with the indicated agents for 6 days; they were allowed to recover without drug(s) for 4 days to examine reversibility. Medium was replaced every other day during the course of the experiment, either with drug-containing medium (days 2 and 4) or drug-free medium (days 6 and 8). For cell cycle analysis, following treatment or treatment (6 days) and recovery (6 days+4 days of recovery), cells were subjected to trypsinization, washed with PBS, and fixed with 3.5 ml ice-cold PBS and 1.5 ml of 95% ethanol. Cells were prepared as described previously^63^ and incubated in a solution of propidium iodide (1 mg ml^−1^) and RNAase (1 mg ml^−1^) at 4 °C overnight. Samples were then analysed on the Beckman Coulter Gallios Flow Cytometer, and data were analysed with the Kaluza software (Beckman Coulter) after excluding doublet cells.

For BrdU analysis, following treatment or recovery, cells were incubated with 10 μM 5-BrdU (Sigma-Aldrich) for 1 h, after which they were subjected to trypsinization, washed with PBS and fixed with 70% ethanol. Cells were then washed with PBS containing 0.5% bovine serum albumin and denatured with a solution of 2 M HCl + 0.5% Triton X-100 at room temperature for 20 min. Cells were washed again and incubated in 200 μl 0.1 M sodium borate, pH 8.5, to neutralize any residual acid for 2 min at room temperature. Cells were then stained with fluorescein isothiocyanate-conjugated anti-BrdU (BD Biosciences) for 20 min at room temperature, protected from light. Cells were finally washed and incubated in a solution of 0.5 ml propidium iodide (10 μg ml^−1^ in PBS) for 30 min at room temperature, protected from light. Samples were analysed on the Beckman Coulter Gallios Flow Cytometer and data were analysed with Kaluza software to obtain the percentages of BrdU-positive cells.

### Measurement of senescence

For *in vitro* studies, senescence was measured by the SA-ß gal staining kit (Millipore, Billerica, MA) according to the manufacturer’s standard protocol. Briefly, cells were plated at a low density of 2,000 to 4,000 cells (depending on the plating efficiency of the cell line; data not shown) in each well of 12-well plates and treated with the indicated agents for 72 h or 6 days. The medium was replaced every other day during the course of the experiment with drug-containing medium (days 2 and 4). Cells were then washed with PBS, fixed and stained with SA-ß gal solution overnight. The cells were then photographed using the Evos XL Core cell imaging system (ThermoFischer, Waltham, MA) and senescent cells were quantified by counting 100 cells in three different fields for each replicate. A minimum of three technical and three biological replicates were performed for each condition.

For *in vivo* studies, mouse tumours tissues were snap-frozen in liquid nitrogen, embedded in OCT and cut into thin sections (5 μm). Senescence was measured by the SA-ß gal detection kit (Biovision, Milpitas, CA) by following the manufacturer’s protocol. Briefly, slides were washed with PBS and incubated in staining solution (prepared according to protocol) overnight. Slides were then washed with PBS and fixed in 70% glycerol. Images of the tissue sections were obtained by a Leica DM light microscope using the × 20 and × 40 optical lenses. Images were acquired with a SPOT Imaging Solutions camera and SPOT Advanced software.

For quantitation of cellular granularity, cells were plated (1 × 10^5^ cells per plate) on 10-cm plates and treated for 6 days; some of the cells were allowed to recover for 4 days without drug. Following treatment, cells were collected, stained with propidium iodide (1 mg ml^−1^) and analysed on the Beckman Coulter Gallios Flow Cytometer. The data were then analysed with the FlowJo software to obtain a distribution curve of side-scatter versus normalized cell counts.

### Annexin V and caspase-3 apoptosis assays

Apoptotic cells were measured by using the Alexa Fluor 488 Annexin V Dead Cell Apoptosis kit (Invitrogen, Waltham, MA) according to the manufacturer’s protocol. Briefly, cells were plated (1 × 10^5^ cells per plate) on 10-cm plates and treated with the indicated agents for 6 days and allowed to recover for 4 days. Cells were collected at the end of treatment (6 days) or after treatment+recovery, washed with 1 × Annexin binding buffer and stained with a solution of Alexa Fluor 488 Annexin V and propidium iodide (100 μg ml^−1^) as directed by the protocol. Samples were then analysed on the Beckman Coulter Gallios Flow Cytometer, and data were analysed with Kaluza software to obtained the percentages of Annexin V-positive/propidium iodide-negative (early apoptosis) and Annexin V-positive/propidium iodide-positive (late apoptosis) cells.

For the caspase-3 activity assay, following drug treatment, cells were collected, washed with a solution of PBS with 2% FBS and fixed with 100 μl of the fixation solution from the Cytofix/CytoPerm kit (BD Biosciences). Cells were then washed with Perm/Wash buffer and stained with phycoerythrin-conjugated rabbit active caspase-3 antibody (BD Biosciences) for 30 min on ice. Cells were finally washed, resuspended in the PBS with 2% FBS solution and analysed on the Becton Dickinson FACS Calibur Flow Cytometer to obtain the percentage of caspase-3-positive cells.

### Cellular ROS measurement

Cellular ROS levels were measured by using the CellROX Deep Red Flow Cytometry assay kit (ThermoFischer Scientific, Waltham, MA) according to the manufacturer’s protocol. Briefly, cells were plated (1 × 10^5^ cells per dish) on 10-cm dishes and treated for 6 days. Cells were then collected and stained with 500 nM CellROX reagent for 1 h at 37 °C. Samples were analysed on the Beckman Coulter Gallios Flow Cytometer using the 635-nm laser; data were analysed with the FlowJo software and mean fluorescence intensity (MFI) was obtained.

### Monodansylcadavarine measurement

Cells were seeded at a density of 1 × 10^5^ cells on 10-cm plates and treated for 6 days with various concentrations of palbociclib. At the end of drug treatment, cells were incubated with 50 μM MDC (Sigma-Aldrich) at 37 °C for 45 min. Cells were then collected, washed with PBS and suspended in a solution of PBS with 1% FBS. Samples were analysed on the Becton Dickinson FACS LSR II Flow Cytometer using the 355-nm ultraviolet laser. Data were analysed with the FlowJo software, and percentages of MDC-positive cells and MFI were obtained.

### Immunofluorescence

For the GFP-LC3 puncta assay, MCF7, MDA-MB-231 and MDA-MB-468 cells stably expressing GFP-LC3 were plated in six-well plates and treated with drug for 48 h. Following treatment, cells were washed with 1 × PBS, fixed with 4% paraformaldehyde for 10 min and mounted with Vectashield mounting media with DAPI. Cells were then visualized with Zeiss Confocal microscope LSM880 usign the 488 nm laser (GFP) for the presence of GFP-LC3 puncta. The puncta was quantified using ImgeJ.

For RFP-GFP-LC3 dual reporter assay, cells were transfected with ptf-LC3 vector and analysed as described previously^24^. Briefly, ptfLC3 vector was tranfected using Lipofectamine 2000 as per manufacturer’s instructions. Cells were then treated with the drug for 48 h, washed with 1 × PBS, fixed briefly (for 10 min) with 4% paraformaldehyde and mounted with Vectashield moutning media. They were then visualized using with Zeiss Confocal microscope LSM880 using the 488 and 643 nm channels to image the GFP+ve and RFP+ve LC3 puncta respectively. The puncta was quantified using ImgeJ to obtained the number of autophagosomes (yellow −RFP+ GFP+) and autophagolysosomes (red −RFP+).

### Transmission electron microscopy

For cell lines, ∼5,000 cells were plated in each well of 12-well plates and treated with the drug for 6 days. For xenografts, tumours were harvested and samples were cut into 1-mm^3^ pieces. The cell lines and tumour samples were processed similarly and Electron microscopy was performed at the High Resolution electron microscopy facility at MD Anderson Cancer Center as described previously^65^. Briefly, they were fixed with a solution containing 3% glutaraldehyde plus 2% paraformaldehyde in 0.1 M cacodylate buffer, pH 7.3. Samples were then washed in 0.1 M sodium cacodylate buffer and treated with 0.1% Millipore-filtered cacodylate-buffered tannic acid. They were fixed with 1% buffered osmium tetroxide for 30 min and stained *en bloc* with 1% Millipore-filtered uranyl acetate. The samples were dehydrated in increasing concentrations of ethanol, filtrated and embedded in LX-112 medium. They were then polymerized in a 60 °C oven for approximately 3 days. Ultrathin sections were cut in a Leica Ultracut microtome (Leica, Deerfield, IL), stained with uranyl acetate and lead citrate in a Leica EM Stainer, and examined in a JEM 1010 TEM (JEOL, USA, Inc., Peabody, MA) at an accelerating voltage of 80 kV. Digital images were obtained at magnifications of × 5,000, × 25,000 and × 50,000 using the AMT Imaging System (Advanced Microscopy Techniques Corp, Danvers, MA).

### *In vivo* xenograft studies

For all xenograft experiments, estrogen pellets (0.72 mg 17-beta estradiol pellet, 90-day release, Innovative Research of America, Sarasota, FL) were implanted subcutaneously into 4- to 6-week-old female nude mice. MCF7-T cells (5 × 10^6^ in a 1:1 ratio with matrigel (BD Biosciences)) were injected into the fifth and tenth inguinal mammary fat pads bilaterally. For the dose-determining experiment, once the tumours reached an average volume of 200 mm^3^, the tumour-bearing mice were randomized into five groups (*n*=3 per group) and treated with vehicle (0.5% methylcellulose) or 25 mg kg^−1^, 50 mg kg^−1^, 75 mg kg^−1^ or 150 mg kg^−1^ palbociclib. Palbociclib was administered daily via oral gavage for 7 consecutive days. For the combination treatment experiment, once the tumours reached an average volume of 250 mm^3^, the tumour-bearing mice were randomized into four groups (*n*=9 per group) and treated with vehicle (0.5% methylcellulose and PBS), HCQ (60 mg kg^−1^), palbociclib (25 mg kg^−1^) or a combination of palbociclib (25 mg kg^−1^) and HCQ (60 mg kg^−1^). Drugs were administered daily via oral gavage (palbociclib) or intraperitoneally (HCQ) for 21 days.

For Lys-05 toxicity experiment, non-tumour-bearing female nude mice were treated with varying concentrations of Lys-05 (1 mg kg^−1^, 5 mg kg^−1^, 10 mg kg^−1^ and 20 mg kg^−1^) everyday for 21 days via I.P. For the Lys-05 combination treatment experiment, once the orthotopic xenograft tumours reached an average volume of 250 mm^3^, the tumour-bearing mice were randomized into four groups (*n*=5 per group) and treated with vehicle (0.5% methylcellulose and PBS), Lys-05 (10 mg kg^−1^), palbociclib (25 mg kg^−1^) or a combination of palbociclib (25 mg kg^−1^) and Lys-05 (10 mg kg^−1^). Drugs were administered daily via oral gavage (palbociclib) or intraperitoneally (Lys-05) for 21 days.

For all xenograft studies, tumour volumes ((*L* × *W*^2^)*/*2)) were measured twice per week with calipers and mouse weight was measured every day. At the end of the treatment and recovery periods, mice were euthanized and the tumours were collected for further analysis. At the time the mice were euthanized, blood (0.2 ml) was collected by cardiac puncture through the left ventricle. Blood samples were subjected to complete blood count analysis (white blood cells, platelets and red blood cells) by the Siemens Adiva 120 Hematology System (Erlangen, Germany). Nude mice for all experiments were obtained from the Department of Experimental Radiation Oncology at The University of Texas MD Anderson Cancer Center, and mice received care in accordance with the Animal Welfare Act and the institutional guidelines of MD Anderson Cancer Center. The protocol for this study was approved by the Institutional Animal Care and Use Committee (IACUC) at The University of Texas MD Anderson Cancer Center (Houston, TX).

### Patient-derived xenograft studies

Breast tumour samples were obtained during routine surgery after informed consent was obtained under protocols approved by the MD Anderson Institutional Review Board. PDX models were developed as previously described^66^. Briefly, fresh primary tumours were collected using sterile technique, and ∼3-mm^3^ fragments of the tissue were transplanted to the fat pad of the fourth pair of mammary glands (both sides) in immunodeficient (severe combined immunodeficient) mice within 1 h of surgical resection. When the primary tumour outgrowths reached 10 mm in diameter, 3-mm^3^ fragments of the outgrowths were explanted to new hosts (*n*=3 per tumour) as secondary passage. As the tumour tissues can stably grow after two passages with our protocol, we considered the PDX line to have been successfully established at that point. The histology of the patient tumours of origin and the corresponding PDX lines were compared by hematoxylin and eosin staining and had very similar histology (data not shown). For the drug treatment experiment, 3-mm^3^ fragments of the PDX line were transplanted into the fat pat of the fourth mammary gland of female nude mice. Once the tumours reached an average volume of 200 mm^3^, mice were randomized into four groups (*n*=4 per group) and treated with vehicle (0.5% methylcellulose and PBS), HCQ (60 mg kg^−1^), palbociclib (25 mg kg^−1^) or combination of palbociclib (25 mg kg^−1^) and HCQ (60 mg kg^−1^). Drugs were administered daily via oral gavage (palbociclib) or intraperitoneally (HCQ) for 21 days. tumour volumes ((*L* × *W*^2^)/2)) were measured twice per week with calipers, and mouse weight was measured every day.

At the end of the treatment period, mice were killed and their tumours were collected for further analysis. All mice were obtained from the Department of Experimental Radiation Oncology at The University of Texas MD Anderson Cancer Center and received care in accordance with the Animal Welfare Act and the institutional guidelines of MD Anderson Cancer Center. The protocol for this study was approved by the IACUC at MD Anderson Cancer Center.

### Immunohistochemistry analysis of mouse tumour tissues

For BrdU assessment, mice were administered BrdU solution (5 mg kg^−1^ intraperitoneally) 2 h before killing. After death, the tumour was resected and snap-frozen in liquid nitrogen, embedded in OCT, cut and utilized for measurement of senescence by SA-ß gal staining. The remaining tissue was fixed in formalin and used for assessment of histology and of proliferation by BrdU as described previously^63^. Briefly, 5-μm sections from the formalin-fixed, paraffin-embedded tumour tissues were stained with standard hematoxylin and eosin. Other sections of the tumour blocks were subjected to immunohistochemical (IHC) staining. After paraffin removal, tumour sections were heated in a water bath for 20 min in 10 mM sodium citrate buffer (pH 6.0) at 90 °C to retrieve nuclear antigens. Endogenous peroxidase activity was quenched with a 3% hydrogen peroxide solution. Sections were blocked with 1.5% normal goat serum and incubated overnight at 4 °C with rat monoclonal antibody to BrdU (clone BU1/75 [ICR1]; GeneTex Inc, San Antonio, TX) diluted at 1:500. Slides were developed using the VECTASTAIN Elite ABC kit (PK4004; Vector Laboratories, Burlingame, CA), followed by staining with DAB substrate (Vector Laboratories) and counterstaining with hematoxylin (DAKO), and then were mounted. Staining was evaluated with a Leica DM light microscope using the × 40 optical lenses. Images were acquired on a SPOT Imaging Solutions camera with SPOT Advanced software. BrdU was quantified as percentage of BrdU-positive cells, which was calculated as percentage of BrdU-positive nuclei from a total of 300 tumour cells from three fields of view.

For 4HNE and Anti-8OHdG immunostaining; after paraffin removal, heat-induced antigen retrieval was performed for 10 min in 10 mM sodium citrate buffer (pH 6.0) at 98 °C. Endogenous peroxidase activity was blocked with 3% hydrogen peroxidase for 15 min. Slides were then incubated for 1 h with diluted rabbit blocking serum. The sections were incubated for overnight at 4 °C with Anti-8OHdG mouse monoclonal antibody (clone N45.1, Genox, Baltimore, MD, 1:100 dilution) and Anti-4-HNE mouse monoclonal antibody (clone HNEJ-2, Genox, Baltimore, MD, 1:50 dilution). The slides were incubated for 30 min with diluted biotinylated secondary antibody and 30 min with Vectastain Elite ABC kit (Vector Laboratories, United States). For the evaluation of 4HNE and Anti-8OHdG antibody expression, slides were scored separately for percentage and intensity of the cells. Percentage positivity was graded using 0–4 scale, where 0 represented no stained cells, 1 was 1 to 5% stained cells, 2 was 6 to 30% stained cells, 3 was 31 to 70% stained cells and 4 was 71 to 100% stained cells. Staining intensity was scored as follows: 0, no staining; 1, weak positive; 2, intermediate positive; and 3, strong positive. *H* score calculated by multiplying the percentage of positive cells and intensity of staining.

### Western blot analysis

Western blot analysis was performed as described previously^63^. Briefly, cells were seeded at a density of 1 × 10^5^ cells on 10-cm plates and treated for 6 days with the indicated drugs. Following treatment, cells were collected and subjected to lysis with RIPA buffer (150 mM NaCl, 10 mM Tris, pH 7.3, 0.1% sodium dodecyl sulfate (SDS), 1% Triton X-100, 1% deoxycholate and 5 mM ethylene-diaminetetraacetic acid) containing protease inhibitors. For mouse tissues, the tumours were minced into small pieces on dry ice and immersed in RIPA buffer for lysis. Lysates were then subjected to centrifugation at 45,000 r.p.m. for 45 min at 4 °C to obtain the protein lysates in the supernatant. Protein concentration was determined by Bradford Protein Assay dye (Bio-Rad), and 50 μg of protein per sample was resolved by SDS–polyacrylamide gel electrophoresis as described previously. Blots were blocked with Blotto milk for 1 h at room temperature and incubated with primary antibody overnight at 4 °C. They were then incubated with goat anti-rabbit or goat anti-mouse immunoglobulin–horseradish peroxidase conjugates (Pierce, Rockford, IL) at a dilution of 1:5,000 in Blotto for 1 h. Blots were then washed and developed using a Renaissance chemiluminescence system (Perkin Elmer Life Sciences, Inc.) by following the manufacturer’s instructions. The developed and scanned blots were then analysed by the ImageJ software to obtain densitometry values.

### RPPA and data analysis

The mouse tumour tissues were dissected on dry ice and subjected to lysis in RIPA buffer (1% Triton X-100, 50 mM HEPES, pH 7.4, 150 mM NaCl, 1.5 mM MgCl_2_, 1 mM EGTA, 100 mM NaF, 10 mM Na pyrophosphate, 1 mM Na_3_VO_4_, 10% glycerol, and freshly added protease and phosphatase inhibitors; Roche Applied Science, Indianapolis, IN). Tumour lysates were then subjected to centrifugation and protein concentration was determined by using the Bradford reagent. Protein concentration was adjusted to 1.5 μg μl^−1^ and mixed with 4 × SDS Sample Buffer containing 40% glycerol, 8% SDS, 0.25 M Tris-HCL and 2-mercapto-ethanol at pH 6.8. Samples were boiled for 5 min, and the RPPA analysis was performed by the Functional Proteomics core facility at MD Anderson Cancer Center. The slide images were quantified using MicroVigene 4.0 (Vigene-Tech, Carlisle, MA). The spot level raw data was processed with the R package SuperCurve (https://r-forge.r-project.org/ projects/supercurve), which returns the estimated protein concentration (raw concentration) and a quality control score for each slide. The raw concentration data was then normalized by median-centring for each sample across all the proteins, to correct for loading bias.

For RPPA data analysis, one-way (analysis of variance) ANOVA was used to identify proteins that are differentially expressed between treatment groups. To adjust for multiple comparisons, Benjamini–Hochberg procedure was used to estimate false discovery rate (FDR). Tukey HSD tests were used for *post hoc* pairwise comparisons. To compare the different Palbociclib doses treatments *in vivo* (Vehicle, 25 mg kg^−1^, 75 mg kg^−1^ and 150 mg kg^−1^), proteins with 20% FDR in ANOVA, Tukey *P*<0.05 and fold change > ±1.2 was identified as significantly differentially expressed between groups. A heat map was then drawn using significant proteins from the ANOVA analysis, and ordered based on KEGG cell cycle, senescence and autophagy/catabolism pathways. Pearson distance metric and Ward's minimum variance was used to cluster the samples in the heat map. For comparing the combination treatments (Vehicle, HCQ, Palbociclib and Palbociclib + HCQ) *in vivo*, proteins with 15% FDR in ANOVA, Tukey *P*<0.05 and fold change > ±1.2 were identified as significantly differentially expressed in pairwise analysis. Pathway score for cell cycle and senescence pathways was calculated using mean expression level (in log_2_ scale) of the selected proteins from ANOVA analysis.

### Bioinformatics and TCGA analysis

Alterations in CDK4, CDK6, CCND1, Rb1 and CCNE1 were obtained using cBio Portal^67^ from the TCGA RNA seq data for breast, ovarian, lung, pancreatic, colon and prostate cancer. For biomarker analysis, gene expression data for the 23 cell lines under study was obtained from Kao *et al*.^68^, and Gene Set Enrichment Analysis (GSEA) was performed against the BioCarta gene sets^69^ to obtain a heat map of the cell cycle genes.

### Breast cancer patient samples, clinical data and statistical analysis

The Department of Medical Oncology at MD Anderson maintains a prospective curated database of patients from 1997 onward. This database was searched for all patients with breast cancer who had received palbociclib, and this search was supplemented with a manual search. Key demographic, clinical and pathologic data, cancer treatment details and response to therapy were abstracted into a working database for analysis under an IRB-approved protocol. Outcomes of interest that were recorded included response, stable disease and progression (based on the physician’s determination, but not always based on RECIST criteria) and associated durations of disease response and stability. To demonstrate the applicability of our staining procedure, blocks with the largest available tumour specimen was chosen for each patient (obtained after informed consent) and retrieved from the archives of the Department of Pathology at MD Anderson under an IRB-approved protocol; this included specimens from the primary tumour and/or local and/or metastatic recurrences. Our working database includes results of pathology reports, including tumour histology and grade and standard IHC analyses for ER and progesterone receptors for primary breast cancer biopsies and surgical specimens as well as local and distant recurrences that were subject to biopsy or surgical excision.

Patient, tumour and treatment characteristics were evaluated and compared between patients who were or were not free from progression; 6-month and 12-month progression rates were calculated for each of factor using the Kaplan–Meier method, and differences were examined using the log-rank test (Supplementary Tables 1 and 2). Univariable and multivariable Cox model analyses were used to determine the influence of patient, tumour and treatment factors of known or potential prognostic value on PFS (Supplementary Tables 3 and 4). All factors with *P*≤0.1 in the univariable analyses were entered into a full model, and the final model was selected by using a backwards elimination procedure. Model performance was quantified using Harrell’s concordance index^70^. The discriminative ability of the model was assessed using the C-index for comparative purposes with the literature, as well as the concordance probability estimate due to the high degree of censoring in the data^70^. The C-index can range from perfect concordance (1.0) to random predictions (0.5). Similar to the area under the receiver operating characteristic curve, concordance probability estimate can range from perfect concordance (1.0) to perfect discordance (0.0). In addition, Akaike’s information criterion (AIC) was calculated. The AIC takes into account how well the model fits the data as well as the complexity of a model, thereby reducing the risk of overfitting. After comparisons, the final model with the lowest AIC value and the highest C-index was reported. All statistical analyses were performed using R 3.3.2 (http://www.r-project.org/). All *P* values were two-tailed, and *P*≤0.05 was considered significant, and adjustments for multiple factors were not made.

### Immunohistochemical staining of patient samples

For the 109 breast cancer samples recovered, two serial 5-μm sections were cut and mounted on Superfrost Plus glass slides. The antigen retrieval and washing steps were performed as described for BrdU immunohistochemistry analysis. For cyclin E and Rb IHC, two commercially available primary antibodies were used: rabbit polyclonal antibody to cyclin E (clone C19; Santa Cruz Biotechnology, Santa Cruz, CA) diluted 1:1,000 and Rb mouse monoclonal antibody (Clone 4H1; Cell Signaling Technology, Denvers, MA) diluted 1:100. Slides were developed using the VECTASTAIN Elite ABC kit (PK6101 and PK6102; Vector Laboratories, Burlingame, CA) followed by staining with DAB substrate (Vector) and counterstaining with hematoxylin (DAKO), and then were mounted. Tumour cell blocks known to express high levels of LMWE and Rb were included in each batch as positive controls, and negative controls were prepared by replacing the primary antibody with PBS buffer. Staining was evaluated with a Leica DM light microscope using the × 20 and × 40 optical lenses. Images were acquired by a SPOT Imaging Solutions camera and SPOT Advanced software. Pathologists at MD Anderson (C.K. and P.O.S.) evaluated and scored Rb and LMWE staining.

### Immunohistochemical scoring of Rb and cyclin E

Cyclin E staining was scored by two pathologists blinded to patient outcomes (data not shown). Scores (0=negative, 1=weak staining, 2=moderate staining and 3=strong staining) were assigned for nuclear and cytoplasmic staining according to percentage of cells stained and intensity of staining, as described previously^41^. Each tumour sample was scored separately for nuclear and cytoplasmic cyclin E expression, and LMWE status was assigned as follows: LMWE negative was defined as no staining or nuclear staining only; LMWE positive was defined as nuclear + cytoplasmic staining or cytoplasmic staining only.

For Rb staining, the intensity of staining and percentage of positive cells were evaluated separately. Staining intensity was scored as follows: 0, no staining; 1, weak positive (faint yellow staining); 2, intermediate positive; and 3, strong positive (brown staining). The number of positive cells was visually evaluated and stratified as follows: <1%, 0 (negative); 1 to <5% positive cells, 1 (weak); 5–50% positive cells, 2 (moderate); >50% positive cells, 3 (strong). The sum of the staining intensity and percentage of positive cell scores was used to determine the staining index for each section, with a minimum score of 0 and maximum score of 6; scores >1 were defined as Rb positivity. Using this cut-off, we compared Rb-positive to Rb-negative tumours.

IHC analysis for Rb and cyclin E for the NCI TMA tumour samples were performed and scored as described above for the Palbociclib-treated patient samples.

### Statistical analysis

All experiments were performed with a minimum of three technical and three biological replicates, and values reported are the mean of the three biological replicates, unless otherwise indicated. Error bars represent the s.d. from the mean, unless otherwise indicated. Pairwise comparisons were analysed using multiple *t*-tests (one unpaired *t*-test per row), with corrections applied (Holm–Sidak method) for multiple comparisons. When comparing data from experiments with multiple groups, a regular one-way ANOVA (no matching) was used with the Benjamini–Hochberg procedure to adjust for multiple comparisons. Tukey HSD tests were used for *post hoc* analysis. Kaplan–Meier survival analysis was performed by using the log-rank (Mantel–Cox) test. For all tests, differences were considered statistically significant at a *P* value of 0.05 or less. For all figures, NS: *P*>0.05; *P<0.05; **P<0.01; ***P<0.001; ****P<0.0001. All statistical analyses were performed using the GraphPad Prism software and R.

### Data availability

All data are available within the Article and Supplementary Files, or available from the authors upon request.

## Additional information

**How to cite this article:** Vijayaraghavan, S. *et al*. CDK4/6 and autophagy inhibitors synergistically induce senescence in Rb positive cytoplasmic cyclin E negative cancers. *Nat. Commun.* **8**, 15916 doi: 10.1038/ncomms15916 (2017).

**Publisher’s note:** Springer Nature remains neutral with regard to jurisdictional claims in published maps and institutional affiliations.

## Supplementary Material

Supplementary Information
