# Supplementary material for: CDK4/6 and autophagy inhibitors synergistically induce senescence in Rb positive cytoplasmic cyclin E negative cancers
Source: Nat Commun. 2017 Jun 27;8:15916. doi: 10.1038/ncomms15916 (PMC5490269; doi:10.1038/ncomms15916)
Supplement: Supplementary Information [file ncomms15916-s1.pdf]

Type of file: PDF
Size of file: 0 KB
Title of file for HTML: Supplementary Information
Description: Supplementary Figures and Supplementary Tables

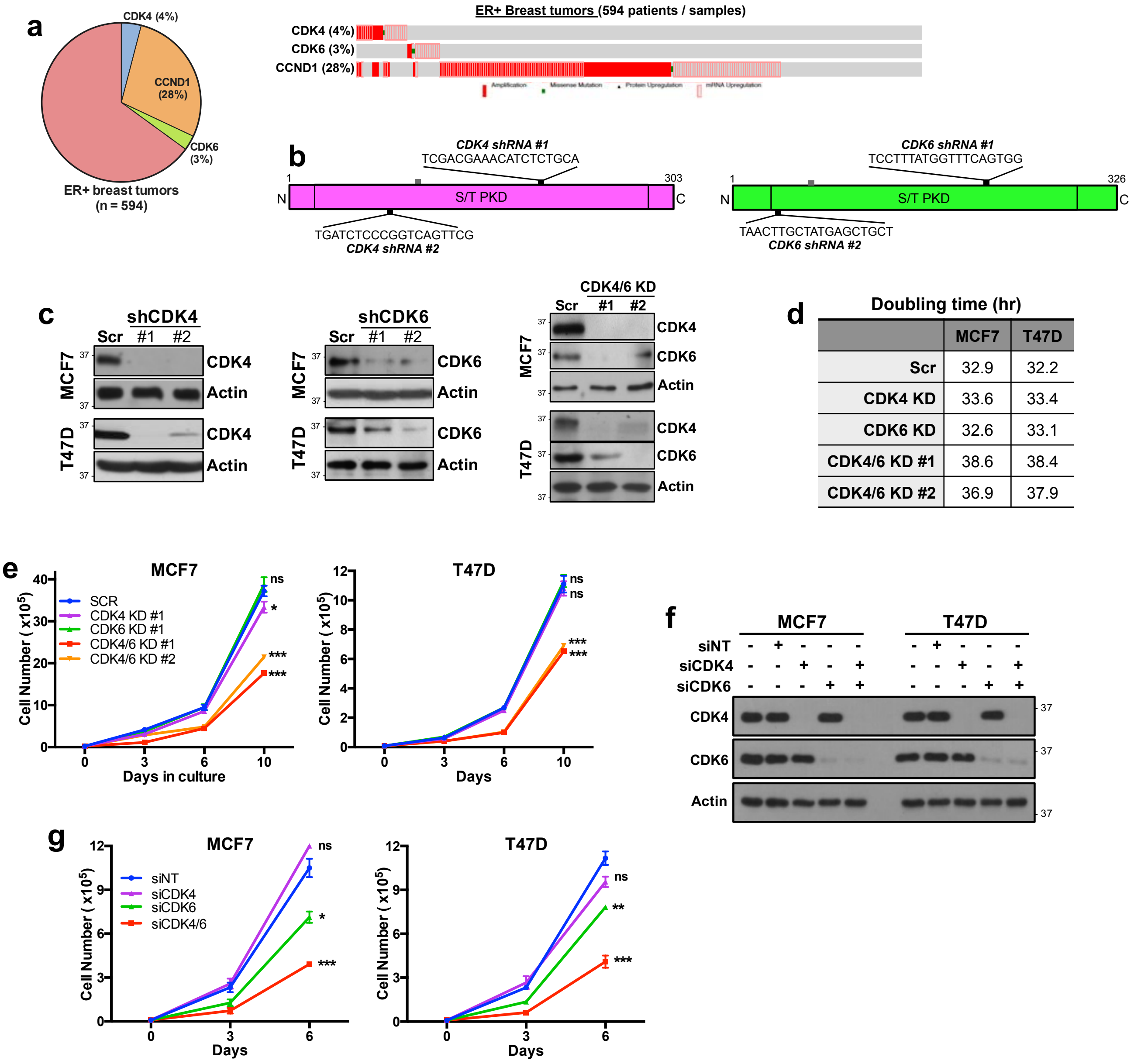

	MCF7	T47D
Scr	32.9	32.2
CDK4 KD	33.6	33.4
CDK6 KD	32.6	33.1
CDK4/6 KD #1	38.6	38.4
CDK4/6 KD #2	36.9	37.9

Supplementary Figure 1. CDK4/6 downregulation has a growth inhibitory effect on ER+ve breast cancer cells: a) Alterations in the CDK4/CDK6/cyclin D pathway among 594 patients with ER+ breast cancer from the TCGA database. **b)** Schematic depicting the location of the shRNA sequences (#1 and 2) on the *CDK4* and *CDK6* genes. **c)** Western blot showing levels of CDK4 and CDK6 proteins in MCF7 and T47D cells after transfection with shRNA for Scrambled (SCR) or CDK4 and CDK6, separately or combined. **d,e)** Impact of knocking down (KD) CDK4 and/or CDK6 on the doubling time (d) and proliferation (e) of MCF7 and T47D cells when compared to control Scrambled shRNA–transfected cells. **f)** Western blot showing levels of CDK4 and CDK6 proteins in MCF7 and T47D cells after transfection with ON-TARGETplus SMARTpool siRNA non-targeting (siNT) or CDK4 and CDK6, separately or combined **g)** Impact of siRNA knocking down of CDK4 and/or CDK6 on the proliferation (g) and doubling time (h) of MCF7 and T47D cells compared to NT siRNA transfected cells. All data represent mean±SD from three independent experiments; p-values were calculated in comparison to cells treated with SCR or siNT. ns: p>0.05; *p<0.05; **p<0.01; ***p<0.001; ****p<0.0001.

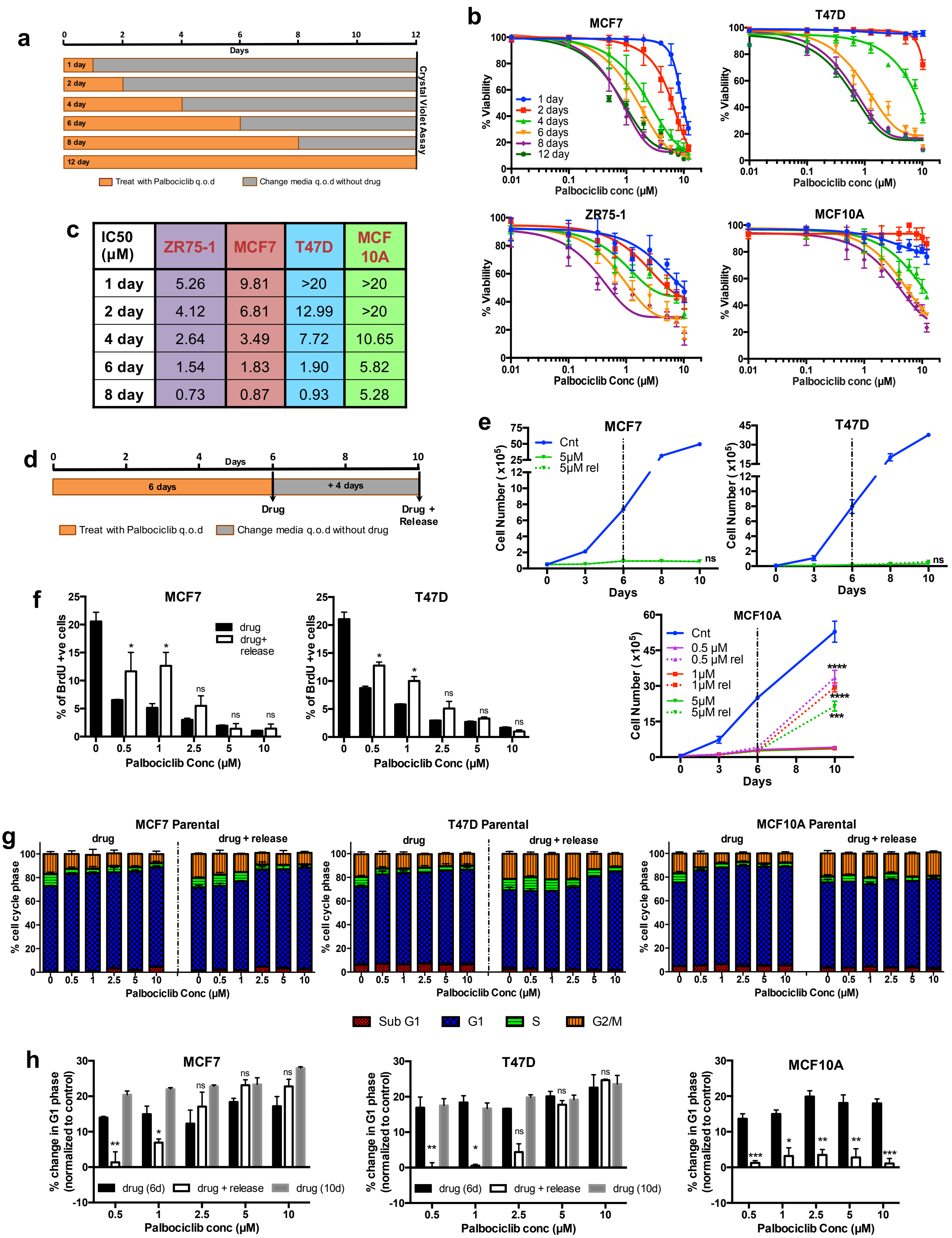

IC50 (μM)	ZR75-1	MCF7	T47D	MCF 10A
1 day	5.26	9.81	>20	>20
2 day	4.12	6.81	12.99	>20
4 day	2.64	3.49	7.72	10.65
6 day	1.54	1.83	1.90	5.82
8 day	0.73	0.87	0.93	5.28

Supplementary Figure 2. Palbociclib induces dose-dependent growth inhibition and G1 arrest in ER+ve breast cancer cells: a) Schematic depicting the palbociclib treatment schedule for the dose-response experiments shown in Figs 1a and Supplementary Fig. 2b. Drug-containing medium or drug-free medium was replaced every other day (q.o.d). **b)** Dose-response curves for MCF7, T47D, MCF10A, and ZR75-1 cells treated with DMSO or increasing concentrations (conc) of CDK4/6 inhibitor palbociclib (0.01 to 12 μM) for 1, 2, 4, 6, 8 or 12 days. **c)** Half-maximal inhibitory concentration (IC_{50}) values of MCF7, T47D, ZR75-1, and MCF10A cells as calculated from dose-response experiments in Figs 1a and Supplementary Fig. 2b. **d)** Schematic depicting the palbociclib treatment schedule where cells were treated for 6 days and cultured in drug-free medium for 4 days to examine reversibility. Medium was replaced every other day (q.o.d). **e-h)** MCF7, T47D, and MCF10A cells were treated with DMSO (Cnt) or varying concentrations of palbociclib for 6 days and some cells were allowed to recover for 4 days after treatment (release) to examine reversibility. Cells were then subjected to e) cell counting to assess growth inhibition of MCF10A; f) flow cytometry analysis for BrdU-positive cells, a measure of S phase progression; g) cell cycle analysis via flow cytometry; and h) cell cycle analysis to determine percentage change in the G1 phase. All data represent mean±SD from three independent experiments; p-values were calculated by comparing values at the end of 6 day drug treatment with those at the end of drug + release. ns: p>0.05; *p<0.05; **p<0.01; ***p<0.001; ****p<0.0001.

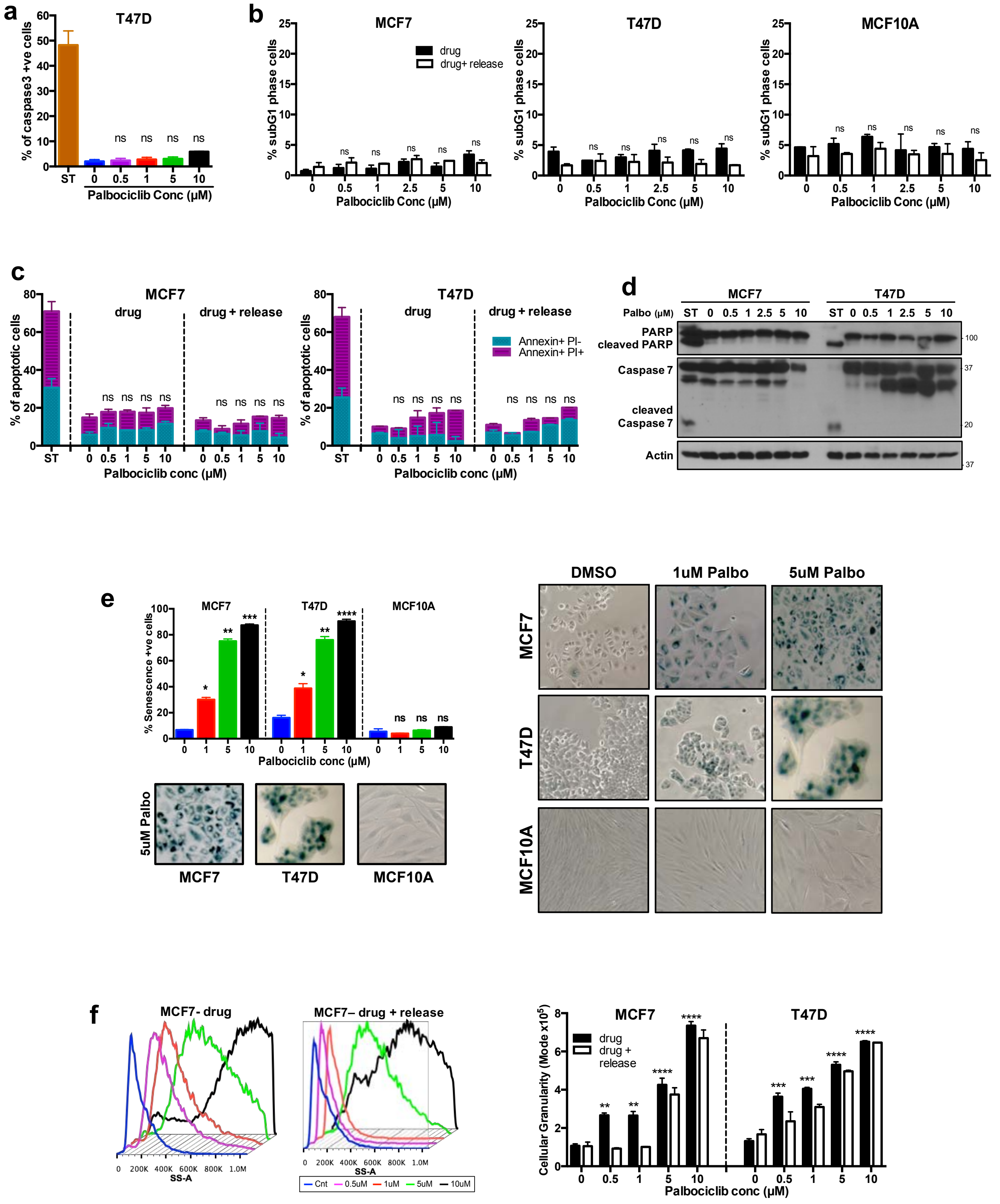

a
T47D
% of caspase3 +ve cells
ST
0
0.5
1
5
10
ns
Palbociclib Conc (µM)
b
MCF7
T47D
MCF10A
% subG1 phase cells
drug
drug+ release
ns
0
0.5
1
2.5
5
10
Palbociclib Conc (µM)
c
MCF7
T47D
drug
drug + release
% of apoptotic cells
Annexin+ PI-
Annexin+ PI+
ns
ST
Palbociclib conc (µM)
d
MCF7
T47D
Palbo (µM)
ST 0 0.5 1 2.5 5 10
PARP
cleaved PARP
Caspase 7
cleaved Caspase 7
Actin
100
37
20
37
e
MCF7
T47D
MCF10A
% Senescence +ve cells
*
**
***
****
ns
Palbociclib conc (µM)
5uM Palbo
DMSO
1uM Palbo
5uM Palbo
f
MCF7- drug
MCF7– drug + release
0 200K 400K 600K 800K 1.0M
SS-A
Cnt
0.5uM
1uM
5uM
10uM
Cellular Granularity (Mode x10^5)
drug
drug + release
MCF7
T47D
Palbociclib conc (µM)

Supplementary Figure 3 (contd.)

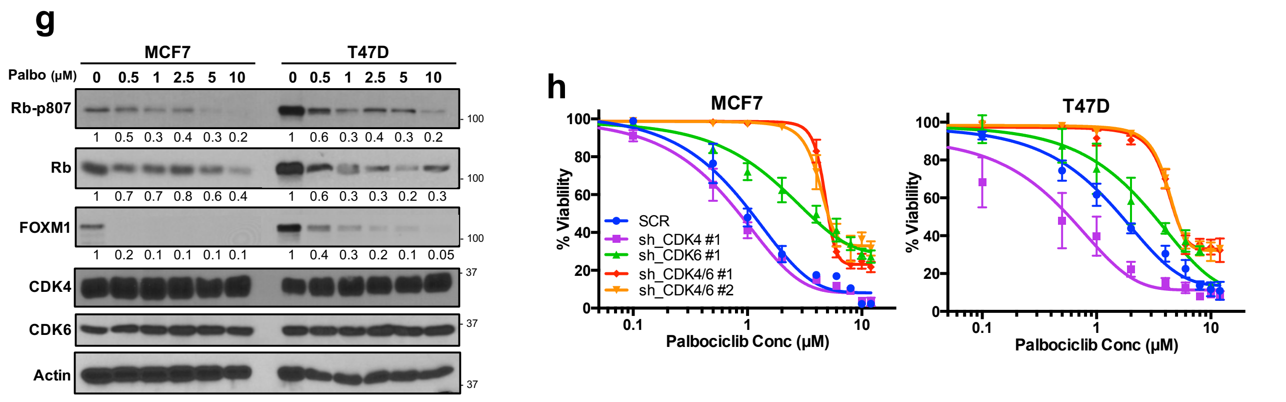

Supplementary Figure 3. Palbociclib did not trigger apoptosis but induced senescence in a dose-dependent manner: a) Percentage of caspase-3 positive cells determined by flow cytometry in T47D cells treated with DMSO, 3 μM staurosporine (positive control), or varying concentrations of palbociclib for 6 days. **b)** cell cycle analysis to determine percentage of cell in sub G1 phase, as a measure of apoptosis. **c)** Flow cytometry measurement of apoptotic cells (early apoptosis: Annexin V+/ PI-; late apoptosis: Annexin V+/PI+) in cells treated as in (a). **d)** Western blot analysis of apoptotic proteins in MCF7 and T47D treated with DMSO or palbociclib (Palbo) for 6 days: PARP, cleaved PARP, caspase-7, and cleaved caspase-7, with actin as loading control. **e)** Senescence-associated SA-ß gal staining with representative images inMCF7 and T47D cells upon treatment with DMSO or palbociclib for 6 days. **f)** Side scatter analysis and quantification to assess granularity of MCF7 and T47D cells treated as in (e). **g)** Western blot analysis of MCF7 and T47D cells treated with DMSO or palbociclib for levels of cell cycle proteins: Rb, p-Rb (S807), FOXM1, CDK4, and CDK6. Values show densitometry of the western blots as normalized to the loading control actin. **h)** Proliferation of MCF7 and T47D cells upon knockdown of CDK4 and/or CDK6 and treatment with DMSO or increasing concentrations of palbociclib (0.01 to 12 μM) for 6 days. All data represent mean±SD from three independent experiments; p-values were calculated in comparison to cells treated with DMSO (Control) unless indicated. ns: $p>0.05$; *$p<0.05$; **$p<0.01$; ***$p<0.001$; ****$p<0.0001$.

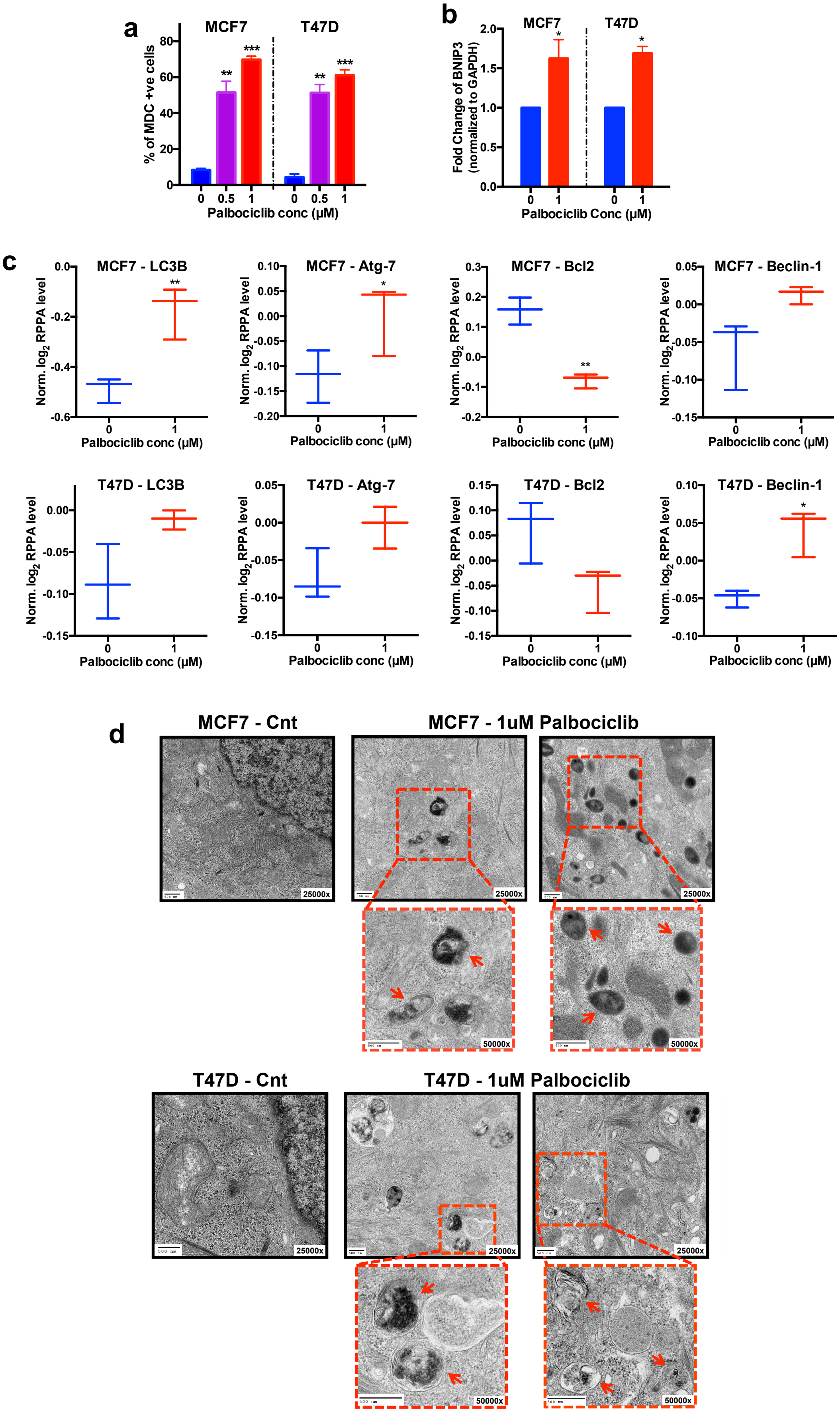
a
MCF7
T47D
% of MDC +ve cells
Palbociclib conc (μM)
b
MCF7
T47D
Fold Change of BNIP3 (normalized to GAPDH)
Palbociclib Conc (μM)
c
MCF7 - LC3B
MCF7 - Atg-7
MCF7 - Bcl2
MCF7 - Beclin-1
T47D - LC3B
T47D - Atg-7
T47D - Bcl2
T47D - Beclin-1
Norm. log2 RPPA level
Palbociclib conc (μM)
d
MCF7 - Cnt
MCF7 - 1uM Palbociclib
T47D - Cnt
T47D - 1uM Palbociclib
25000x
50000x

Supplementary Figure 4. CDK4/6 inhibition induces autophagy at low concentrations in ER positive breast cancer: a) Measurement of monodansylcadavarine (MDC)-positive acidic vesicles, including autophagosomes, by flow cytometry in MCF7 and T47D cells treated with varying concentrations of palbociclib for 6 days (b). **b)** mRNA level of BNIP3 (normalized to GAPDH) in MCF7 and T47D cells treated with 1 μM palbociclib. **c)** Expression (normalized log_2 level) of LC3B, Atg-7, BCl2 and Beclin-1 determined by RPPA analysis of MCF7 and T47D cell lines treated with palbociclib for 6 days. **d)** Representative TEM microphotographs of MCF7 and T47D cells treated with DMSO (Cnt) or palbociclib (Palbo; 1 μM or 5 μM) for 6 days. Red arrows indicate double-membraned autophagosomes. Scale bars equal 500 nm. All data represent mean±SD from three independent experiments; p-values were calculated in comparison to cells treated with DMSO (Control) or siNT. ns: p>0.05; *p<0.05; **p<0.01; ***p<0.001; ****p<0.0001.

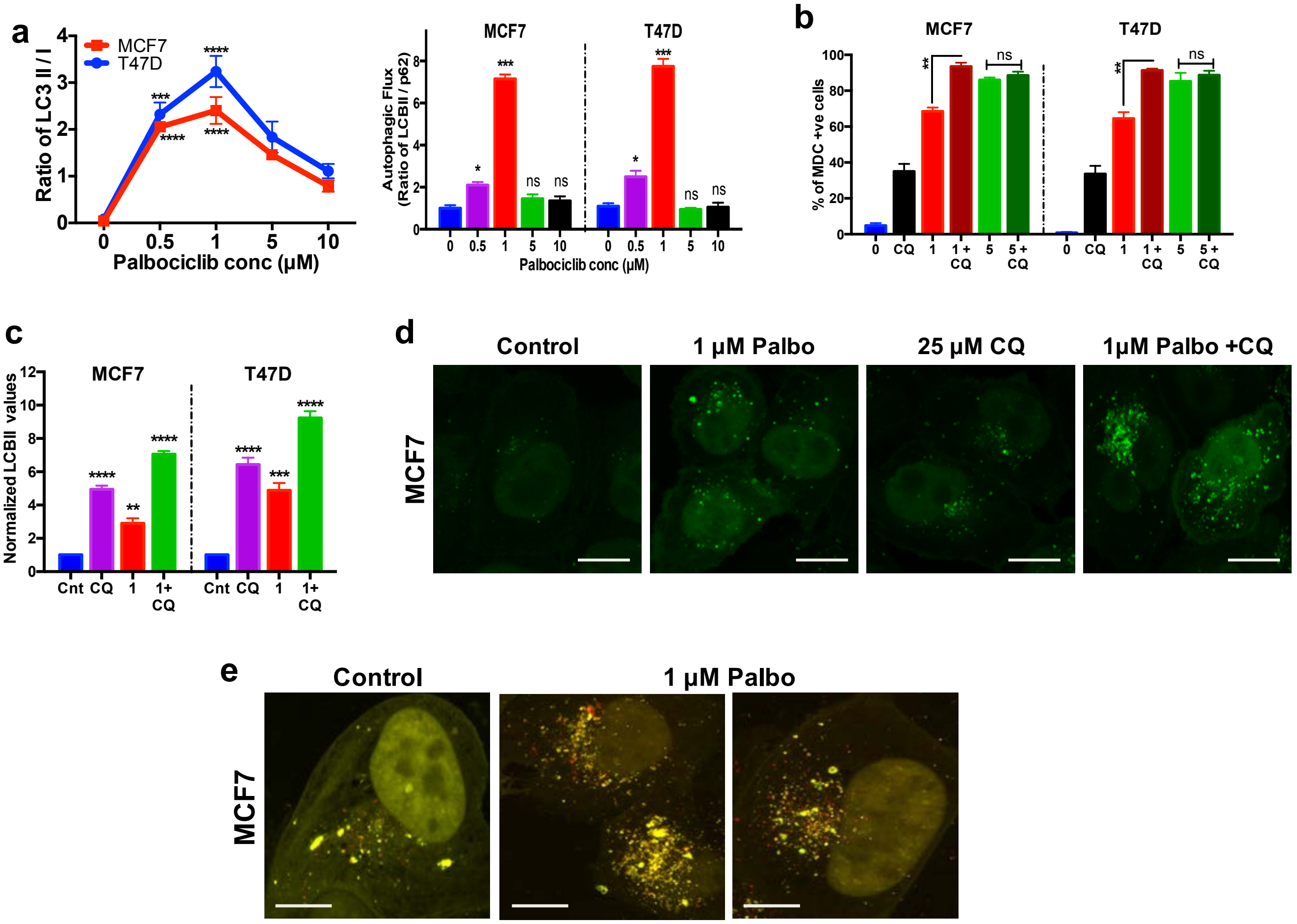

Supplementary Figure 5. Palbociclib induces autophagy with an intact flux at low concentrations in ER+ breast cancer: a) Autophagic flux, calculated as ratio of LC3B-II to LC3B-I and LC3B-II to p62 from densitometry values (normalized to corresponding levels of actin) of western blots from Fig. 1h. **b)** Measurement and quantification (Mean Fluorescence Intensity) of MDC-positive cells by flow cytometry in MCF7 and T47D cells treated with a combination of lysosomal blocker chloroquine (CQ; 10 μM) and DMSO or palbociclib (1 or 5 μM) for 6 days. **c)** Densitometry values for western blots in Fig. 1j to obtain LC3B-II protein levels (normalized to corresponding levels of actin). **d)** Representative confocal images of GFP-LC3 expressing MCF7 cells treated with 25 μM CQ (for 1 hour), 1 μM palbociclib or combination of palbociclib and CQ for 48 hours. Scale bars are 50 μm. **e)** Representative confocal images of RFP-GFP-LC3 expressing MCF7 cells treated with 1 μM palbociclib for 48 hours. Scale bars are 50 μm. All data represent mean±SD from three independent experiments; p-values were calculated in comparison to cells treated with DMSO (Control) unless indicated. ns: $p>0.05$; *$p<0.05$; **$p<0.01$; ***$p<0.001$; ****$p<0.0001$.

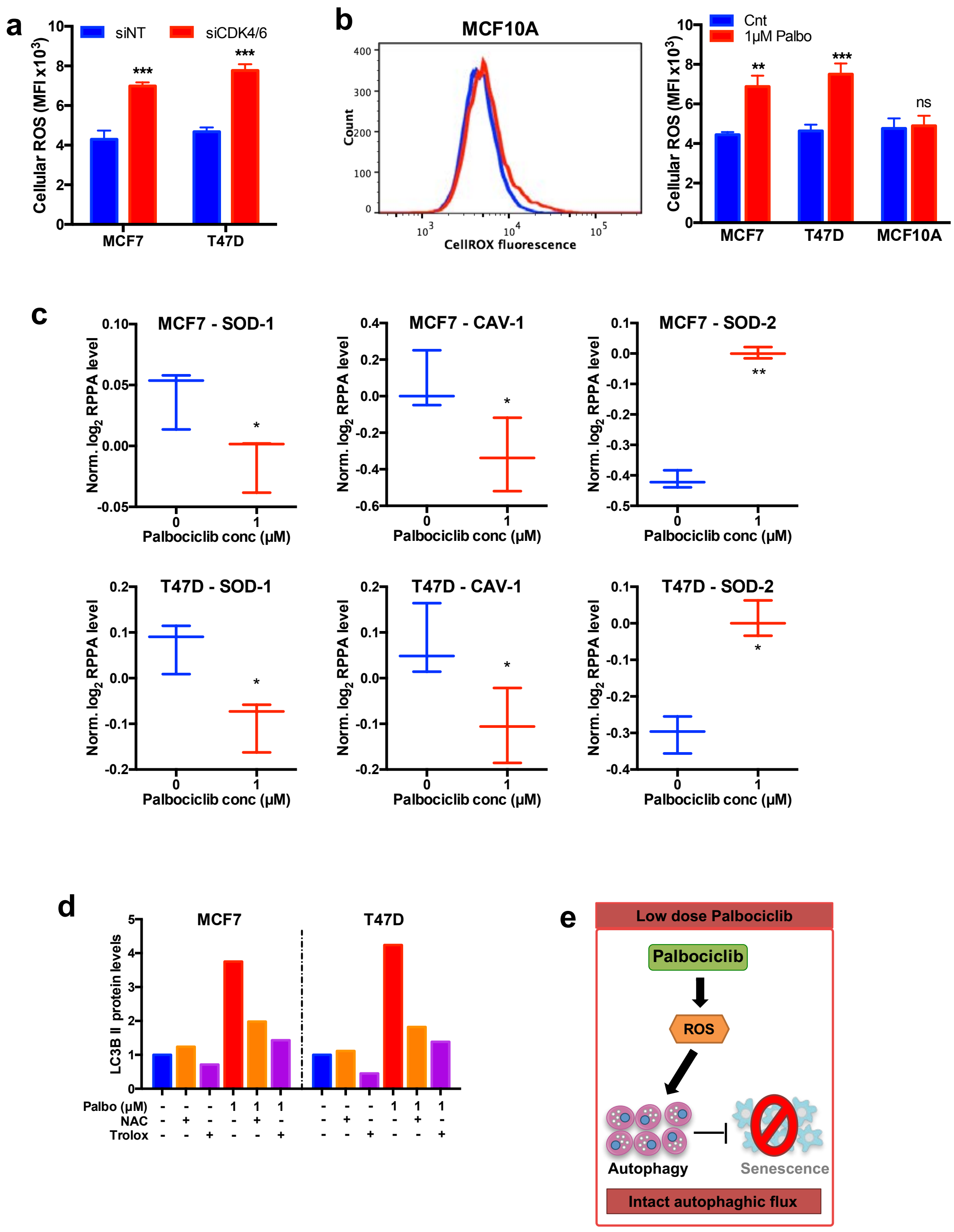

a
Cellular ROS (MFI x10^3)
siNT
siCDK4/6
***
***
MCF7
T47D
b
MCF10A
Count
CellROX fluorescence
Cnt
1μM Palbo
**
***
ns
MCF7
T47D
MCF10A
c
MCF7 - SOD-1
MCF7 - CAV-1
MCF7 - SOD-2
T47D - SOD-1
T47D - CAV-1
T47D - SOD-2
Norm. log$_2$ RPPA level
Palbociclib conc (μM)
d
MCF7
T47D
LC3B II protein levels
Palbo (μM)
NAC
Trolox
e
Low dose Palbociclib
Palbociclib
ROS
Autophagy
Senescence
Intact autophagic flux

Supplementary Figure 6. Low dose palbociclib induces ROS-mediated autophagy in ER+ breast cancer cells: a) Cellular reactive oxygen species (ROS) measurement and quantification (mean fluorescence intensity [MFI]) of ROS levels in MCF7 and T47D cells upon transfection with NT siRNA or siRNA against CDK4 and CDK6. **b)** Cellular reactive oxygen species (ROS) measurement and quantification (mean fluorescence intensity [MFI]) of reactive oxygen species (ROS) levels in MCF7, T47D and MCF10A cells upon treatment with DMSO or palbociclib for 6 days. **c)** Expression (normalized $\log_2$ level) of SOD-1, CAV-1 and SOD-2 determined by RPPA analysis of MCF7 and T47D cell lines treated with palbociclib for 6 days. **d)** LC3B II protein levels in MCF7 and T47D cells treated with combination of ROS scavenger (10 mM NAC or 0.1 mM trolox) with 1μM palbociclib for 6 days. **e)** Schematic showing the biphasic effect of palbociclib on ER+ breast cancer cells. All data represent mean±SD from three independent experiments; p-values were calculated in comparison to cells treated with DMSO (Control) or siNT unless indicated. ns: $p>0.05$; *$p<0.05$; **$p<0.01$; ***$p<0.001$; ****$p<0.0001$.

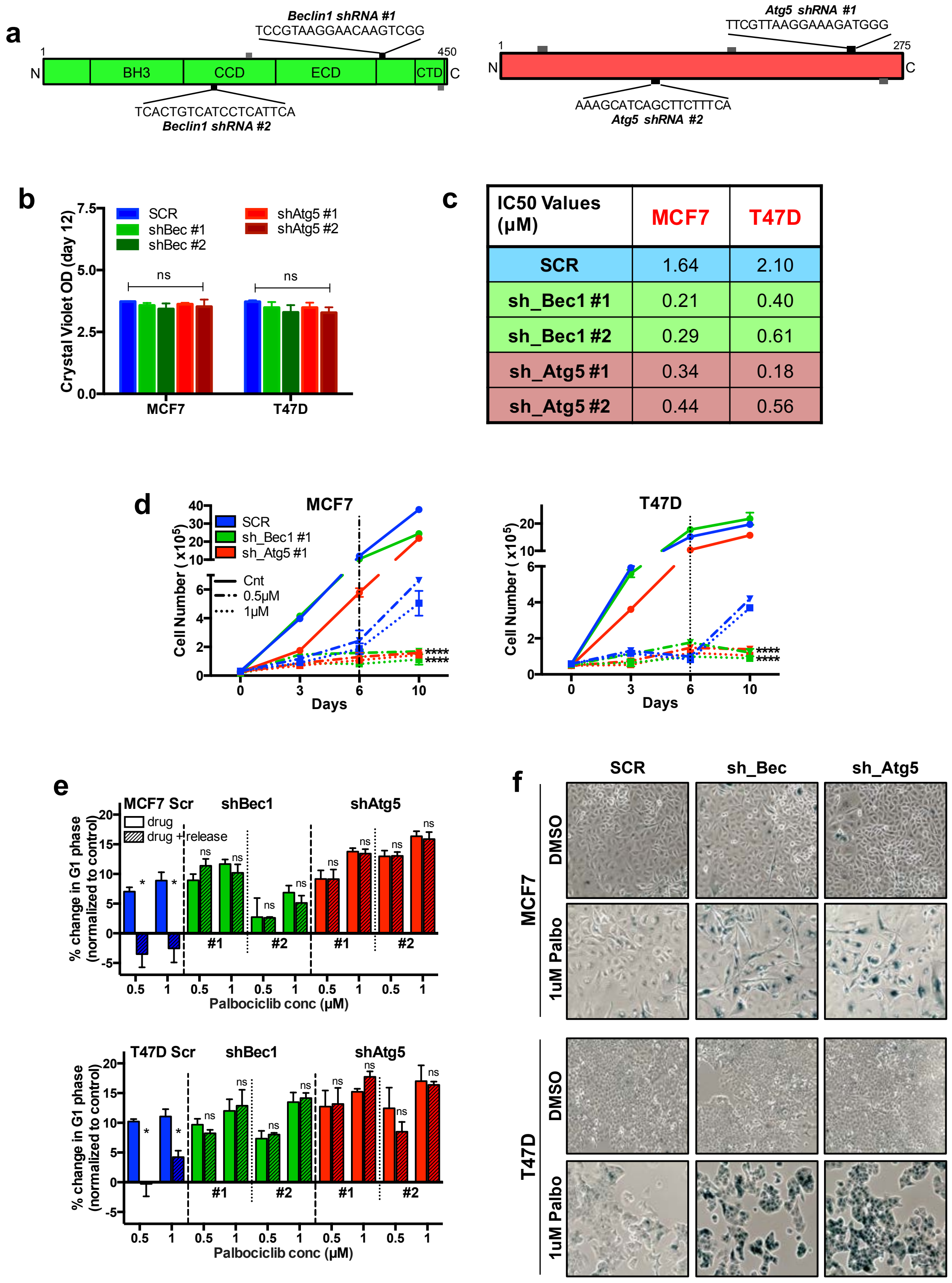

IC50 Values (μM)	MCF7	T47D
SCR	1.64	2.10
sh_Bec1 #1	0.21	0.40
sh_Bec1 #2	0.29	0.61
sh_Atg5 #1	0.34	0.18
sh_Atg5 #2	0.44	0.56

Supplementary Figure 7. Ablation of autophagic genes sensitizes cells to palbociclib-induced senescence: a) Schematic depicting locations of the shRNA sequences on the autophagy-associated *Beclin-1* and *Atg5* genes. **b)** Crystal violet OD measured on day 12 from drug response studies as shown in Fig 2b. **c)** Palbociclib Half-maximal inhibitory concentrations (IC_{50}) values of MCF7 and T47D cells as calculated from the drug-response experiments shown in Fig. 2b. **d-f)** Beclin-1 or Atg5-knockdown MCF7 and T47D cells were treated with 0.5 or 1 μM of palbociclib (Palbo) for 6 days. Some cells were allowed to recover for 4 days (release) to examine reversibility and subjected to d) cell counting to assess proliferation (p-values were calculated in comparison to cells treated with Scramble shRNA [SCR] and 1 μM palbociclib), e) cell cycle analysis to determine the percentage change in G1 phase (p-values were calculated by comparing values at the end of drug treatment with those at the end of drug + release), and f) Representative images senescence-associated SA-ß gal–positive cells. All data represent mean ± SD from three independent experiments; ns: $p>0.05$; *$p<0.05$; **$p<0.01$; ***$p<0.001$; ****$p<0.0001$.

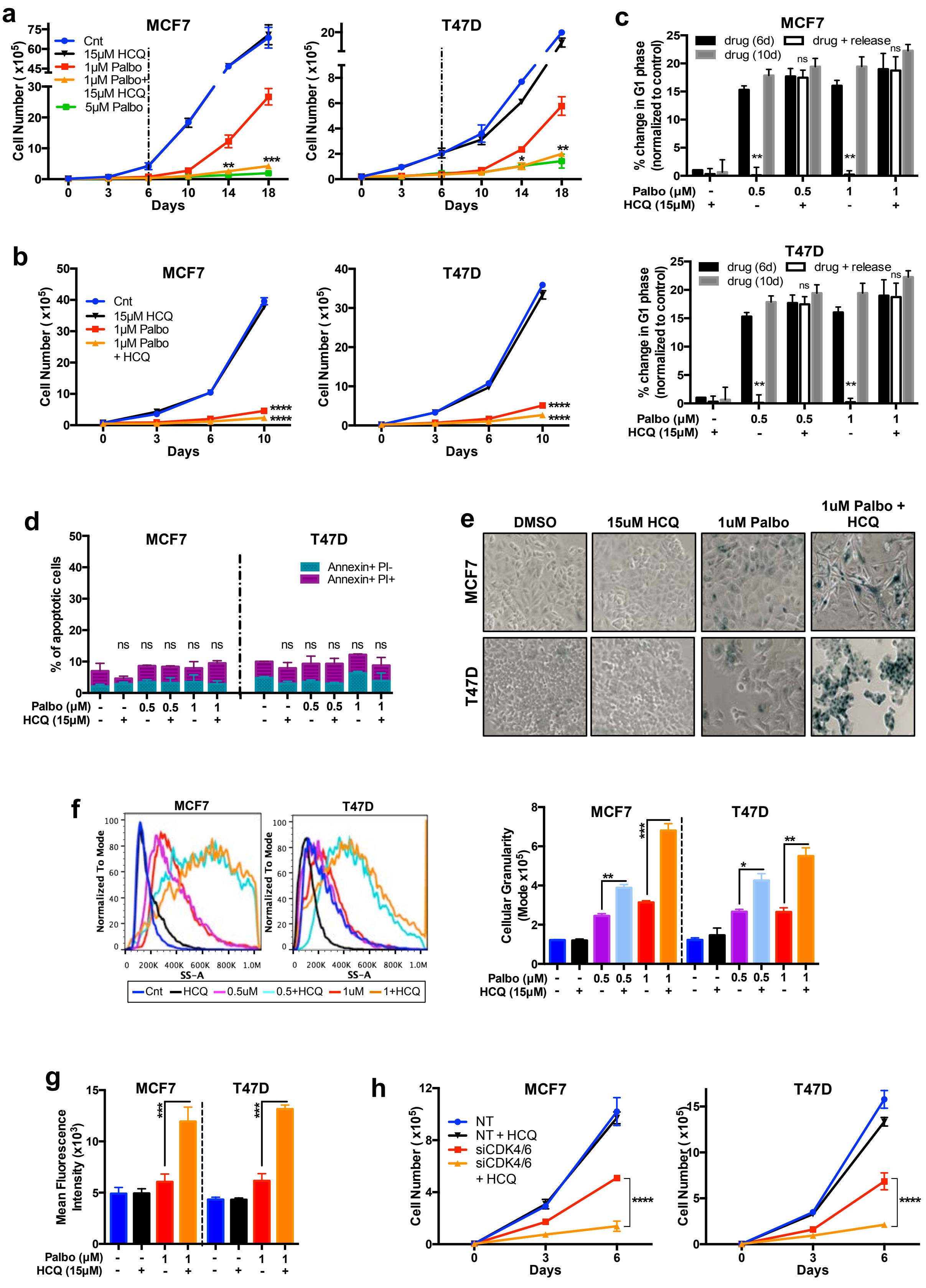
a
MCF7
T47D
Cnt
15μM HCQ
1μM Palbo
1μM Palbo+ 15μM HCQ
5μM Palbo
Cell Number (x10⁵)
Days
b
MCF7
T47D
Cnt
15μM HCQ
1μM Palbo
1μM Palbo + HCQ
Cell Number (x10⁵)
Days
c
MCF7
T47D
drug (6d)
drug + release
drug (10d)
% change in G1 phase (normalized to control)
Palbo (μM)
HCQ (15μM)
d
MCF7
T47D
Annexin+ PI-
Annexin+ PI+
% of apoptotic cells
Palbo (μM)
HCQ (15μM)
e
DMSO
15uM HCQ
1uM Palbo
1uM Palbo + HCQ
MCF7
T47D
f
MCF7
T47D
Normalized To Mode
SS-A
Cnt
HCQ
0.5uM
0.5+HCQ
1uM
1+HCQ
Cellular Granularity (Mode x10⁵)
Palbo (μM)
HCQ (15μM)
g
MCF7
T47D
Mean Fluorescence Intensity (x10³)
Palbo (μM)
HCQ (15μM)
h
MCF7
T47D
NT
NT + HCQ
siCDK4/6
siCDK4/6 + HCQ
Cell Number (x10⁵)
Days

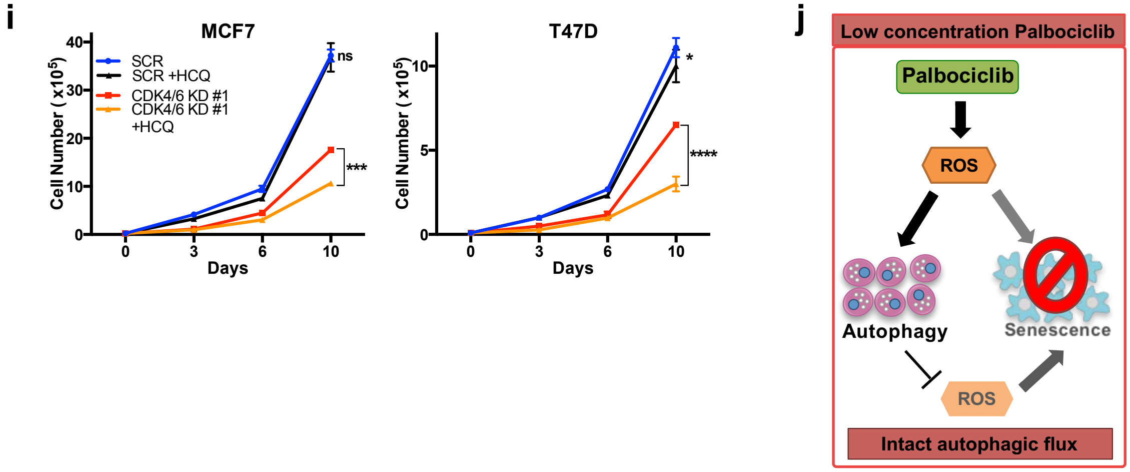

Supplementary Figure 8. Autophagy inhibition via hydroxychloroquine sensitizes cells to palbociclib-induced senescence: a,b) Effect on cell viability when treated with a combination of DMSO or palbociclib (Palbo - 0.5 or 1 µM) and autophagy inhibitor hydroxychloroquine (HCQ; 15 µM) for a) 6 days and allowed to recover for 12 days to examine long-term reversibility (p-values were calculated in comparison to cells treated with 1 µM palbociclib) and b) 12 days without drug washout. **c-g)** MCF7 and T47D cells were treated with 15 µM HCQ and/or palbociclib (0.5 or 1 µM) for 6 days and subjected to c) cell cycle analysis to determine the percentage change in G1 phase (p-values were calculated by comparing values at the end of 6 days drug treatment with those at the end of drug + release); d) flow cytometry measurement of apoptotic cells (early apoptosis: Annexin V+/PI-; late apoptosis: Annexin V+/PI+); e) representative images of SA-ß gal positive cells; f) measurement and quantification of side scatter analysis to assess cellular granularity; and g) quantification of cellular reactive oxygen species (ROS) levels. **h,i)** Proliferation of MCF7 and T47D cells with knockeddown of CDK4 and CDK6 via siRNA (h) or shRNA (i) and treatment with DMSO or 15 µM HCQ. **j)** Schematic showing the regulation of ROS production, senescence, and autophagy by palbociclib at low concentrations. All data represent mean ± SD from three independent experiments; ns: $p>0.05$; *$p<0.05$; **$p<0.01$; ***$p<0.001$; ****$p<0.0001$.

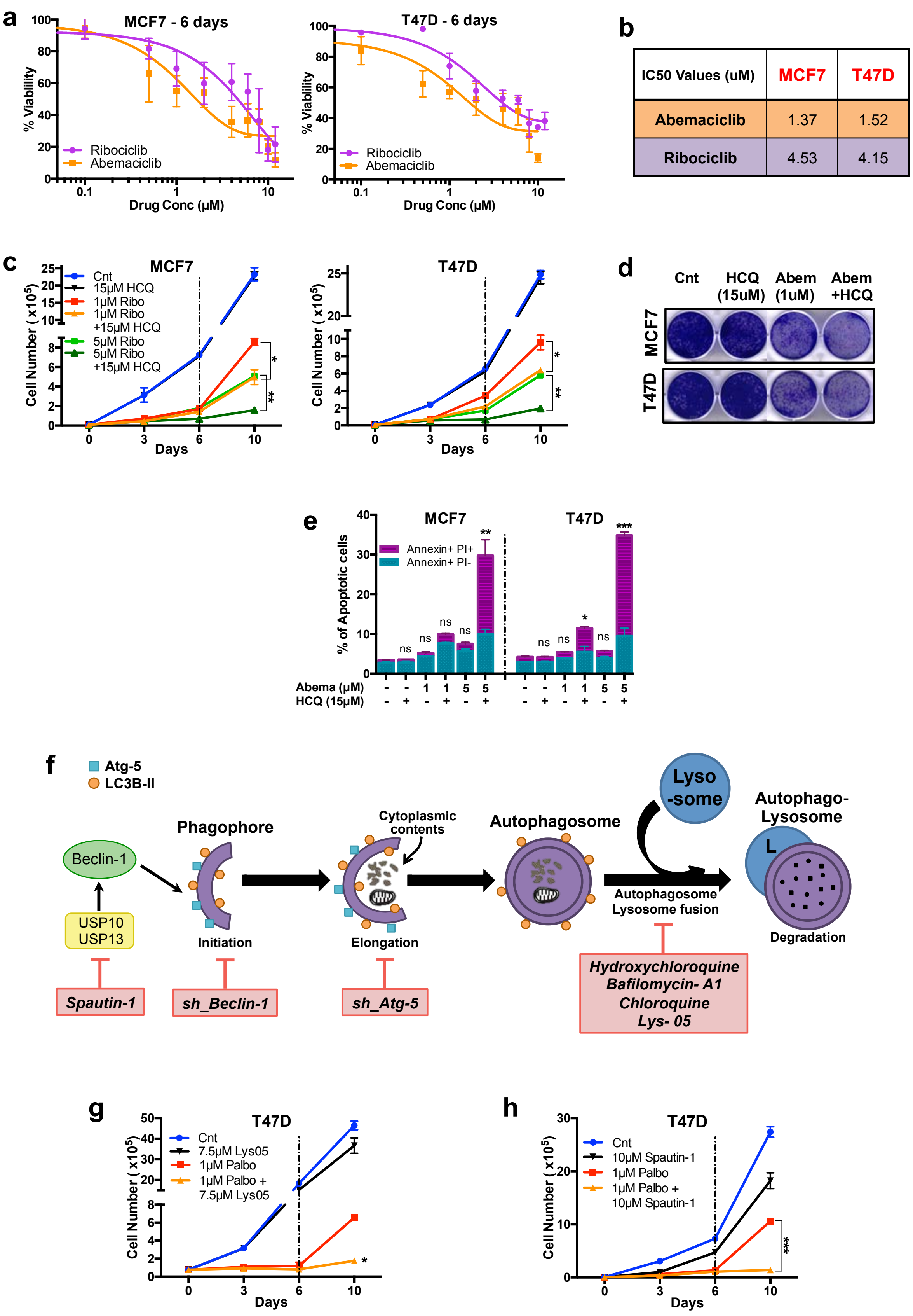
a
MCF7 - 6 days
% Viability
Drug Conc (µM)
Ribociclib
Abemaciclib
T47D - 6 days
% Viability
Drug Conc (µM)
Ribociclib
Abemaciclib
b
IC50 Values (uM)
MCF7
T47D
Abemaciclib 1.37 1.52
Ribociclib 4.53 4.15
c
MCF7
Cell Number (x10^5)
Days
Cnt
15µM HCQ
1µM Ribo
1µM Ribo +15µM HCQ
5µM Ribo
5µM Ribo +15µM HCQ
T47D
Cell Number (x10^5)
Days
d
Cnt
HCQ (15uM)
Abem (1uM)
Abem +HCQ
MCF7
T47D
e
MCF7
T47D
% of Apoptotic cells
Annexin+ PI+
Annexin+ PI-
Abema (µM)
HCQ (15µM)
f
Atg-5
LC3B-II
Beclin-1
USP10
USP13
Spautin-1
Phagophore
Initiation
sh_Beclin-1
Cytoplasmic contents
Elongation
sh_Atg-5
Autophagosome
Lyso -some
Autophagosome Lysosome fusion
Hydroxychloroquine
Bafilomycin- A1
Chloroquine
Lys- 05
Autophago- Lysosome
L
Degradation
g
T47D
Cell Number (x10^5)
Days
Cnt
7.5µM Lys05
1µM Palbo
1µM Palbo + 7.5µM Lys05
h
T47D
Cell Number (x10^5)
Days
Cnt
10µM Spautin-1
1µM Palbo
1µM Palbo + 10µM Spautin-1

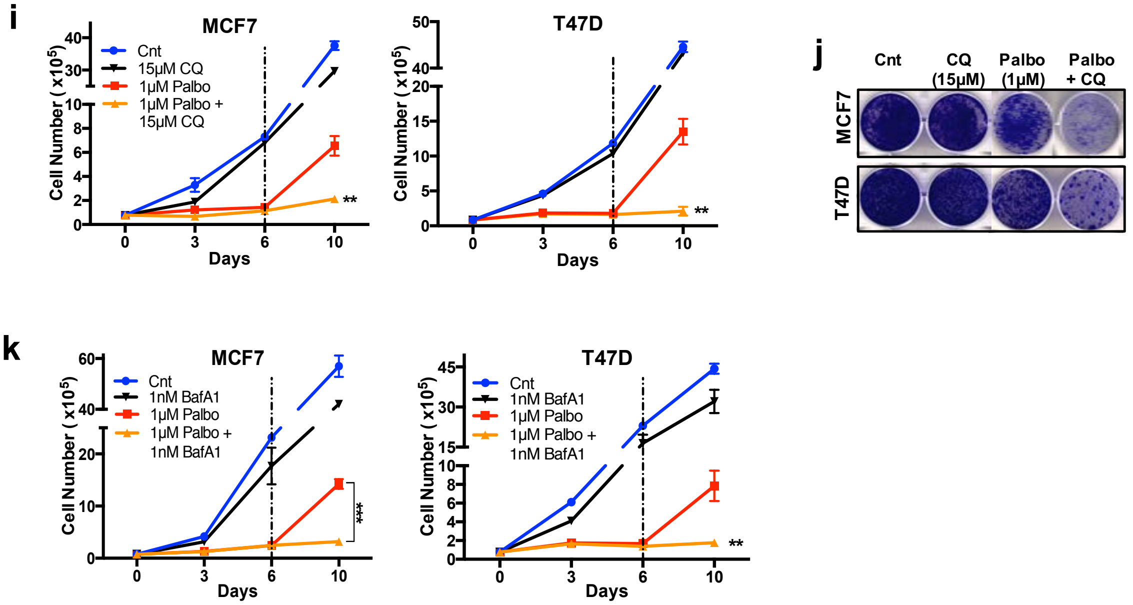

Supplementary Figure 9. Autophagy inhibition sensitizes cells to senescence induced by CDK4/6 inhibitors: a,b) Proliferation of MCF7 and T47D cells treated with DMSO or increasing concentrations (conc) of ribociclib or abemaciclib (0.01 to 12 µM) for 6 days (a) and their corresponding half-maximal inhibitory concentration (IC_{50}) values (b). **c)** Cell counting was used to assess proliferation of MCF7 and T47D cells treated with a combination of DMSO (Cnt) or ribociclib (1 or 5 µM) and 15 µM hydroxychloroquine (HCQ) for 6 days. Cells were allowed to recover for 4 days to examine reversibility. **d)** Clonogenic assay to evaluate proliferation upon combined treatment with 5 µM abemaciclib and HCQ for 6 days and recovery for 6 days. **e)** Flow cytometry measurement of apoptosis (early apoptosis: Annexin V+/PI-; late apoptosis: Annexin V+/PI+) in cells treated with 5µM abemaciclib combined with 15 µM HCQ for 6 days. p-values were calculated in comparison to cells treated with DMSO (Control). **f)** Schematic of the autophagy pathway indicating the points of genetic and pharmacological interventions described in Fig. 2. **g,h)** Cell counting was used to assess proliferation of T47D cells treated with a combination of 1µM palbociclib and 7.5 µM Lys-05 (g) or 10 µM Spautin-1 (h) for 6 days and allowed to recover for 4 days. **i,j)** MCF7 and T47D cells were treated with 1µM palbociclib and 15µM chloroquine (CQ) for 6 days and allowed to recover for 4 or 6 days The effects on growth were examined by i) cell counting and j) clonogenic assay. **k)** Cell counting to assess proliferation of MCF7 and T47D cells treated with the combination of 1 µM palbociclib and the autophagy inhibitor Bafilomycin (BafA1- 1 nM) for 6 days. Cells were allowed to recover for 4 days to examine reversibility. All data represent mean ± SD from three independent experiments; ns: p>0.05; *p<0.05; **p<0.01; ***p<0.001; ****p<0.0001.

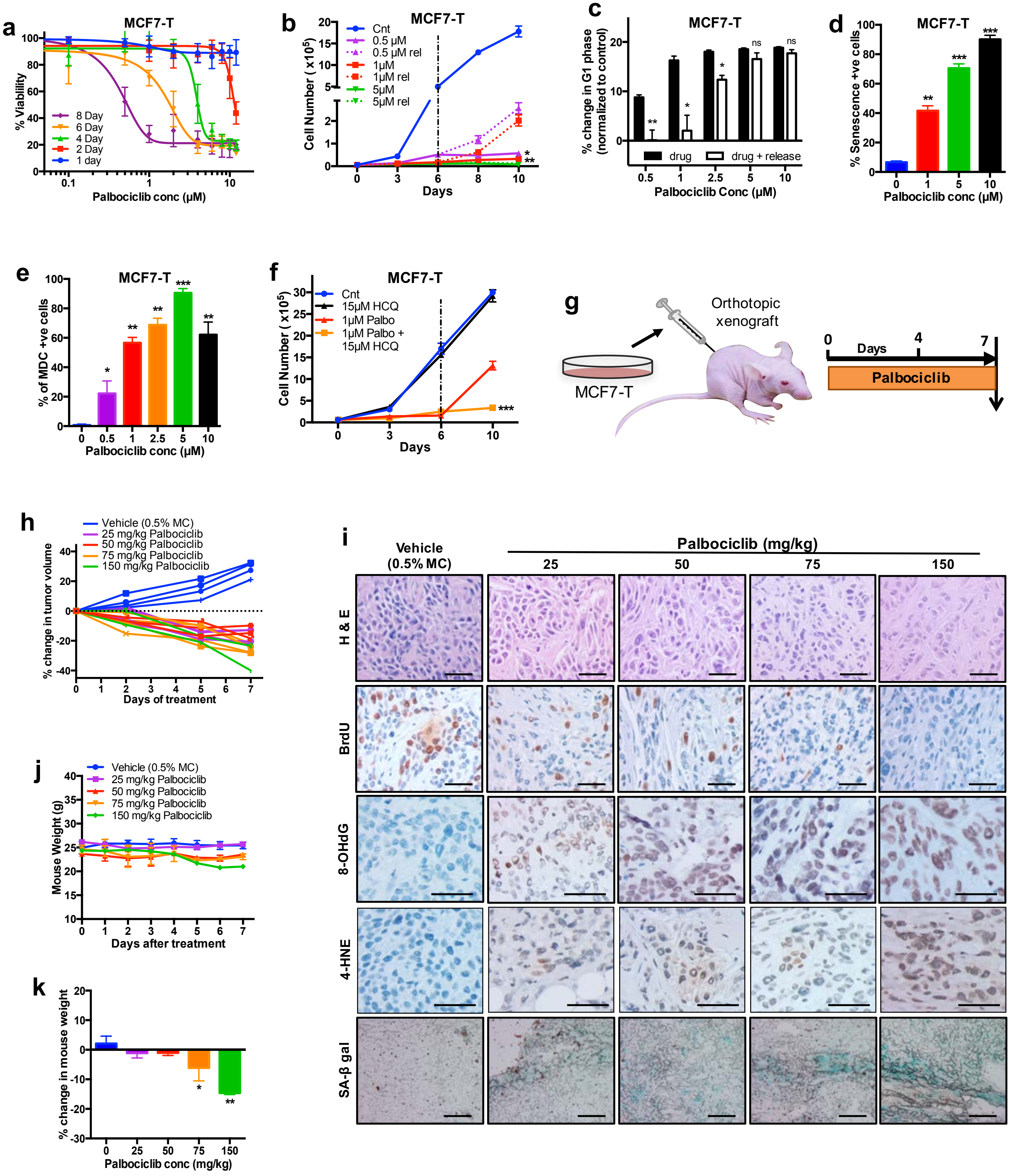
a
MCF7-T
% Viability
8 Day
6 Day
4 Day
2 Day
1 day
Palbociclib conc (μM)
b
MCF7-T
Cell Number (x10^5)
Cnt
0.5 μM
0.5 μM rel
1μM
1μM rel
5μM
5μM rel
Days
c
MCF7-T
% change in G1 phase (normalized to control)
drug
drug + release
Palbociclib Conc (μM)
d
MCF7-T
% Senescence +ve cells
Palbociclib conc (μM)
e
MCF7-T
% of MDC +ve cells
Palbociclib conc (μM)
f
MCF7-T
Cell Number (x10^5)
Cnt
15μM HCQ
1μM Palbo
1μM Palbo + 15μM HCQ
Days
g
Orthotopic xenograft
MCF7-T
Days
Palbociclib
h
Vehicle (0.5% MC)
25 mg/kg Palbociclib
50 mg/kg Palbociclib
75 mg/kg Palbociclib
150 mg/kg Palbociclib
% change in tumor volume
Days of treatment
i
Vehicle (0.5% MC)
Palbociclib (mg/kg)
25
50
75
150
H & E
BrdU
8-OHdG
4-HNE
SA-β gal
j
Vehicle (0.5% MC)
25 mg/kg Palbociclib
50 mg/kg Palbociclib
75 mg/kg Palbociclib
150 mg/kg Palbociclib
Mouse Weight (g)
Days after treatment
k
% change in mouse weight
Palbociclib conc (mg/kg)

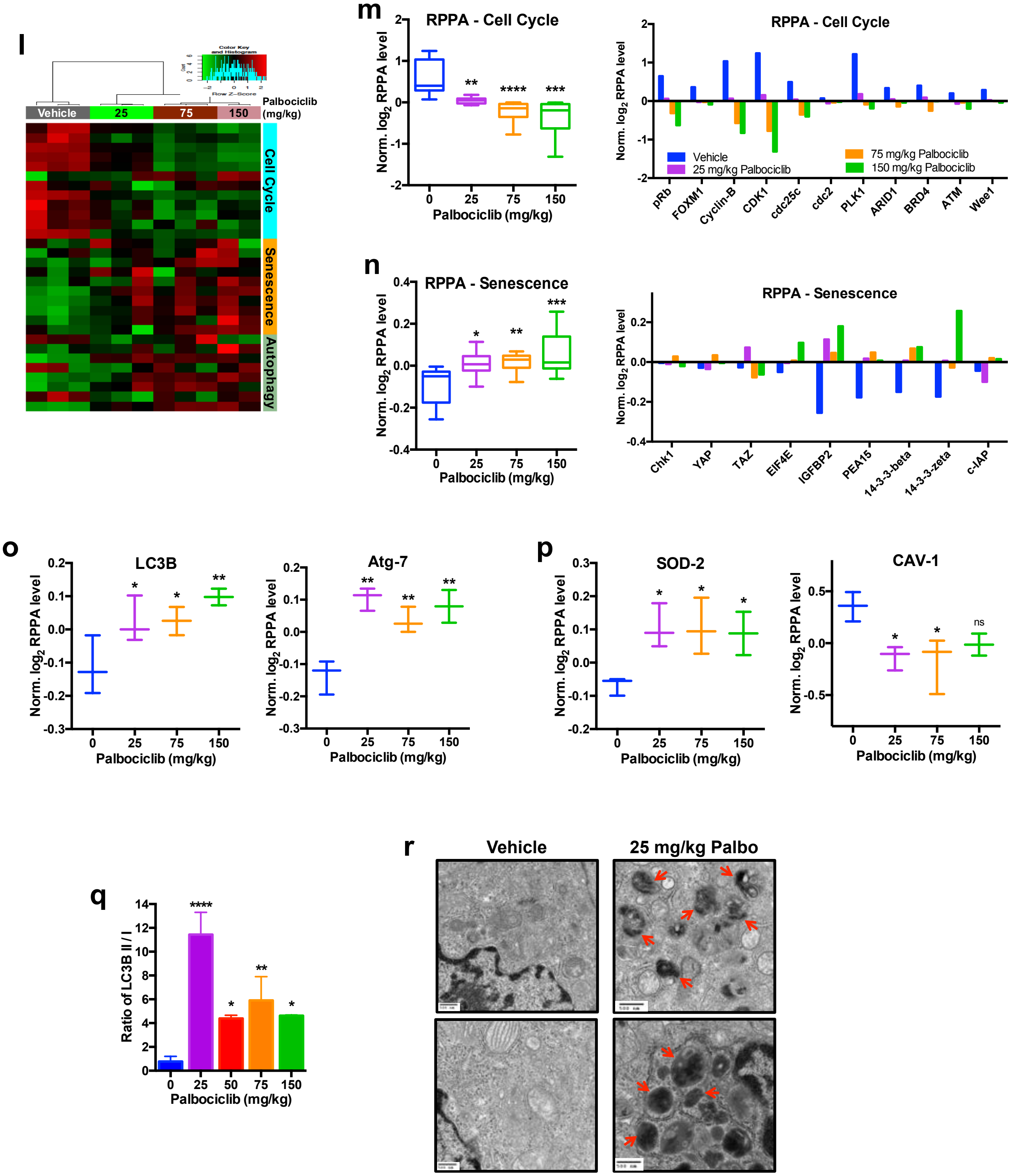
l
Color Key and Histogram
Row Z-Score
Vehicle
25
75
150
Palbociclib (mg/kg)
Cell Cycle
Senescence
Autophagy
m
RPPA - Cell Cycle
Norm. log2 RPPA level
Palbociclib (mg/kg)
0
25
75
150
**
****
***
RPPA - Cell Cycle
Vehicle
25 mg/kg Palbociclib
75 mg/kg Palbociclib
150 mg/kg Palbociclib
pRb
FOXM1
Cyclin-B
CDK1
cdc25c
cdc2
PLK1
ARID1
BRD4
ATM
Wee1
n
RPPA - Senescence
*
**
***
RPPA - Senescence
Chk1
YAP
TAZ
EIF4E
IGFBP2
PEA15
14-3-3-beta
14-3-3-zeta
c-IAP
o
LC3B
Atg-7
p
SOD-2
CAV-1
ns
q
Ratio of LC3B II / I
****
**
*
0
25
50
75
150
Palbociclib (mg/kg)
r
Vehicle
25 mg/kg Palbo

Supplementary Figure 10. Palbociclib induces autophagy *in vivo*: a) Proliferation of MCF7-T cells treated with DMSO or increasing concentrations (conc) of palbociclib (0.01 to 12 µM) for 1, 2, 4, 6, or 8 days. **b-d)** MCF7-T cells were treated with DMSO (Cnt) or varying concentrations (0.5, 1, or 5 µM) of palbociclib for 6 days and allowed to recover for 4 days (release, or rel) to examine reversibility. Cells were then subjected to b) cell counting to assess the effect on growth; c) cell cycle analysis to determine percentage change in G1 phase (p-values were calculated by comparing values at the end of drug treatment with those at the end of drug + release); and d) quantification of senescence-associated SA-ß gal positive cells. **e)** Monodansylcadavarine (MDC) positive MCF7-T cells were quantified with flow cytometry after treatment with DMSO or varying concentrations of palbociclib for 6 days. **f)** Cell counting was used to assess effect on growth of MCF7-T cells treated with a combination of DMSO or palbociclib (Palbo; 0.5 or 1 µM) and autophagy inhibitor hydroxychloroquine (HCQ; 15 µM) for 6 days. Cells were allowed to recover for 4 days to examine reversibility. **g)** Schematic showing treatment schedule of orthotopic MCF7-T xenograft mice with vehicle (0.5% methylcellulose [MC]) or varying concentrations of palbociclib for 7 days. Images of the mouse and the syringe were taken with permission from ScienceSlides by VisiScience Corp. **h)** Percentage change in tumor volume of orthotopic xenograft mice (normalized to volume on Day 0) of individual tumors treated with vehicle (0.5% methylcellulose) or varying concentrations of palbociclib daily for 7 days via oral gavage ($n \geq 4$ mice tumors/treatment group). **i)** Representative images of hematoxylin and eosin (H&E), BrdU, 8-hydroxydeoxy-guanosine (8-OHdG), 4-hydroxynonenal (4-HNE) and SA-ß gal immunohistochemical staining of tumor tissues harvested after 7 days of treatment as described in (h). Scale bars equal 50µm. **j)** Mean mouse weights for each treatment group after 7 days of treatment as described in (h). **k)** Percentage change in mouse weights after 7 days treatment as described in (h). **l)** Heat map obtained from RPPA analysis of tumors harvested after treated as in (h) and ordered based on Cell cycle, senescence and autophagy pathways. **m,n)** Pathway scores and expression (normalized $\log_2$ level) of individual proteins within the m) cell cycle (n=10 proteins) and n) senescence pathways (n=13 proteins) determined from RPPA analysis of tumors harvested after treatment as described in (h). **o)** Expression (normalized $\log_2$ level) of LC3B and Atg-7 protein levels as determined from RPPA analysis of tumors harvested after treatment as described in (h). **p)** Expression (normalized $\log_2$ level) of SOD-2 and Cav-1 protein levels as determined from RPPA analysis of tumors harvested after treatment as described in (h). **q)** Autophagic flux, calculated as ratio of LC3B-II to LC3B-I from densitometry values (normalized to the corresponding levels of actin) of western blots from Fig. 3c. **r)** Representative TEM microphotographs of tumors harvested from mice treated with vehicle (0.5% MC), 25 mg/kg or 150 mg/kg of palbociclib for 7 days. Red arrows indicate double-membraned autophagosomes. Scale bars equal 500 nm. All in vitro data represent mean±SD from three independent experiments; p-values were calculated in comparison treatement with DMSO or vehicle (Control) unless indicated. ns: $p>0.05$; *$p<0.05$; **$p<0.01$; ***$p<0.001$; ****$p<0.0001$.

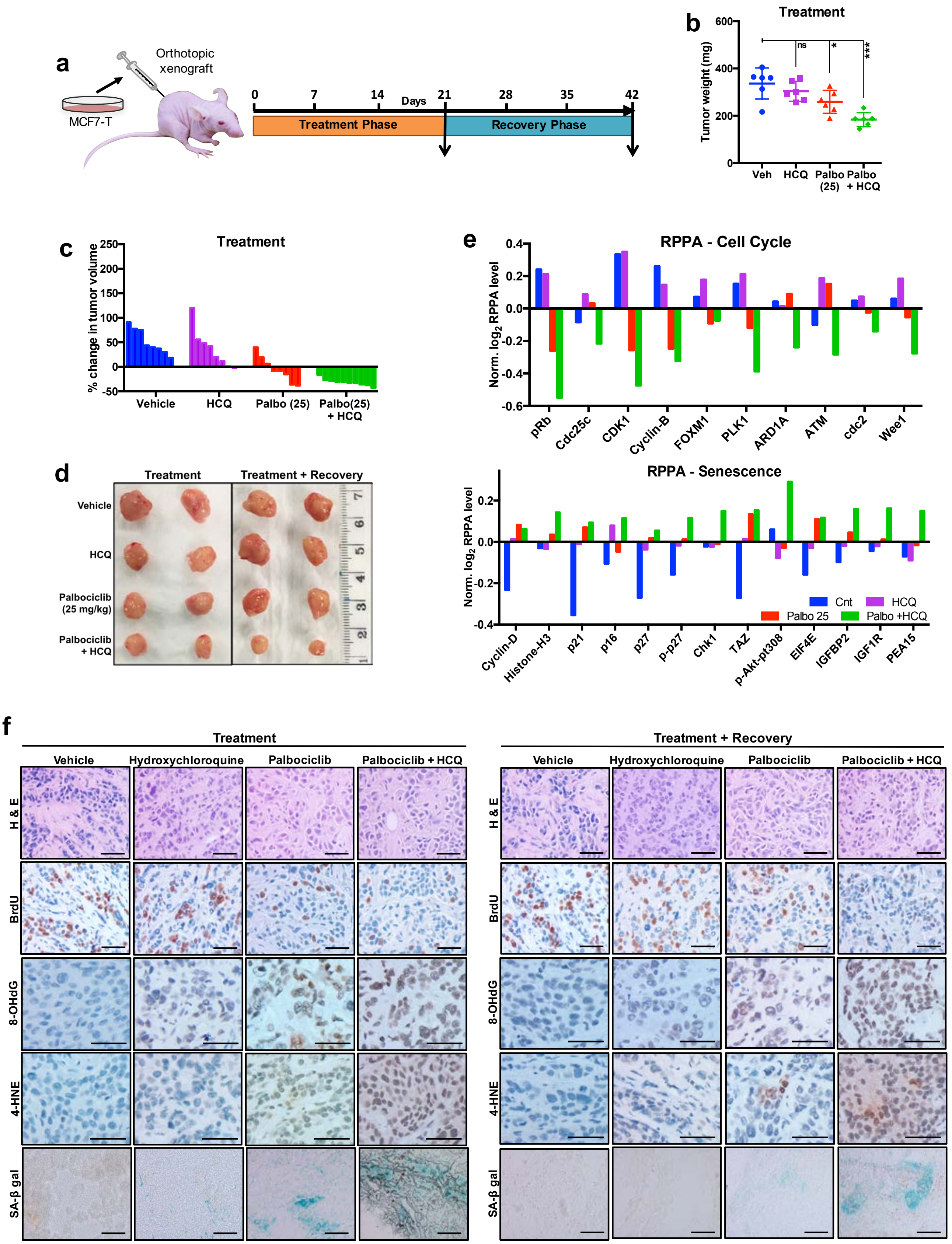
a
Orthotopic xenograft
MCF7-T
Days
0 7 14 21 28 35 42
Treatment Phase
Recovery Phase
b
Treatment
Tumor weight (mg)
ns
*
***
Veh
HCQ
Palbo (25)
Palbo + HCQ
c
Treatment
% change in tumor volume
Vehicle
HCQ
Palbo (25)
Palbo(25) + HCQ
d
Treatment
Treatment + Recovery
Vehicle
HCQ
Palbociclib (25 mg/kg)
Palbociclib + HCQ
e
RPPA - Cell Cycle
Norm. log2 RPPA level
pRb
Cdc25c
CDK1
Cyclin-B
FOXM1
PLK1
ARD1A
ATM
cdc2
Wee1
RPPA - Senescence
Cyclin-D
Histone-H3
p21
p16
p27
p-p27
Chk1
TAZ
p-Akt-pt308
EIF4E
IGFBP2
IGF1R
PEA15
Cnt
HCQ
Palbo 25
Palbo +HCQ
f
Treatment
Treatment + Recovery
Vehicle
Hydroxychloroquine
Palbociclib
Palbociclib + HCQ
H & E
BrdU
8-OHdG
4-HNE
SA-β gal

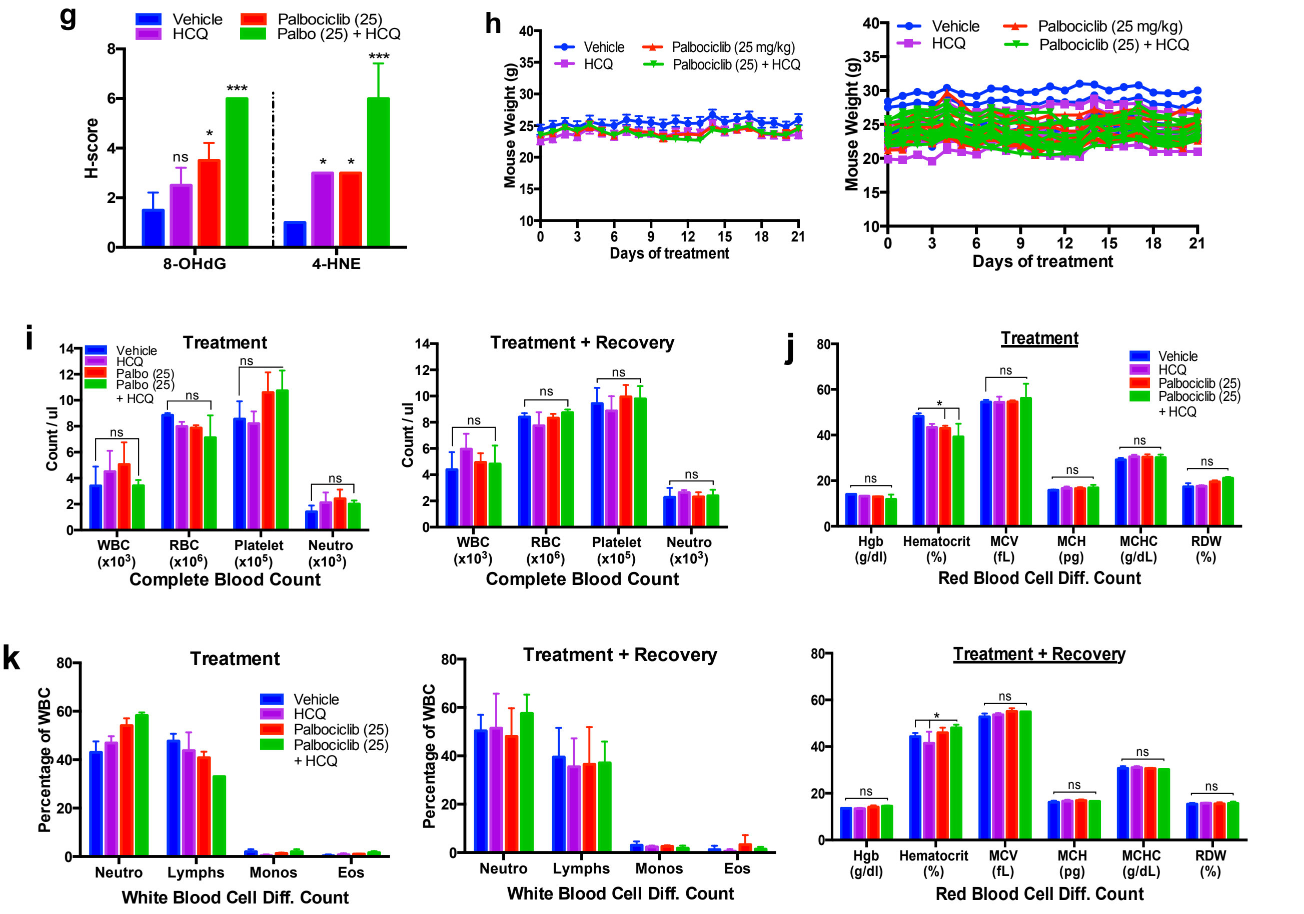

Supplementary Figure 11. Palbociclib synergizes with hydroxychloroquine to inhibit tumor growth *in vivo*: a) Schematic showing treatment schedule of orthotopic MCF7-T xenograft mice with vehicle (veh; 0.5% methylcellulose and PBS), 60 mg/kg hydroxychloroquine (HCQ), 25 mg/kg palbociclib (Palbo); or combination of palbociclib (25mg/kg) and hydroxychloroquine (60mg/kg) for 21 days (treatment phase), followed by a recovery phase of 21 days without treatment. Images of the mouse and the syringe were taken with permission from ScienceSlides by VisiScience Corp. **b)** Tumor weights at the end of treatment phase (21 days) as described in (a) (n=6 mice/treatment group). **c)** Percentage change in tumor volume of individual tumors at the end of the treatment phase as described in (a). **c)** Representative images of tumors harvested at the end of the treatment phase and at the end of the recovery phase as described in (a). **e)** Expression (normalized $\log_2$ expression level) of individual proteins of the cell cycle (n=10 proteins) and senescence pathways (n=13 proteins) determined from RPPA analysis of tumors harvested after treatment as described in (a). **f)** Representative images of hematoxylin and eosin (H&E), BrdU, 8-hydroxydeoxy-guanosine (8-OHdG), 4-hydroxynonenal (4-HNE) and senescence-associated SA-ß gal immunohisto-chemical staining on tumor tissues harvested at the end of the treatment phase and at the end of the recovery phase as described in (a). Scale bars equal 50 µm. **g)** Quantification (H-score) of 8-OHdG and 4-HNE staining on tumors at the end of treatment + recovery phase. **h)** Mean mouse weight and Weight of individual mice after 21 days of treatment as described in (a). **i-k)** Mice were treated as described in (a). Blood samples collected at the end of treatment phase and at the end of the treatment + recovery phase were subjected to i) complete blood counts (red blood cells [RBC], white blood cells [WBC], platelets, and neutrophils [Neutro]); j) RBC differential counts; and k) WBC differential count. All data represent mean±SD; p-values were calculated in comparison to mice treated with vehicle (Control) unless indicated. ns:p>0.05; *p<0.05; **p<0.01; ***p<0.001; ****p<0.0001.

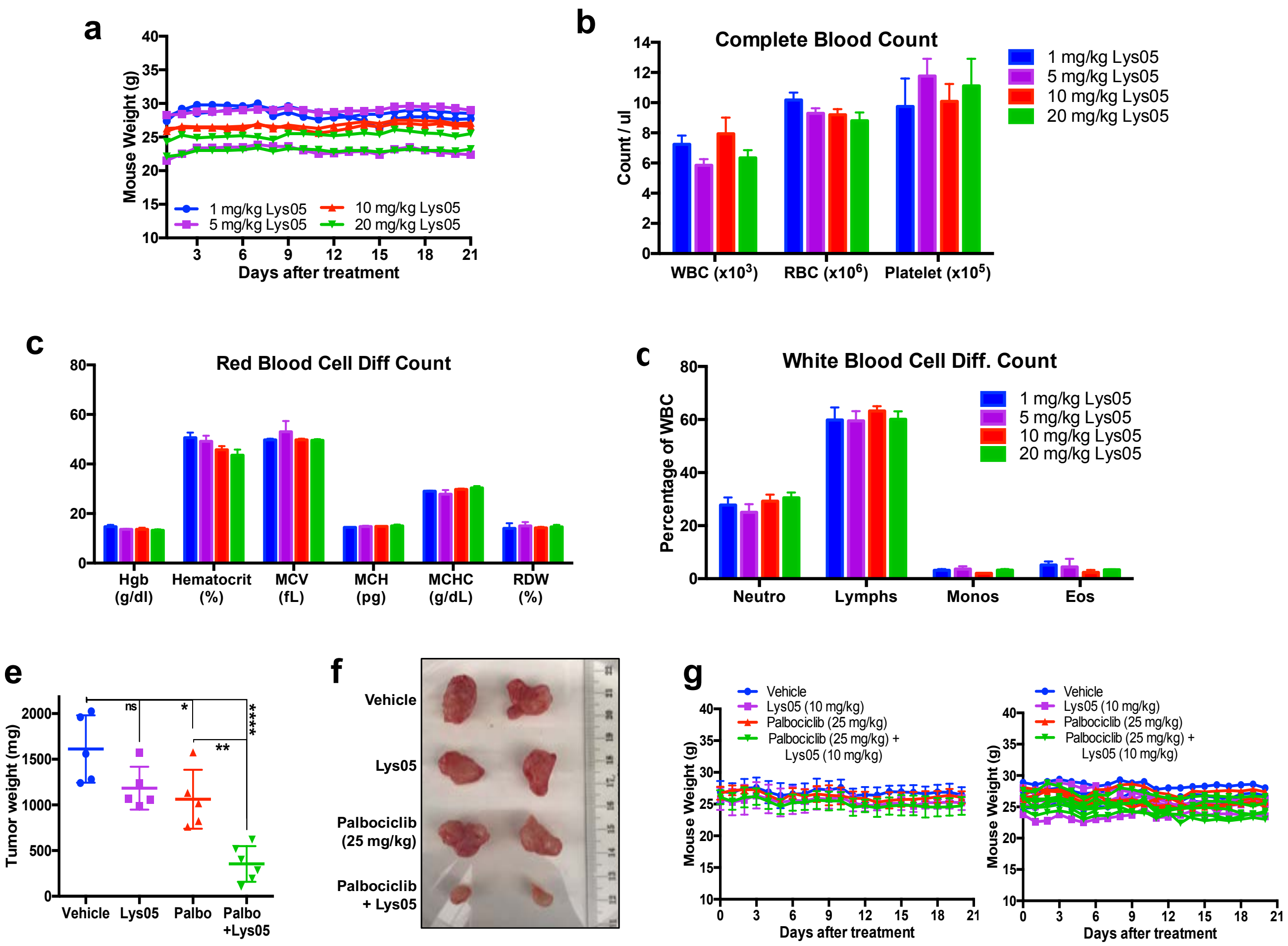

Supplementary Figure 12. Palbociclib synergizes with the autophagy inhibitor, Lys-05 to inhibit tumor growth *in vivo*: a) Weight of individual non-tumor bearing mice treated with varying concentrations of Lys-05 daily for 21 days via I.P (n=2 mice/treatment group). **b-d)** Mice were treated as described in (a). Blood samples collected at the end of treatment were subjected to b) complete blood counts (red blood cells [RBC], white blood cells [WBC], platelets; c) RBC differential counts; and d) WBC differential count. **e,f)** Tumor weights (e) and representative pictures (f) after treatment of orthotopic MCF7-T xenograft mice with vehicle (veh; 0.5% methylcellulose and PBS),160 mg/kg Lys-05, 25 mg/kg palbociclib (Palbo); or combination of palbociclib (25mg/kg) and Lys-05 (10mg/kg) for 21 days (treatment phase), followed by a recovery phase of 14 days without treatment (n≥5 mice tumors/treatment group). **g)** Mean mouse weight and weight of individual mice after 21 days of treatment as described in (e). All data represent mean±SD; p-values were calculated in comparison to mice treated with vehicle (Control) unless indicated. ns:p>0.05; *p<0.05; **p<0.01; ***p<0.001; ****p<0.0001.

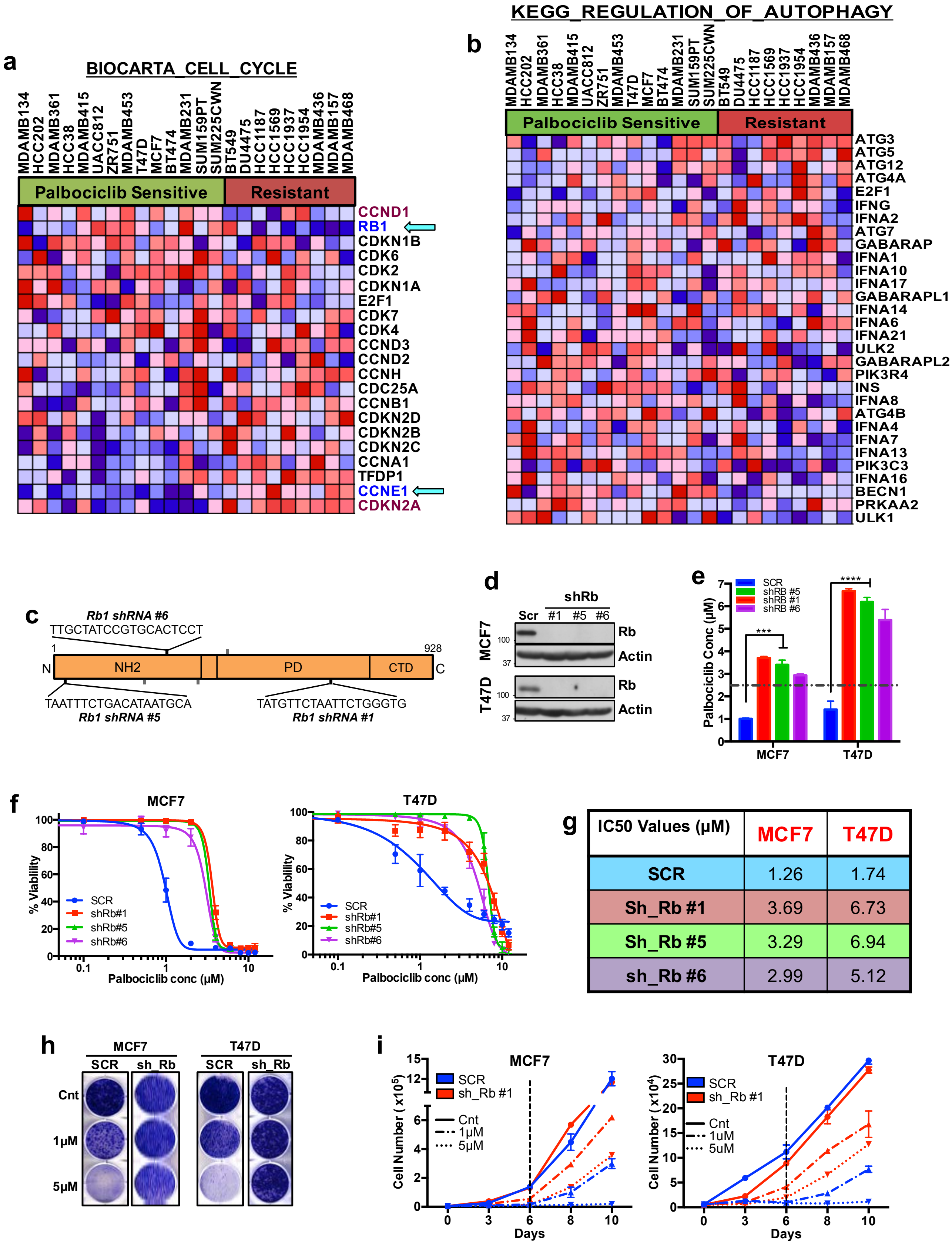

g

IC50 Values (µM)	MCF7	T47D
SCR	1.26	1.74
Sh_Rb #1	3.69	6.73
Sh_Rb #5	3.29	6.94
sh_Rb #6	2.99	5.12

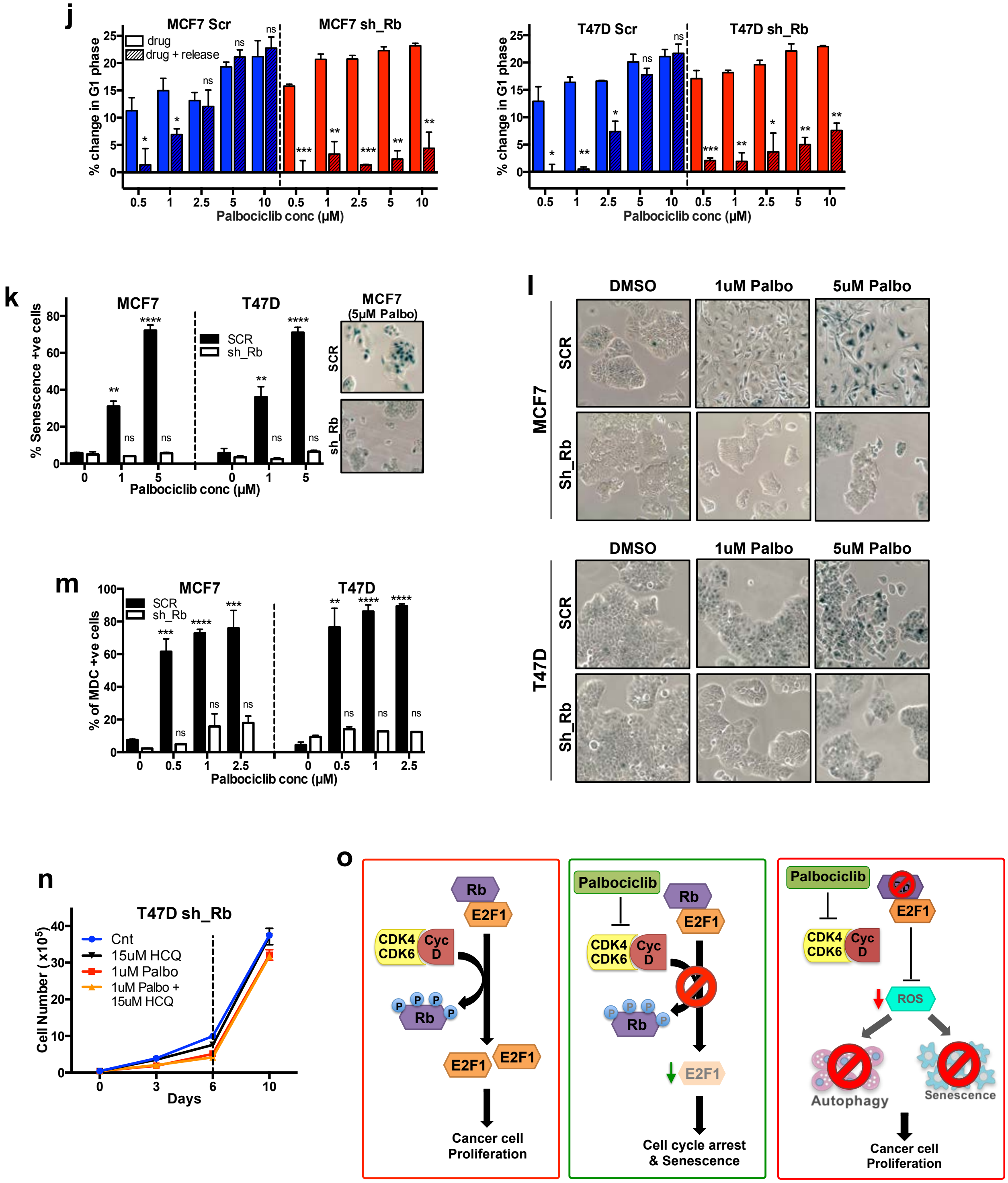

j
MCF7 Scr
MCF7 sh_Rb
drug
drug + release
% change in G1 phase
Palbociclib conc (μM)
T47D Scr
T47D sh_Rb
k
MCF7
T47D
SCR
sh_Rb
% Senescence +ve cells
Palbociclib conc (μM)
MCF7
(5μM Palbo)
l
DMSO
1uM Palbo
5uM Palbo
MCF7
SCR
Sh_Rb
T47D
m
MCF7
T47D
SCR
sh_Rb
% of MDC +ve cells
Palbociclib conc (μM)
n
T47D sh_Rb
Cnt
15uM HCQ
1uM Palbo
1uM Palbo +
15uM HCQ
Cell Number (x10^5)
Days
o
Rb
E2F1
CDK4
CDK6
Cyc
D
Cancer cell
Proliferation
Palbociclib
Cell cycle arrest
& Senescence
ROS
Autophagy
Senescence
Cancer cell
Proliferation

Supplementary Figure 13. Deregulated Rb predicts resistance to combination of palbociclib and autophagy inhibition in ER positive breast cancer: a,b) Heat map constructed using the Gene Set Enrichment Analysis (GSEA) program, denoting expression of genes from the Biocarta Cell Cycle (a) and KEGG Regulation of autophagy (b) gene sets in the indicated breast cancer cell lines, classified as being sensitive or resistant to palbociclib. **c)** Schematic depicting locations of the shRNA sequences on the *Rb1* gene. **d)** Western blot showing levels of Rb protein in MCF7 and T47D cells after transfection with shRNA for Scrambled (Scr) or Rb. **e-g)** Impact of knocking down Rb on the growth of MCF7 and T47D treated with DMSO or increasing concentrations (conc) of palbociclib (0.01 to 12 µM) for 6 days (f) and their corresponding half-maximal inhibitory concentration (IC_{50}) values (e,g). **h)** Clonogenic assay in Rb-knockdown MCF7 and T47D cells treated with DMSO (Cnt) or palbociclib (1 or 5 µM) for 6 days and allowed to recover for 6 days. **i-m)** Rb-knockdown MCF7 and T47D cells were treated with varying concentrations of palbociclib (Palbo) for 6 days and allowed to recover for 4 days (release) to examine reversibility. Cells were then subjected to i) cell counting to assess proliferation; j) cell cycle analysis to determine the percentage change in G1 phase (p-values were calculated by comparing values at the end of drug treatment with those at the end of release); k,l) Senescence activity by SA-ß-gal assay (k) and representative images (l); and m) flow cytometry to quantify monodansylcadavarine (MDC) staining, a marker of autophagic vesicles. **n)** Cell counting to assess proliferation of Rb-knockdown T47D cells treated with the combination of 1µM palbociclib and 15µM hydroxychloroquine for 6 days and allowed to recover for 4 days. **o)** Schematic depicting the role of Rb in regulating the ability of palbociclib to induce reactive oxygen species (ROS), autophagy, and senescence. All data represent mean±SD from three independent experiments; p-values were calculated in comparison to cells treated with DMSO (Control) unless indicated. ns: p>0.05; *p<0.05; **p<0.01; ***p<0.001; ****p<0.0001.

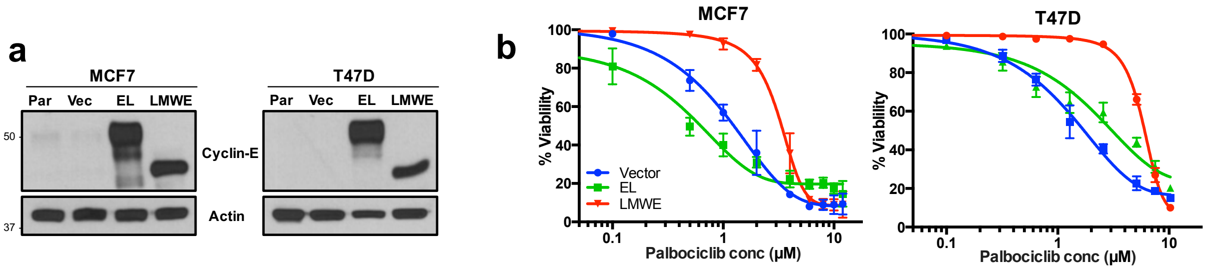

c

IC50 Values (µM)	MCF7		T47D	
	6 day	8 day	6 day	8 day
Vector	1.18	0.54	1.63	0.69
EL	0.69	0.30	2.37	1.48
LMW-E	3.76	2.54	5.82	5.69

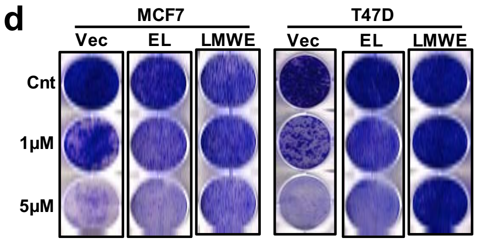

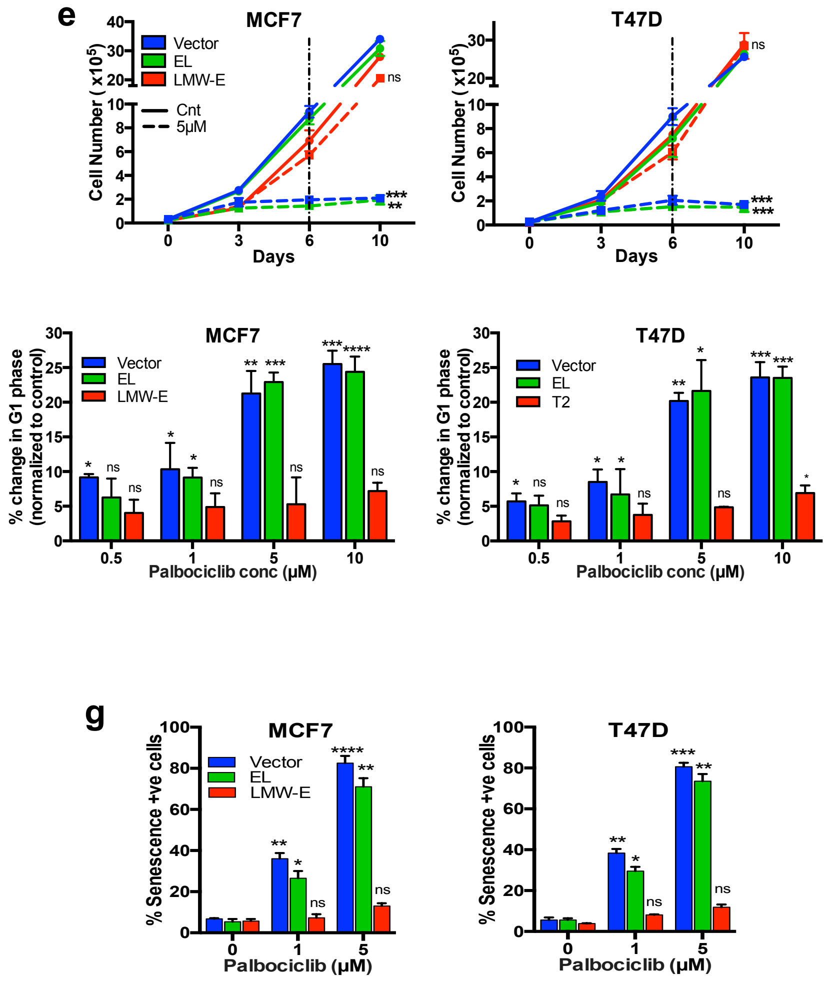

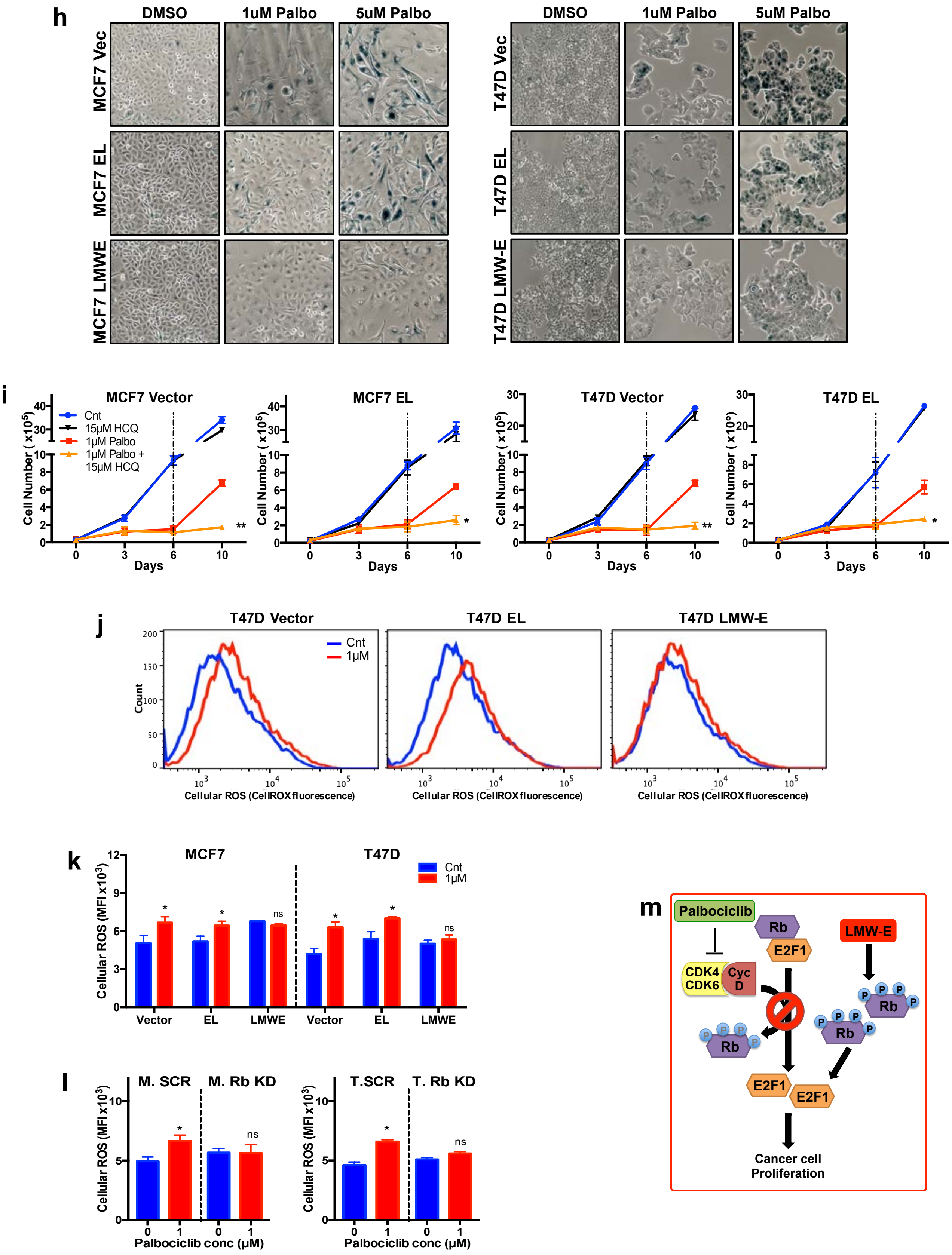

h
DMSO
1uM Palbo
5uM Palbo
MCF7 Vec
MCF7 EL
MCF7 LMWE
T47D Vec
T47D EL
T47D LMW-E
i
MCF7 Vector
MCF7 EL
T47D Vector
T47D EL
Cnt
15μM HCQ
1μM Palbo
1μM Palbo + 15μM HCQ
Cell Number (x10^5)
Days
**
*
j
T47D Vector
T47D EL
T47D LMW-E
Cnt
1μM
Count
Cellular ROS (CellROX fluorescence)
k
MCF7
T47D
Cnt
1μM
Cellular ROS (MFI x10^3)
Vector
EL
LMWE
ns
l
M. SCR
M. Rb KD
T.SCR
T. Rb KD
Cellular ROS (MFI x10^3)
Palbociclib conc (μM)
m
Palbociclib
Rb
E2F1
LMW-E
CDK4
CDK6
Cyc D
P
Cancer cell Proliferation

Supplementary Figure 14. Low-molecular-weight isoforms of cyclin E predict resistance to the combination of palbociclib and autophagy inhibition in ER positive breast cancer: a) Western blot showing levels of cyclin E protein in MCF7 and T47D cells stably overexpressing full-length cyclin E (EL) or low-molecular-weight isoforms of cyclin E (LMW-E). Par, parental cells; Vec, vector-transfected cells. **b,c)** Impact of overexpressing vector, EL, or LMW-E on the growth of MCF7 and T47D cells treated with DMSO or increasing concentrations (conc) of palbociclib (0.01 to 12 µM) for 6 days (b) and their corresponding half-maximal inhibitory concentration (IC_{50}) values (c). **d-h)** MCF7 and T47D cells overexpressing vector, EL, or LMW-E were treated with DMSO (Cnt) or varying concentrations of palbociclib (Palbo) for 6 days. Cells were allowed to recover for 4 days to examine reversibility, at which point they were subjected to d) clonogenic assay; e) cell counting to assess cell proliferation; f) cell cycle analysis to examine the change in G1 phase; g,h) SA-ß gal staining to assess senescence (representative images are shown in h). **i)** Cell counting was used to assess growth of vector, EL, or LMW-E overexpressing MCF7 and T47D cells treated with DMSO or palbociclib +15µM hydroxychloroquine (HCQ) for 6 days and allowed to recover for 4 days. **j,k)** Cellular reactive oxygen species (ROS) levels and its quantification (mean fluorescence intensity – MFI) measured in vector, EL, or LMW-E overexpressiong MCF7 or T47D cells treated with DMSO or palbociclib (1 µM or 5 µM) for 6 days. **l)** Quantification of ROS levels by MFI in Scramble (Scr) or Rb-knockdown (KD) MCF7 and T47D cells treated with DMSO or palbociclib (1 µM or 5 µM) for 6 days. **m)** Schematic depicting the mechanism by which LMW-E mediates resistance to palbociclib. All data represent mean±SD from three independent experiments; p-values were calculated in comparison to cells treated with DMSO (Control) unless indicated. ns:p>0.05; *p<0.05; **p<0.01; ***p<0.001; ****p<0.0001.

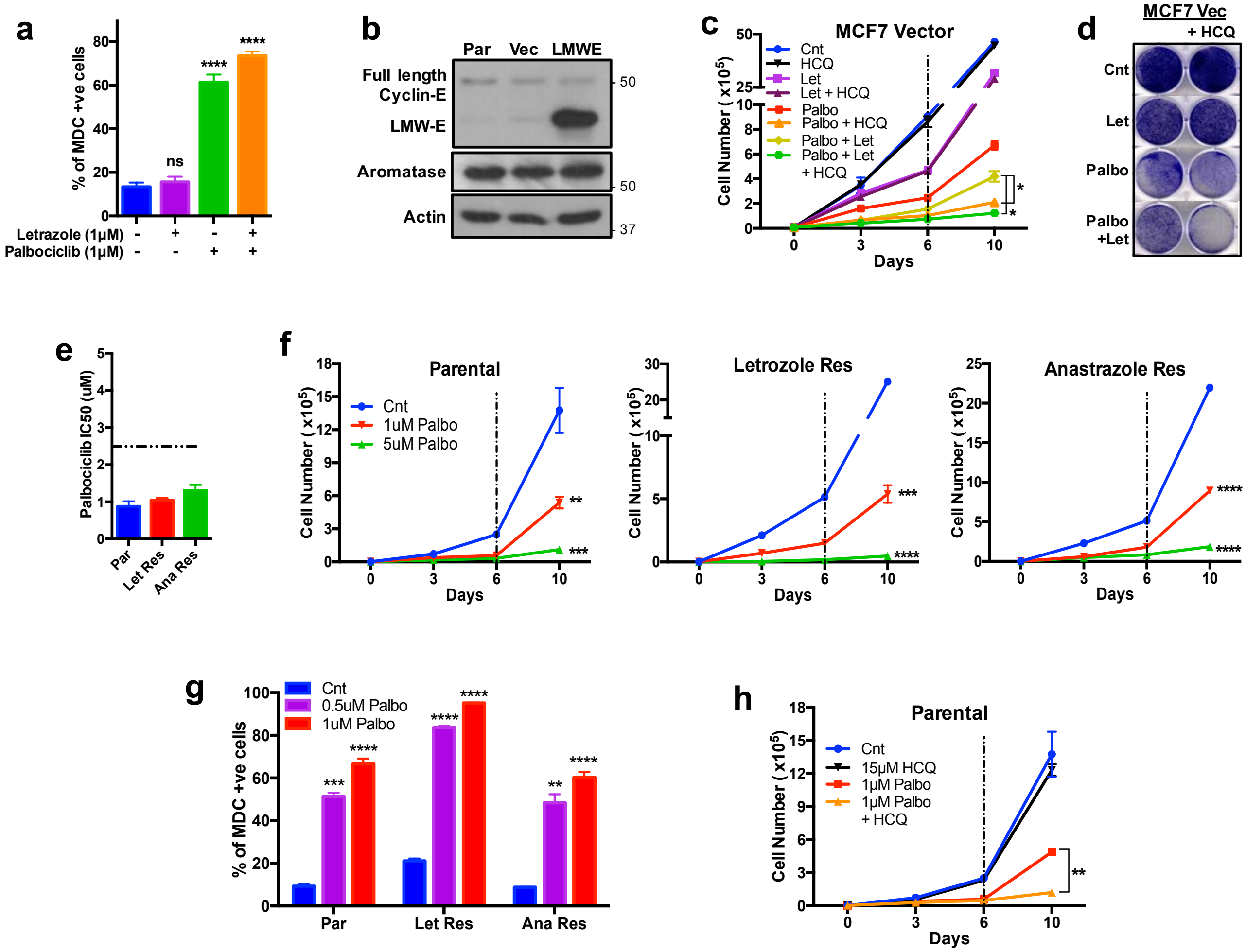

Supplementary Figure 15. Combination of palbociclib and autophagy inhibition is synergistic in aromatase expressing and aromatase inhibitor resistant cells: a) Measurement of monodansyl-cadavarine (MDC) positive acidic vesicles, including autophagosomes, by flow cytometry in MCF7 aromatase expressing cells treated with 1 μM palbociclib in combination with 1 μM letrozole for 6 days. **b)** Western blot showing levels of cyclin E and aromatse protein in MCF7 cells stably overexpressing aromatase (Par) and empty vector (Vec) or low-molecular-weight isoforms of cyclin E (LMW-E). **c,d)** Cell counting (c) and clonogenic assay (d) to measure impact of overexpressing empty vector in MCF7 aromatase expressing cells treated with 1 μM palbociclib (Palbo), 1 μM letrozole (Let) and 15 μM hydroxychloroquine (HCQ) in the indicated combiantions for 6 days. **e)** Half-maximal inhibitory concentration (IC_{50}) values of palbociclib in MCF7 parental, letrazole or anastrazole resistant cells treated with increasing concentrations (conc) of palbociclib (0.01 to 12 μM) for 6 days. **f-h)** MCF7 parental and letrozole or anastrazole resistant cells were treated with varying concentrations of palbociclib (Palbo) for 6 days with recovery for 4 days and subjected to f) cell counting to assess cell proliferation; g) flow cytometry to quantify monodansylcadavarine (MDC) staining, a marker of autophagic vesicles; and h) cell counting in combination with 15μM hydroxychloroquine (HCQ) for 6 days. All data represent mean±SD from three independent experiments; p-values were calculated in comparison to cells treated with DMSO (Control) unless indicated. ns:p>0.05; *p<0.05; **p<0.01; ***p<0.001; ****p<0.0001.

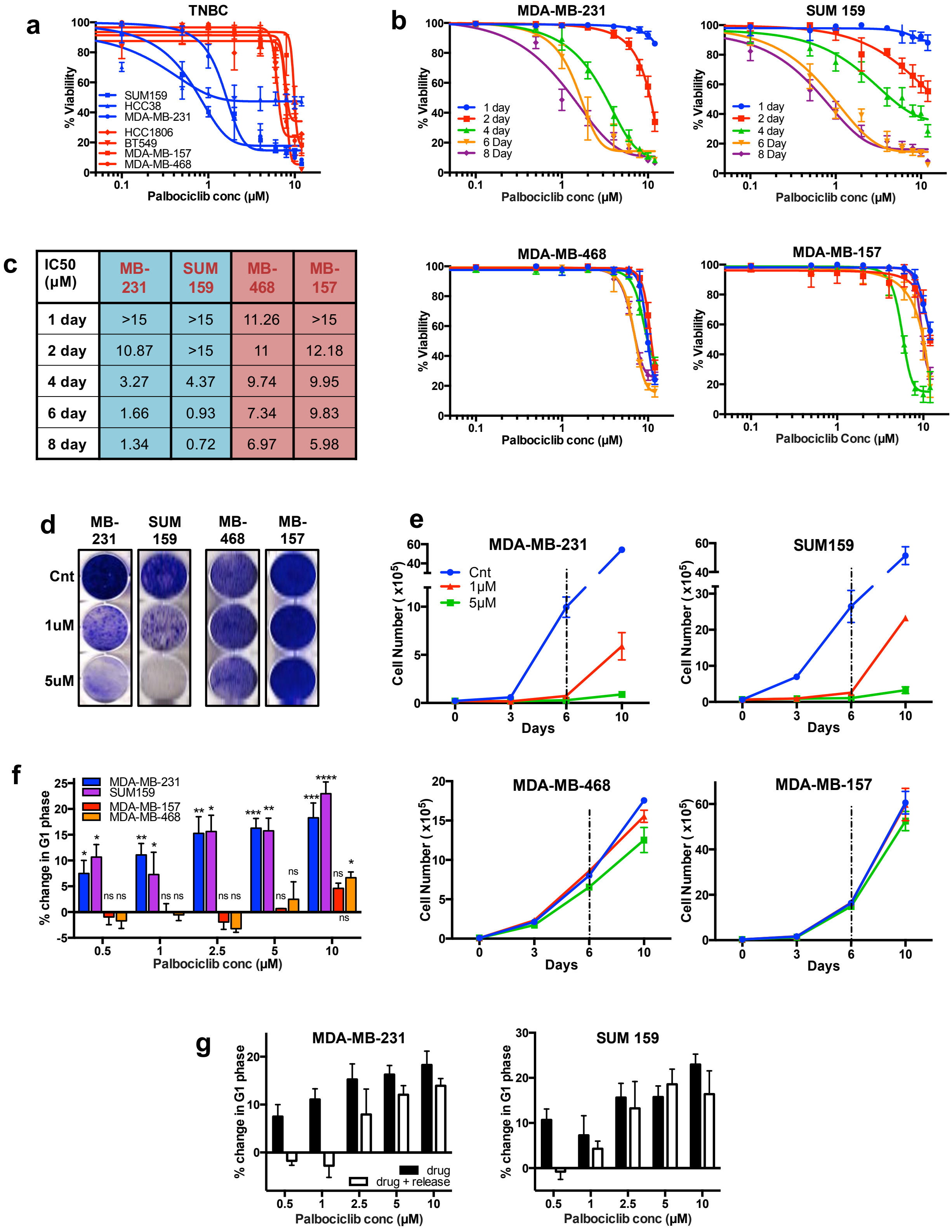

IC50 (µM)	MB-231	SUM 159	MB-468	MB-157
1 day	>15	>15	11.26	>15
2 day	10.87	>15	11	12.18
4 day	3.27	4.37	9.74	9.95
6 day	1.66	0.93	7.34	9.83
8 day	1.34	0.72	6.97	5.98

h

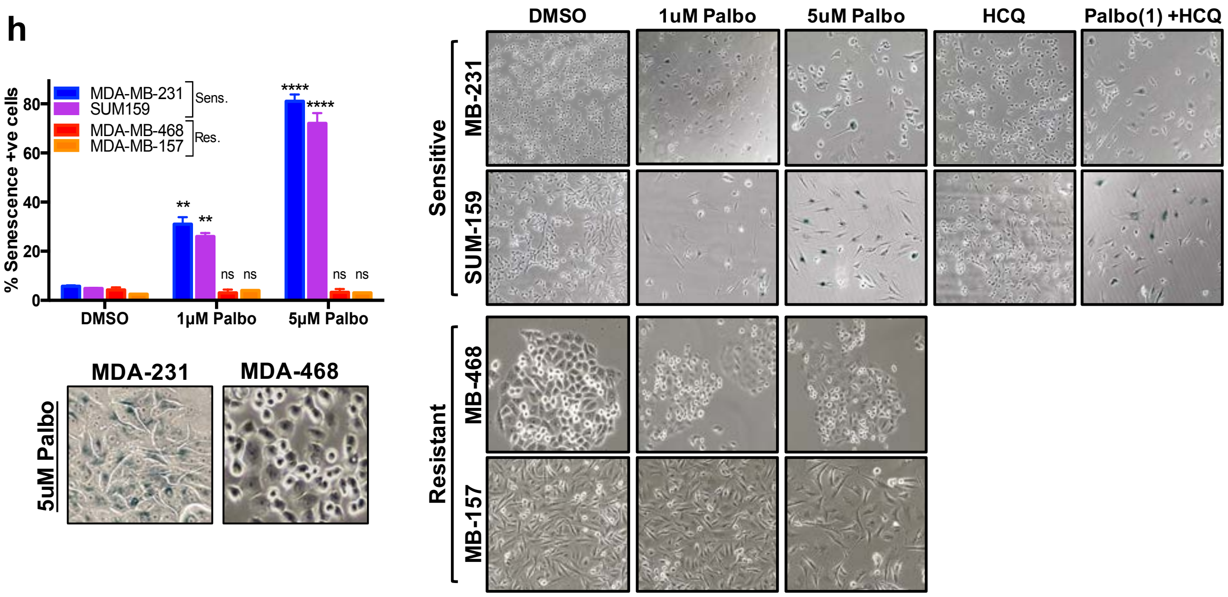

Supplementary Figure 16. Rb+ve LMWE-ve triple-negative breast cancer cell lines are sensitive to palbociclib treatment: a-c) Impact on the growth of the indicated triple-negative breast cancer (TNBC) cell lines of treatment with DMSO or increasing concentrations (conc) of palbociclib (0.01 to 12 μM) for 6 days. Crystal violet staining was performed on day 12 to assess viability and determine the half-maximal inhibitory concentration (IC_{50}) values (c). **d)** Clonogenic assay in TNBC cell lines MDA-MB-231, SUM-159, MDA-MB-468, and MDA-MB-157 cells treated with DMSO (Cnt) or palbociclib (1μM, 5μM) for 6 days and allowed to recover for 6 days. **e-h)** MDA-MB-231, SUM-159, MDA-MB-468, and MDA-MB-157 cells were treated with DMSO or palbociclib (Palbo; 1μM, 5μM) and/or 15 μM autophagy inhibitor hydroxychloroquine (HCQ) for 6 days. Cells were allowed to recover for 4 days to examine reversibility. The cells were subjected to e) cell counting to assess proliferation; f) cell cycle analysis to assess change in G1 phase; g) cell cycle analysis to determine the effect of recovery (release) on change in the G1 phase; h) Senescence activity by SA-ß gal staining and representative images. All data represent mean±SD from three independent experiments; p-values were calculated in comparison to DMSO (Control) unless indicated. ns: $p>0.05$; *$p<0.05$; **$p<0.01$; ***$p<0.001$; ****$p<0.0001$.

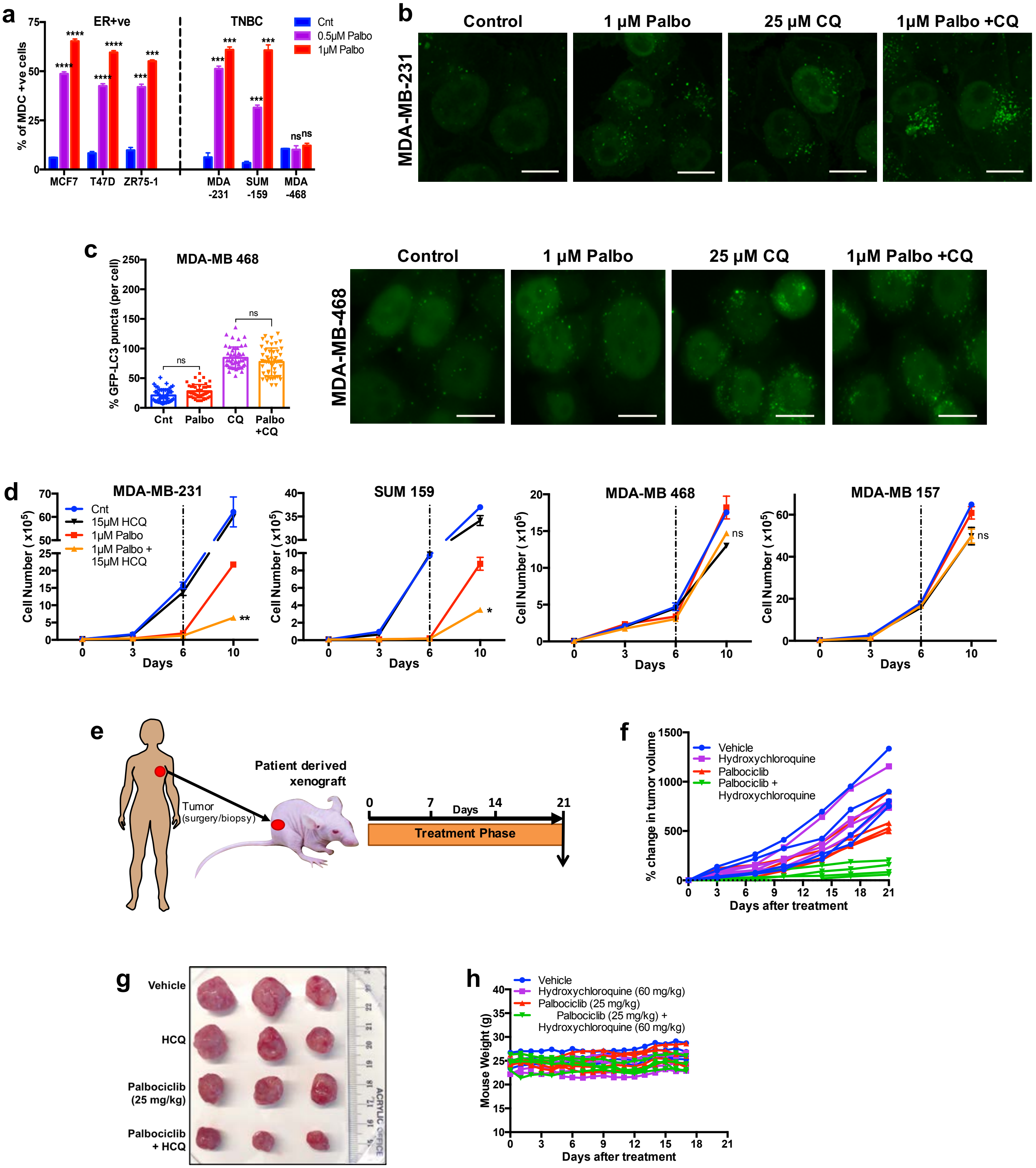
a
ER+ve
TNBC
Cnt
0.5μM Palbo
1μM Palbo
% of MDC +ve cells
MCF7
T47D
ZR75-1
MDA -231
SUM -159
MDA -468
b
Control
1 μM Palbo
25 μM CQ
1μM Palbo +CQ
MDA-MB-231
c
MDA-MB 468
% GFP-LC3 puncta (per cell)
Cnt
Palbo
CQ
Palbo +CQ
MDA-MB-468
d
MDA-MB-231
SUM 159
MDA-MB 468
MDA-MB 157
Cnt
15μM HCQ
1μM Palbo
1μM Palbo + 15μM HCQ
Cell Number (x10^5)
Days
e
Tumor (surgery/biopsy)
Patient derived xenograft
Days
Treatment Phase
f
Vehicle
Hydroxychloroquine
Palbociclib
Palbociclib + Hydroxychloroquine
% change in tumor volume
Days after treatment
g
Vehicle
HCQ
Palbociclib (25 mg/kg)
Palbociclib + HCQ
h
Vehicle
Hydroxychloroquine (60 mg/kg)
Palbociclib (25 mg/kg)
Palbociclib (25 mg/kg) + Hydroxychloroquine (60 mg/kg)
Mouse Weight (g)
Days after treatment

Supplementary Figure 17. Synergism between autophagy inhibition and palbociclib in TNBC cell lines and PDX tumors with intact G1/S checkpoint: a) Measurement of monodansyl-cadavarine (MDC) positive acidic vesicles, including autophagosomes, by flow cytometry in ER positive (MCF7, T47D, ZR75-1) and TNBC (MDA-MB-231, SUM-159, MDA-MB-468) cell lines treated with varying concentrations of palbociclib for 6 days. **b)** Representative confocal images of GFP-LC3 expressing MDA-MB-231 cells treated with 25 μM CQ (for 1 hour), 1 μM palbociclib or combination of palbociclib and CQ for 48 hours. Scale bars are 50 μm. **c)** Representative confocal images and quantification of GFP-LC3 puncta in MDA-MB-468 cells treated with 25 μM CQ (for 1 hour), 1 μM palbociclib or combination of palbociclib and CQ for 48 hours. Scale bars are 50 μm. **d)** Cell counting was used to assess proliferation of TNBC cell lines after combined treatment with DMSO or 1 μM palbociclib and/or HCQ (15 μM) for 6 days and allowed to recover for 4 days to examine reversibility (p-values calculated in comparison with 1 μM palbociclib). **e)** Schematic showing establishment and treatment schedule of patient-derived xenograft (PDX) tumors with vehicle (0.5% methylcellulose and PBS), 60 mg/kg HCQ, 25 mg/kg palbociclib, or palbociclib+ HCQ for 21 days. Images of the mouse and the human body were taken with permission from ScienceSlides by VisiScience Corp. **f)** Percentage change in volume (normalized to volume on Day 0) of the individual PDX tumors after treatment as described in (e) for 21 days (n=4 mice/treatment group). **g)** Representative PDX tumor images from each treatment group harvested at end of 21 days treatment as described in (e). **h)** Individual mouse weights after 21 days of treatment as described in (e). All in vitro data represent mean±SD from three independent experiments; p-values were calculated in comparison to DMSO (Control). ns: $p>0.05$; *$p<0.05$; **$p<0.01$; ***$p<0.001$; ****$p<0.0001$.

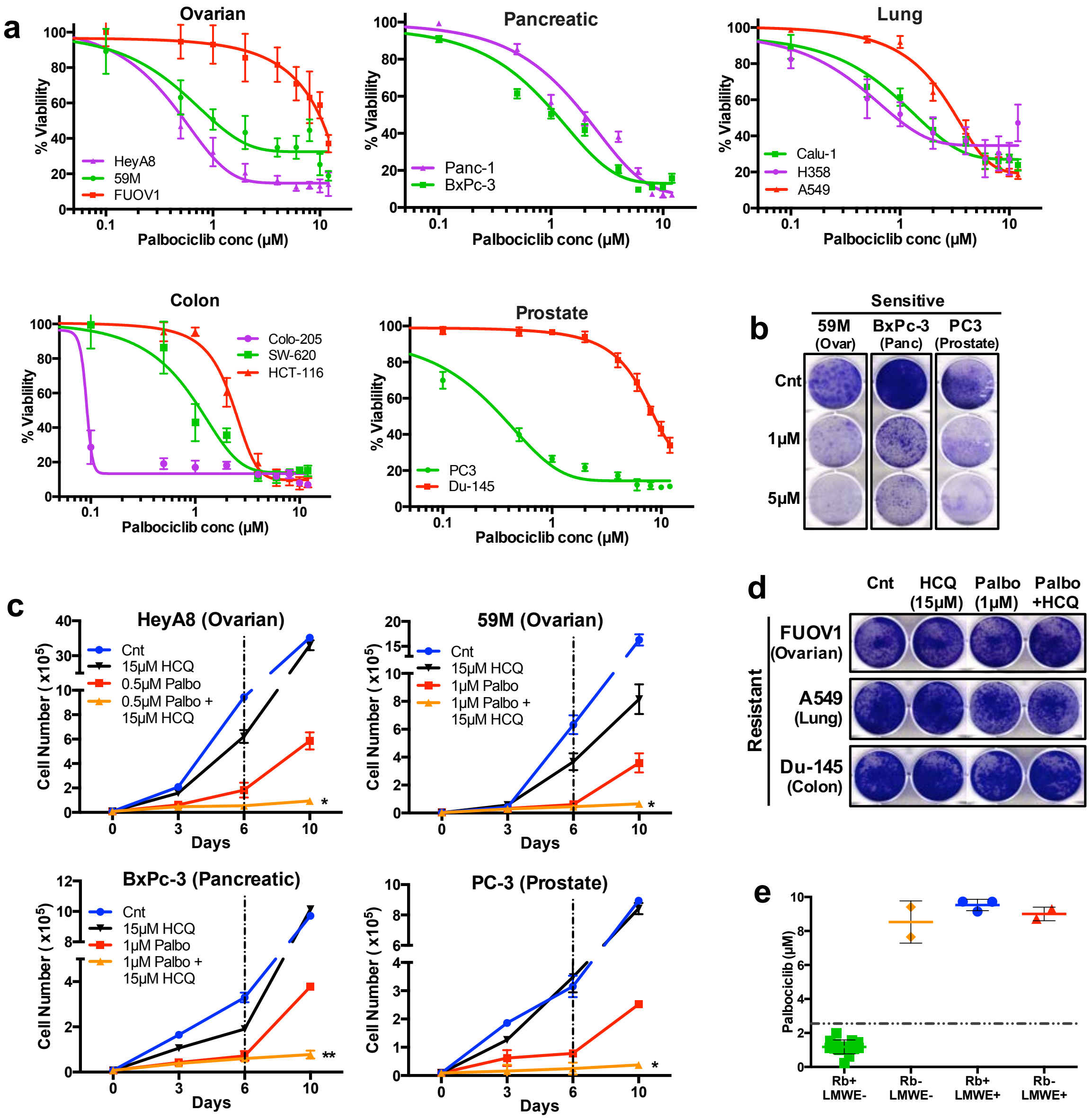

Supplementary Figure 18. Synergism between autophagy inhibition and palbociclib in solid tumor cell lines with an intact G1/S checkpoint: a) Impact on the growth of the indicated ovarian, lung, pancreatic, colon, and prostate cancer cell lines of treatment with increasing concentrations (conc) of palbociclib (0.01 to 12 µM) for 1, 2, 4, 6, or 8 days. **b)** Clonogenic assay in the indicated cell lines treated with DMSO (Cnt) or palbociclib (1 µM, 5 µM) for 6 days and allowed to recover for 6 days. **c)** Cell counting was used to assess proliferation of Hey-A8, 59M, BxPc-3, and PC-3 cells treated with 1 µM palbociclib and/or 15 µM hydroxychloroquine (HCQ) for 6 days and allowed to recover for 4 days to examine reversibility. p-values were calculated in comparison to cells treated with 1 µM palbociclib. **d)** Clonogenic assay in FUOV1, A549, and Du-145 cells treated with DMSO or 1 µM palbociclib (Palbo) and/or HCQ (15 µM) for 6 days and allowed to recover for 6 days. **e)** Correlation between palbociclib half-maximal inhibitory concentration (IC_{50}) values (obtained from dose-response studies in all cancer cell lines) and levels of Rb and low-molecular-weight cyclin E isoform (LMW-E) proteins. All data represent mean±SD from three independent experiments; ns:p>0.05; *p<0.05; **p<0.01; ***p<0.001; ****p<0.0001.

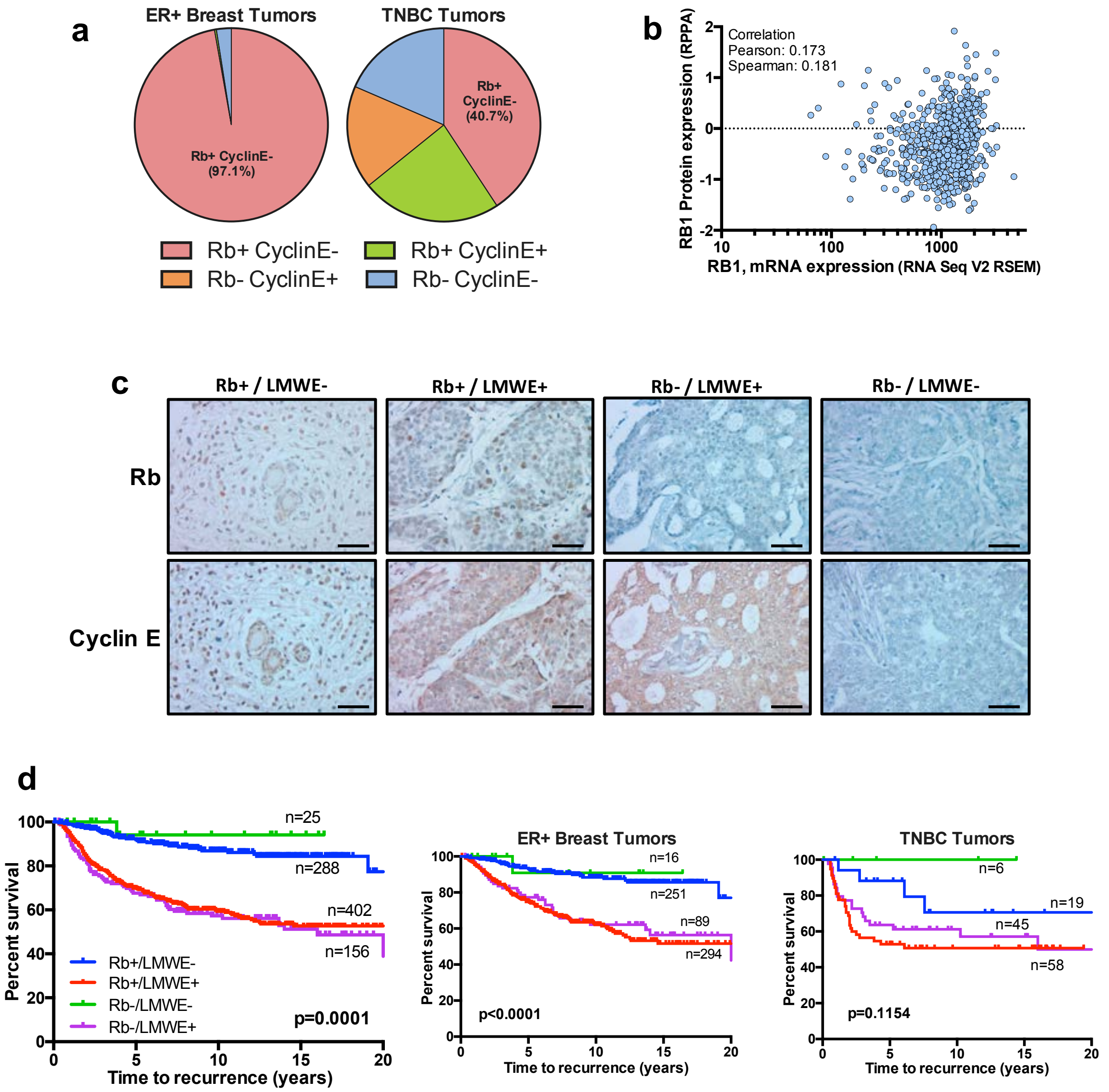

Supplementary Figure 19. Expression of Rb and low-molecular-weight isoforms of cyclin E in breast cancer patients: a) Alterations in Rb and cyclin E RNA levels in ER+/Luminal A and B (n=321) and TNBC/Basal (n=81) tumors from the TCGA database of breast cancer patients. **b)** Plot showing correlation between mRNA and protein levels of Rb among 817 tumors from the TCGA database of breast cancer patients. **c)** Representative images from immunohistochemical analysis of Rb and LMW-E in tumors from the NCI patient cohort (n=879). Scale bars equal 50 μm. **d)** Kaplan Meier curves showing survival of breast cancer patients from the NCI patient cohort when classified based on Rb and LMW-E.

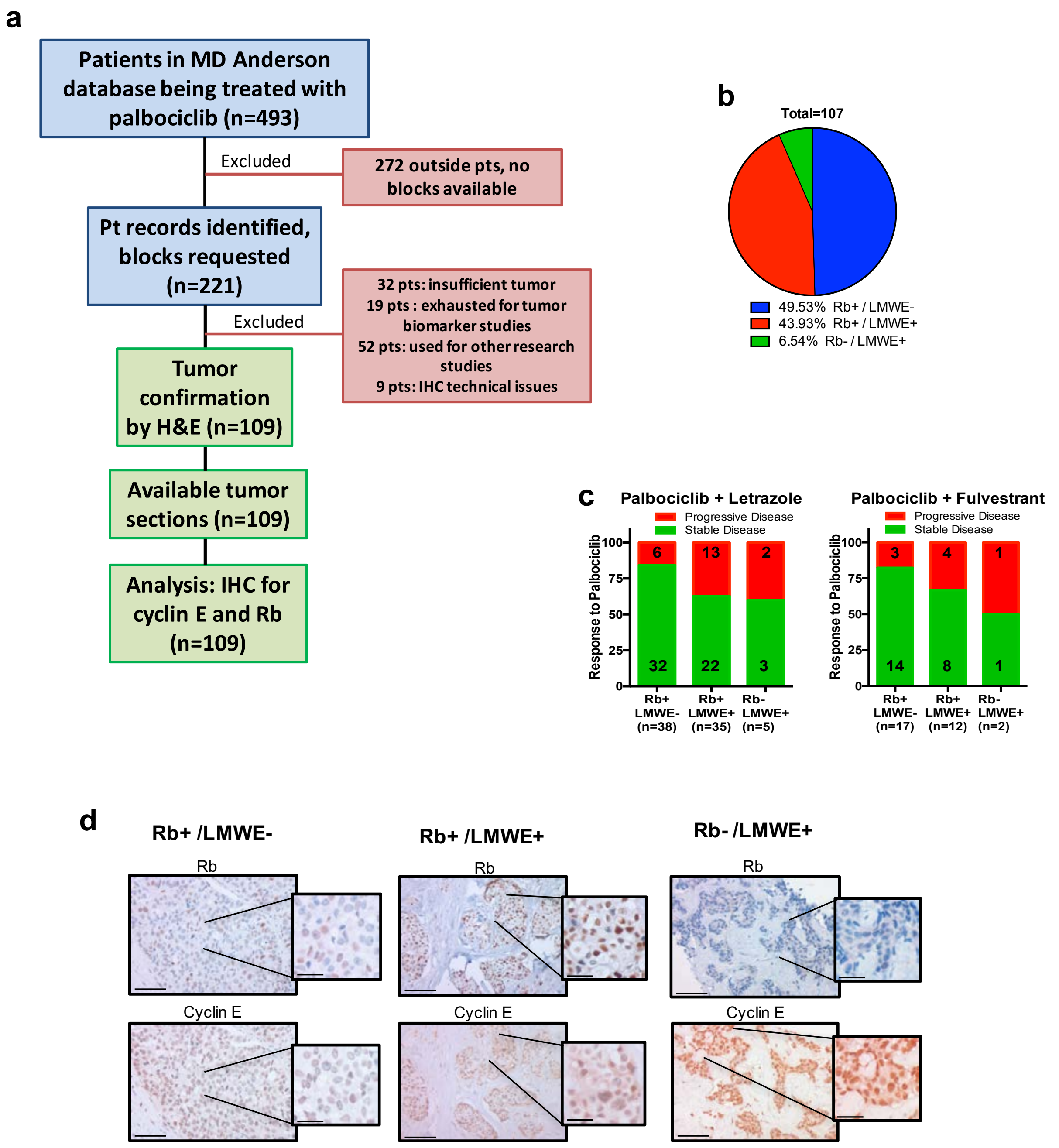

Supplementary Figure 20. Rb and low-molecular-weight isoforms of cyclin E are prognostic biomarker in ER+ve breast cancer patients: a) Schematic showing the total number of palbociclib treated patient tissues obtained and stained for Rb and cyclin-E. **b)** Proportions of 54 breast cancer patients treated with palbociclib with Rb+/LMWE-, Rb+/LMWE+ or Rb-/LMWE+ status. **c)** Proportion of 109 breast patients classified based on treatment with letrozole or fulvestrant with Rb+/LMW-, Rb+/LMWE+ or Rb-/LMWE+ status and their disease progression (response to palbociclib). **d)** Representative images from immunohistochemical analysis of Rb and LMW-E in tumors from patients with ER+ breast cancer treated with palbociclib, classified on the basis of response and Rb/LMW-E status. Scale bars equal 50 μm and insert scale bars equal 20 μm.

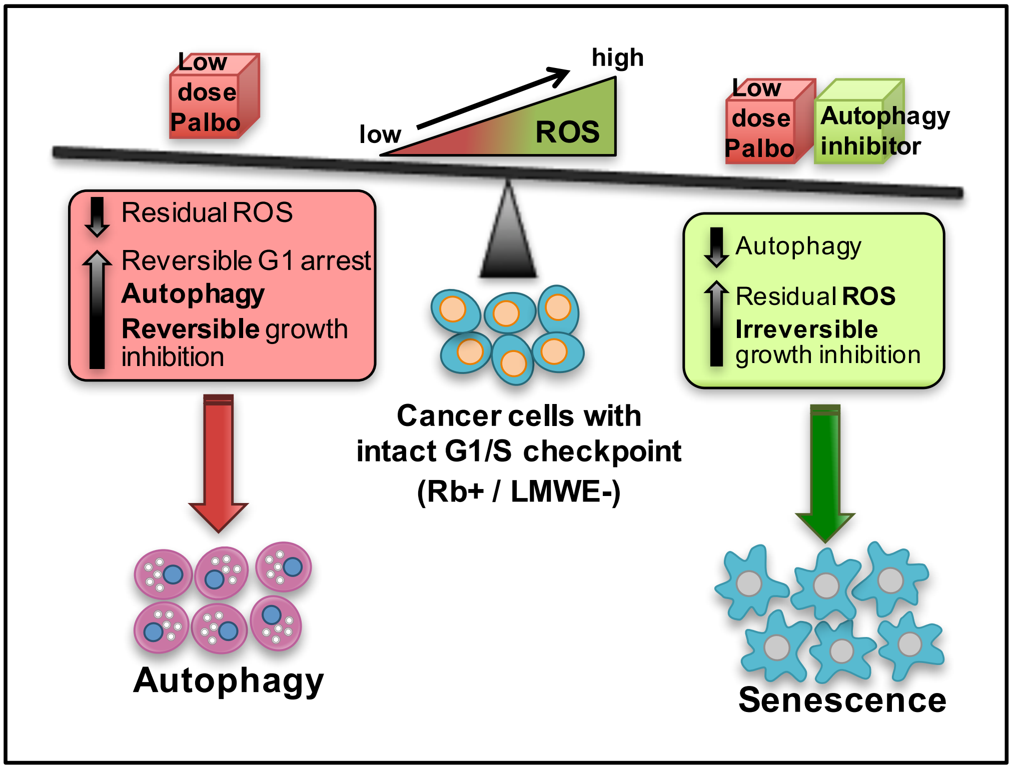

Supplementary Figure 21. Graphical abstract: Schematic depicting the role of CDK4/6 inhibition (i.e., palbociclib [Palbo]) and combined CDK4/6 and autophagy inhibition in regulating autophagy, reactive oxygen species (ROS), and senescence in cancer cells with intact G1/S transition.

Supplementary Figure 22: Uncropped blots for main figures

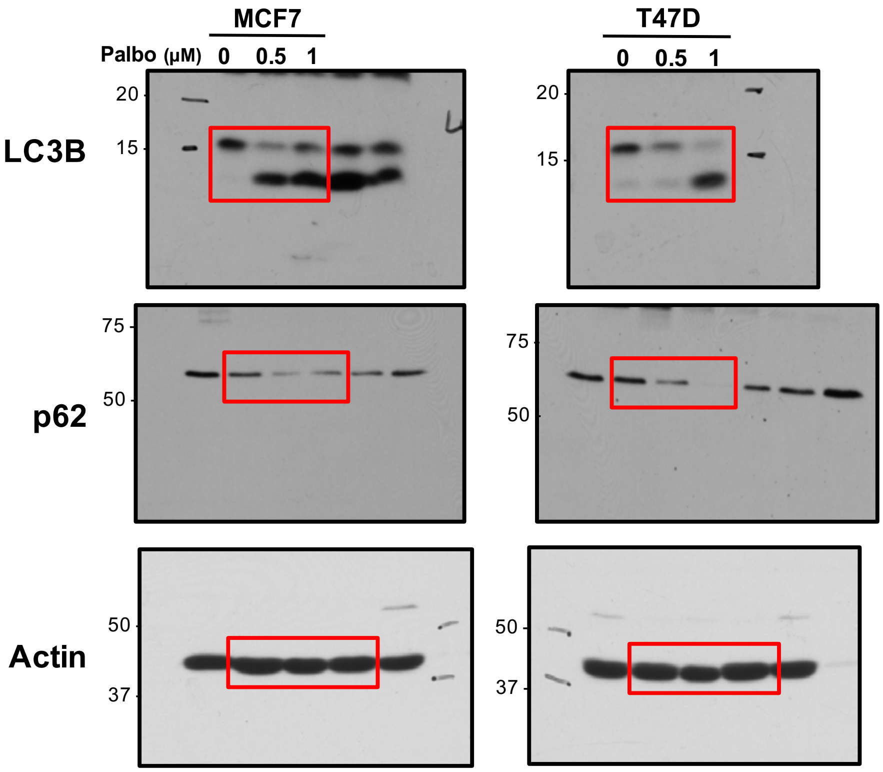

Uncropped blots corresponding to Fig. 1g

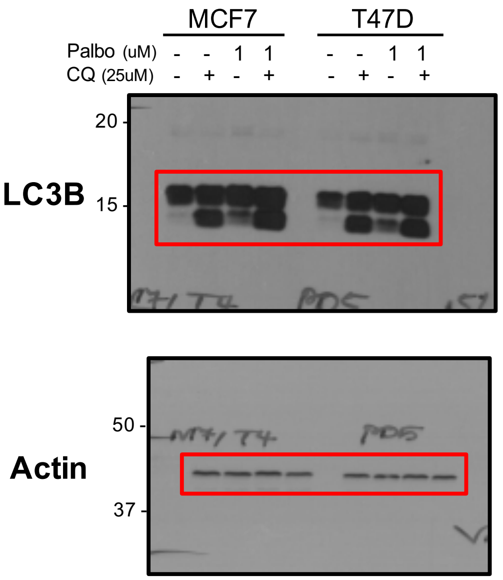

Uncropped blots corresponding to Fig. 1i

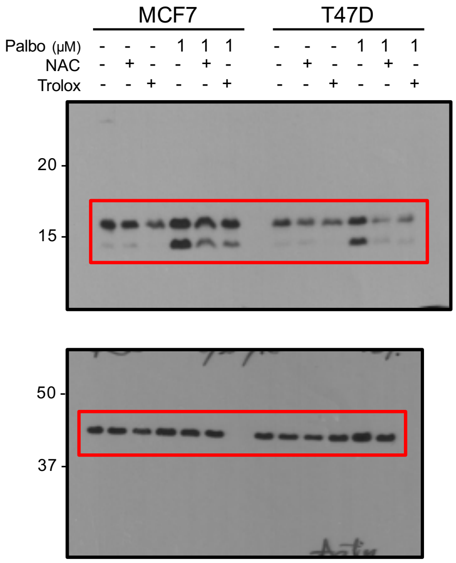

Uncropped blots corresponding to Fig. 1n

Supplementary Figure 22 (contd.): Uncropped blots for main figures

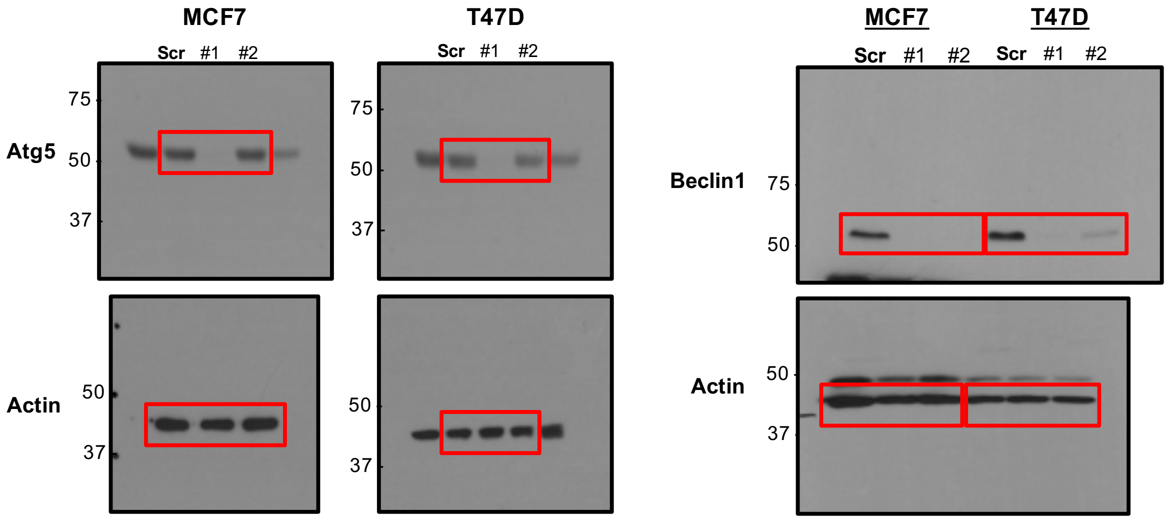

Uncropped blots corresponding to Fig. 2a

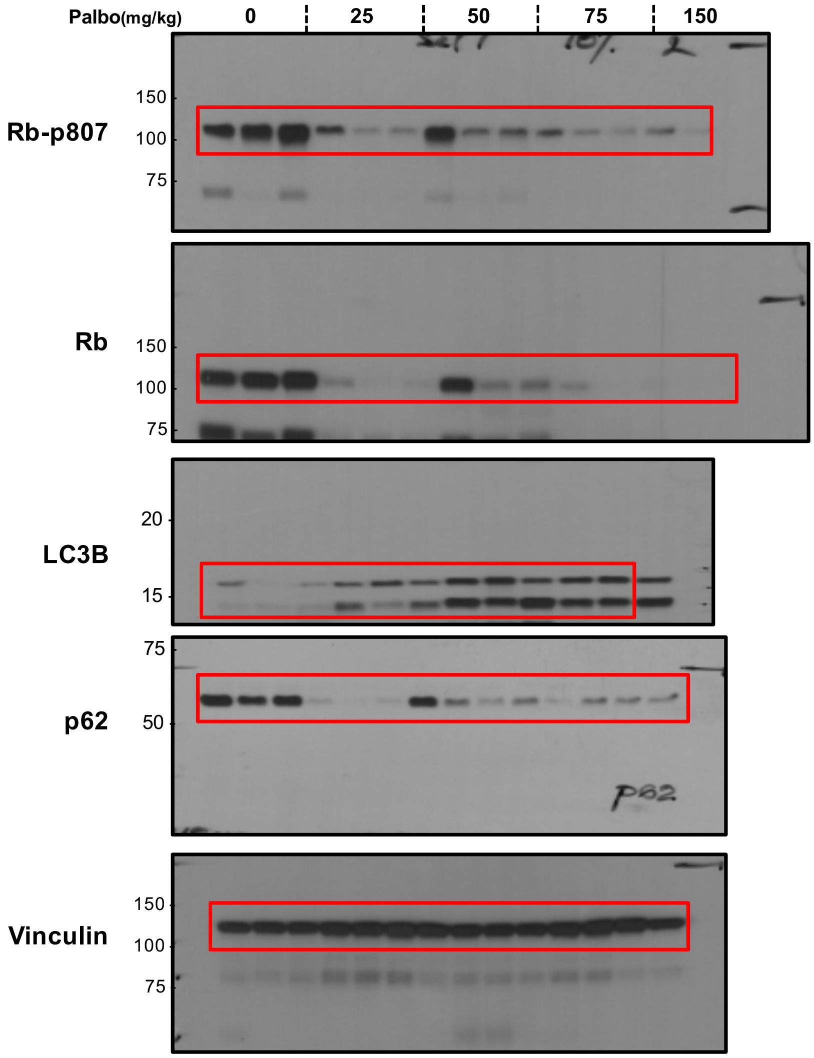

Uncropped blots corresponding to Fig. 3c

Supplementary Figure 22 (contd.): Uncropped blots for main figures

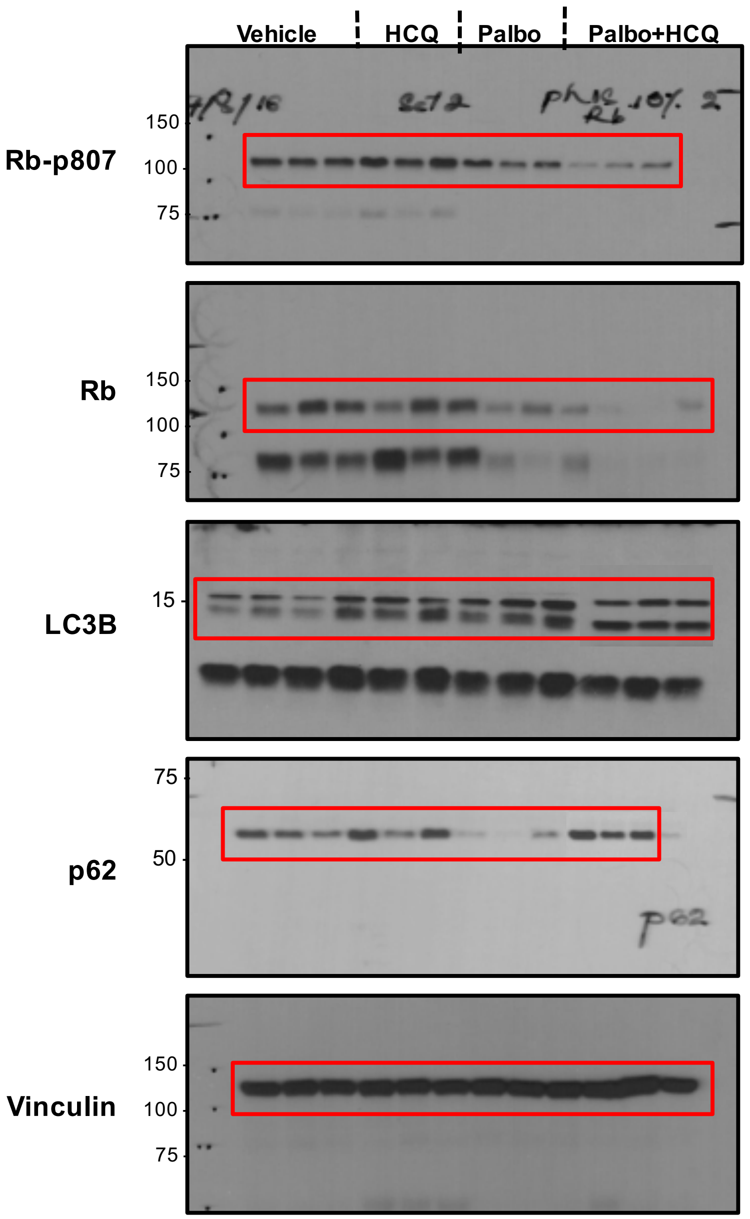

Uncropped blots corresponding to Fig. 3i

Supplementary Figure 22 (contd.): Uncropped blots for main figures

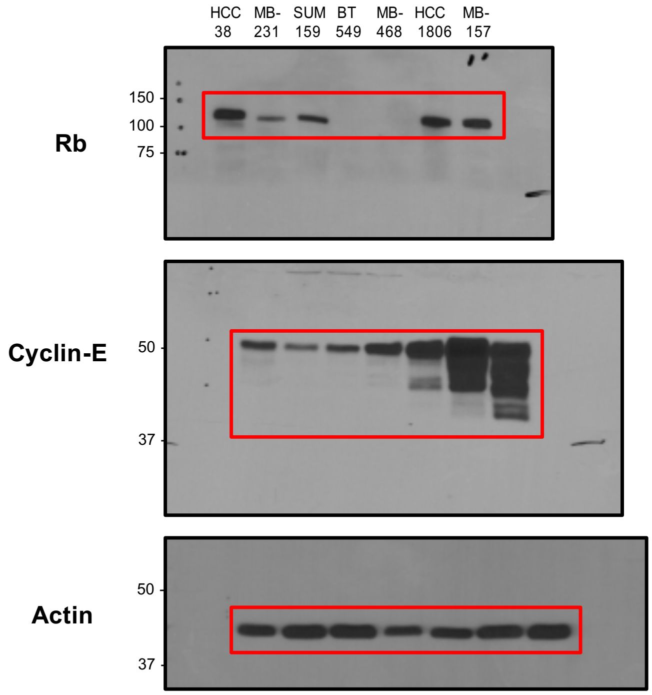

Uncropped blots corresponding to Fig. 5a

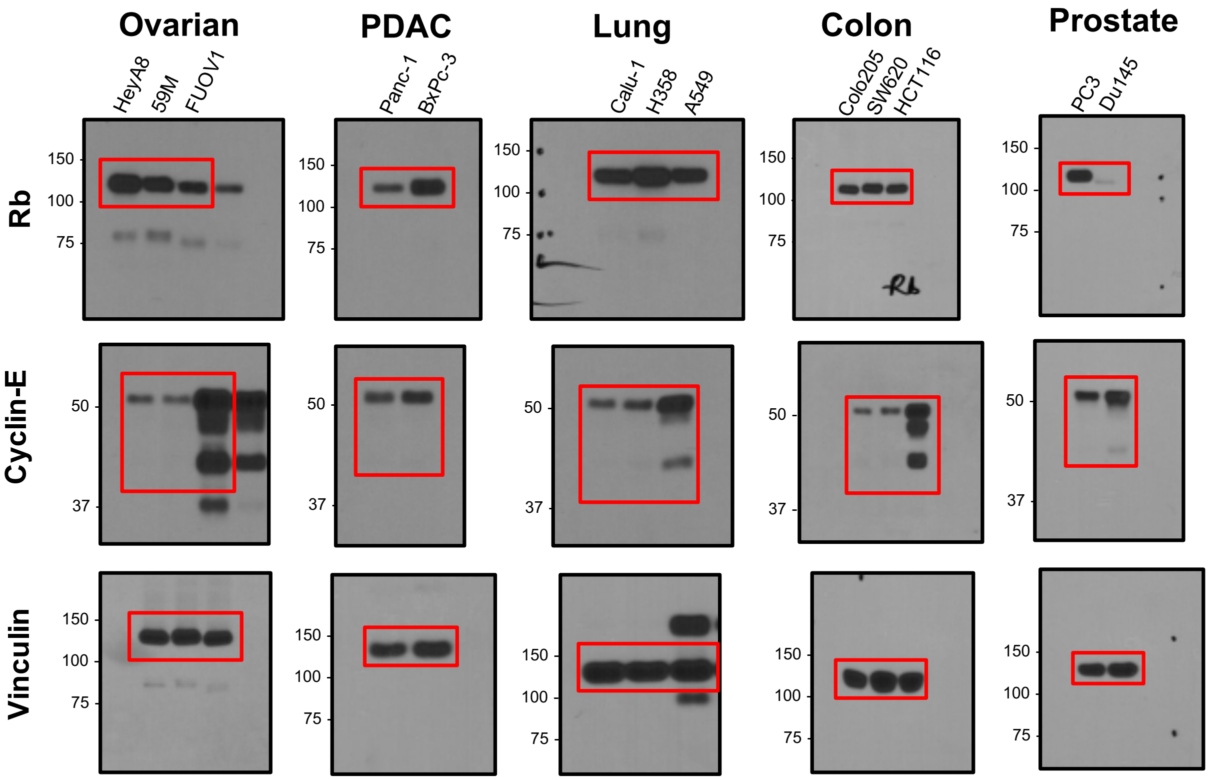

Uncropped blots corresponding to Fig. 5i

Supplementary Figure 23: Uncropped blots for supplementary figures

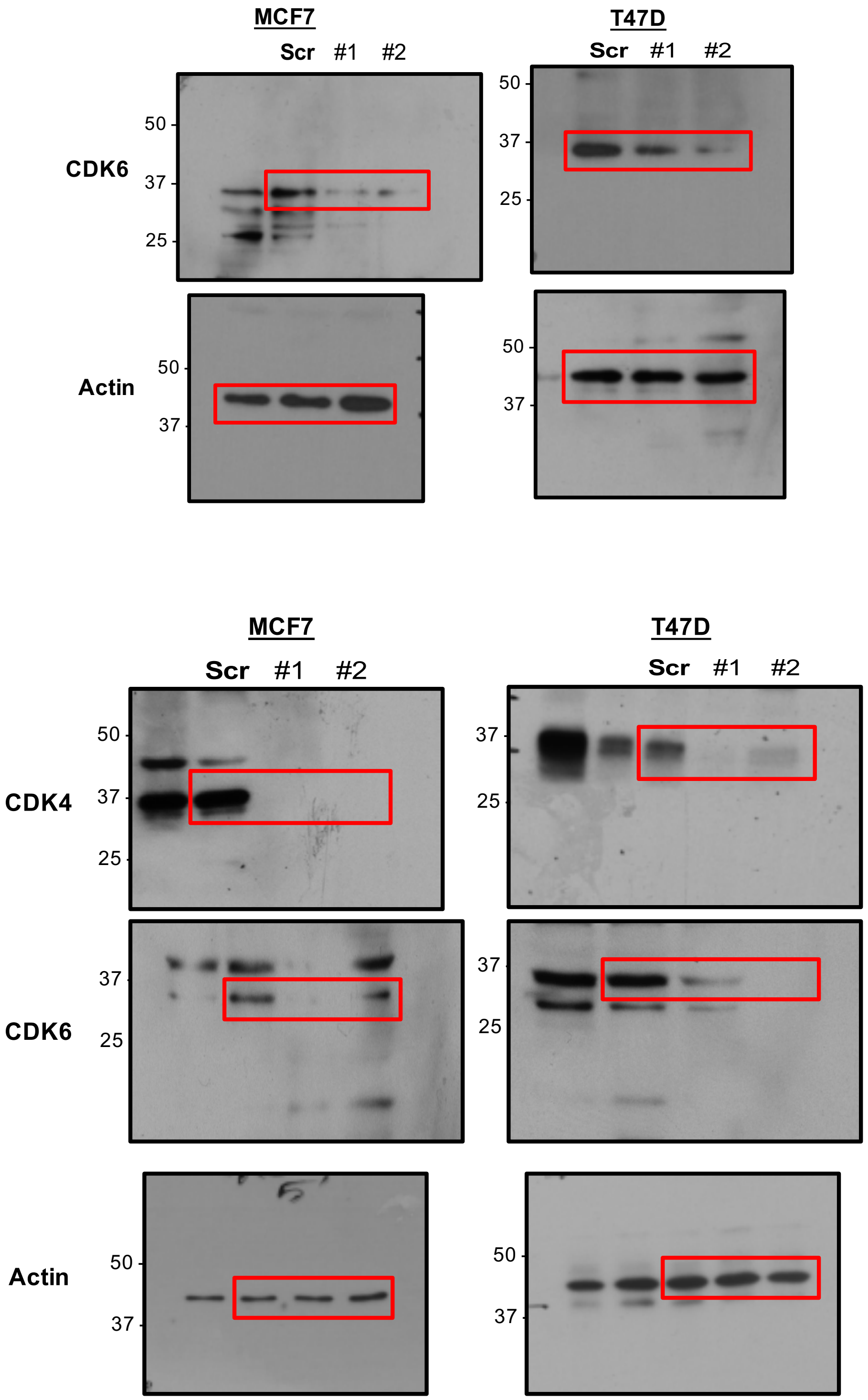

Uncropped blots corresponding to Supp. Fig. 1c

Supplementary Figure 23 (contd.): Uncropped blots for supplementary figures

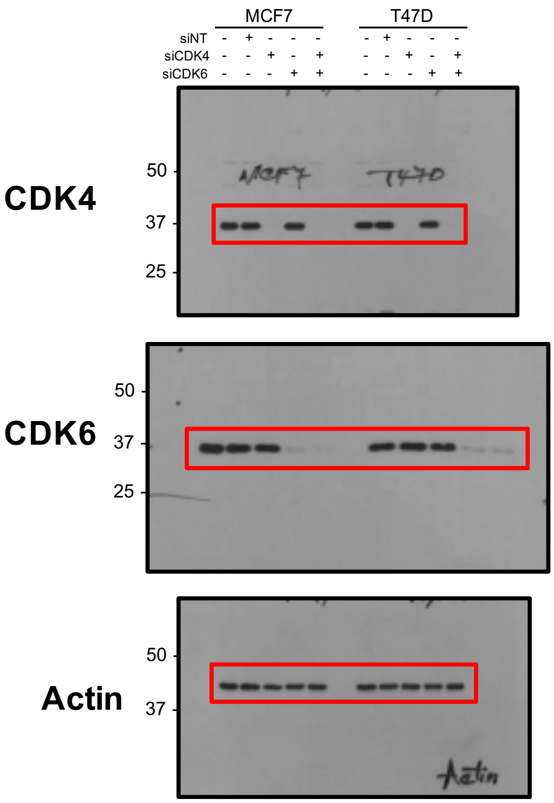

Uncropped blots corresponding to Supp. Fig. 1f

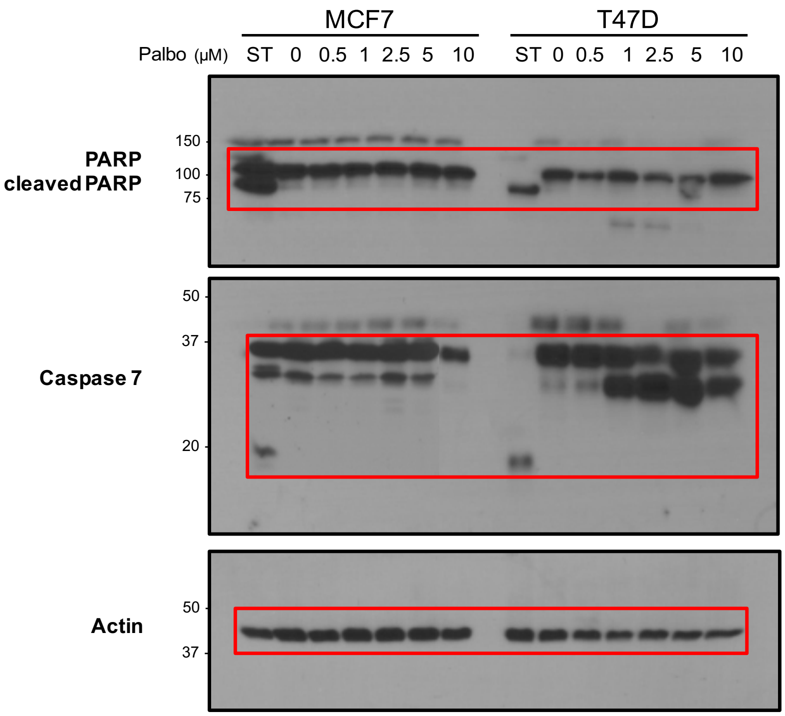

Uncropped blots corresponding to Supp. Fig. 3d

Supplementary Figure 23 (contd.): Uncropped blots for supplementary figures

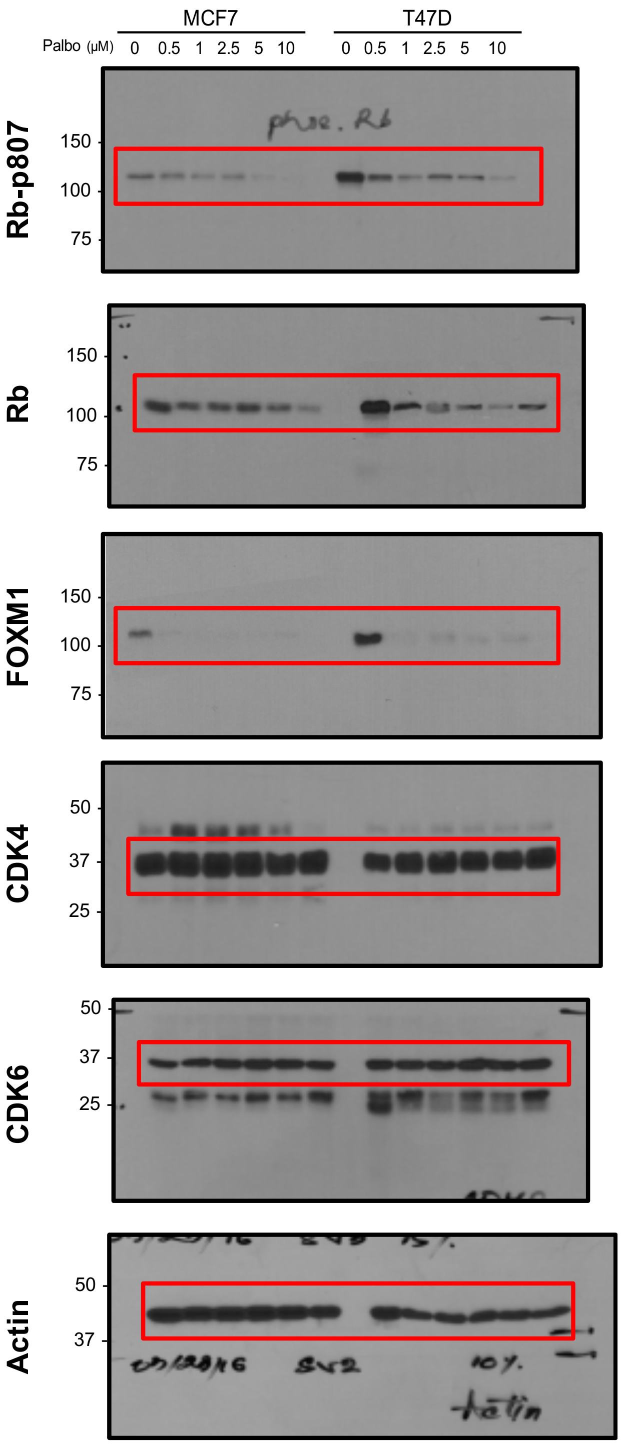

Uncropped blots corresponding to Supp. Fig. 3g

Supplementary Figure 23 (contd.): Uncropped blots for supplementary figures

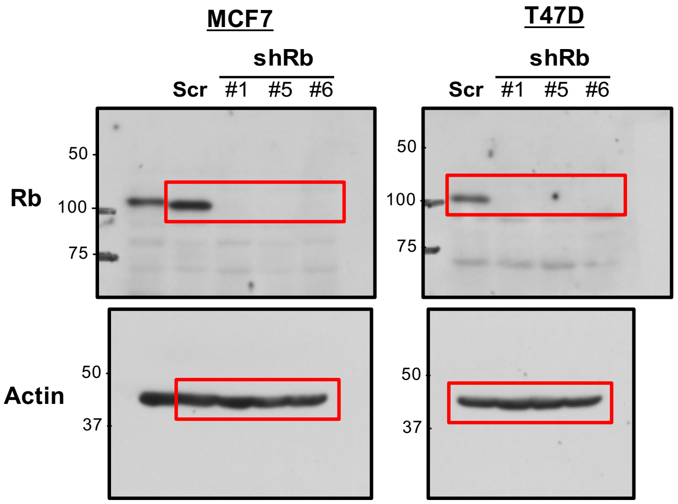

Uncropped blots corresponding to Supp. Fig. 13d

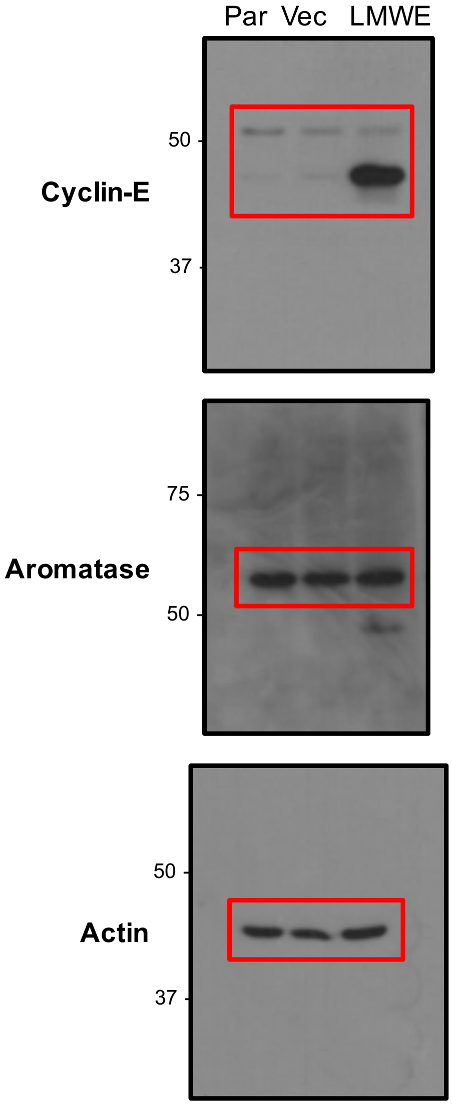

Uncropped blots corresponding to Supp. Fig. 15b

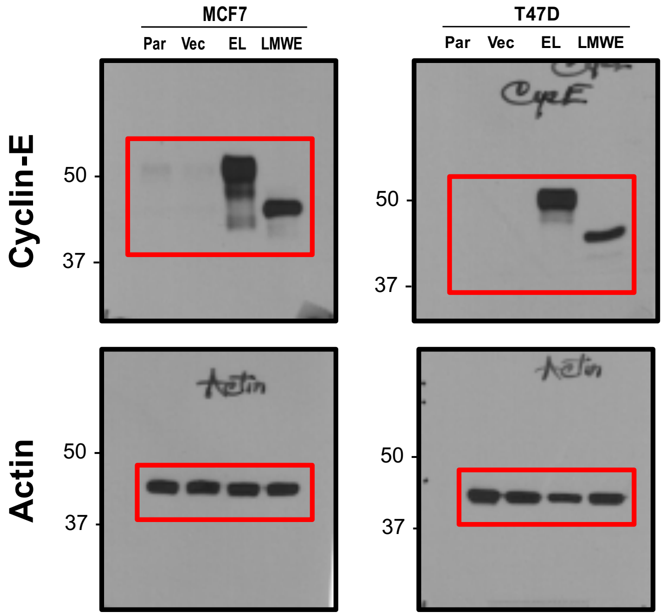

Uncropped blots corresponding to Supp. Fig. 14a

Supplementary Table 1. Clinical, pathologic, and treatment characteristics of patients treated with palbociclib + letrozole

Variable	N	6-month progression rate (%)	12-month progression rate (%)	P value Univariate)
All patients	78	22.2	31.0	
Race				0.3
White	55	25.1	33.2	
Others	23	14.6	24.1	
Clinical tumor stage at diagnosis				0.004
0/I	12	28.7	52.5	
II	20	26.3	36.8	
III	19	38.1	54.2	
IV	25	0	0	
Unknown	1	0	0	
Tumor Histology				0.9
Ductal	67	21.6	31.2	
Others	11	27.1	27.1	
Lymphatic/vascular invasion				0.2
Yes	52	15.2	24.5	
No	24	37.5	45.3	
Adjuvant Chemotherapy				0.004
No	64	16.1	21.5	
Yes	14	48.7	69.2	
Tumor grade				0.4
I/II	57	25.3	38.9	
III	21	15.3	15.3	
Progesterone receptor status				0.02
Positive	69	16.2	26.2	
Negative	9	70.8	70.8	
Rb status				**0.02**
Negative	5	66.7	0	
Positive	73	20.3	29.2	
LMWE status				**0.01**
Negative	38	14.4	18.0	
Positive	40	30.9	44.2	
Rb/LMWE status				**0.006**
Rb+/LMWE-	38	14.4	18.0	
Rb+/LMWE+	35	27.4	41.4	
Rb-/LMWE+	5	66.7	0	
Bone /soft tissue vs. any visceral				0.03
Bone / soft tissue	51	13.7	20.7	
Any visceral	27	37.1	47.6	
Prior therapy for metastatic disease				0.1
None	56	16.6	25.8	
Hormonal	4	25.0	25.0	
Chemotherapy and/or hormonal	18	40.9	50.7	
Time from completion of adjuvant hormonal therapy (years)				0.2
None (de novo metastatic)	42	15.8	23.5	
< 1 year	5	0	0	
> 1 year	31	33.0	42.9	

*p value calculated after excluding unknown.

Supplementary Table 2. Clinical, pathologic, and treatment characteristics of patients treated with palbociclib + fulvestrant

Variable	N	6-month progression rate (%)	12-month progression rate (%)	P value Univariate
All patients	31	17.4	58.0	
Race				0.9
White	25	15.9	63.8	
Others	6	20.0	20.0	
Clinical tumor stage at diagnosis				0.2
0/I	7	0	33.3	
II	7	20.0	40.0	
III	11	14.3	31.4	
IV	6	46.7	-	
Tumor Histology				0.7
Ductal	25	15.3	54.4	
Others	6	25.0	-	
Lymphatic/vascular invasion				0.8
Yes	15	16.4	61.0	
No	16	17.5	45.0	
Adjuvant Chemotherapy				**0.06**
No	16	25.0	80.3	
Yes	15	10.0	25.0	
Tumor grade				0.6
I/II	27	19.3	55.3	
III	4	0	-	
Progesterone receptor status				0.6
Positive	28	18.4	0	
Negative	3	58.6	-	
**Rb status**				**0.03**
Negative	2	-	-	
Positive	29	13.7	56.1	
**LMWE status**				**0.009**
Negative	17	0	52.1	
Positive	14	58.4	79.2	
**Rb/LMWE status**				**0.009**
Rb+/LMWE-	17	0	52.2	
Rb+/LMWE+	12	52.6	76.3	
Rb-/LMWE+	2	-	-	
Bone /soft tissue vs. any visceral				0.2
Bone / soft tissue	20	13.2	62.8	
Any visceral	1	23.8	68.3	
Prior therapy for metastatic disease				0.9
None	9	16.7	-	
Hormonal	14	16.4	68.7	
Chemotherapy and/or hormonal	8	20.0	46.7	
Time from completion of adjuvant hormonal therapy (years)				0.5
None	14	18.0	-	
< 1 year	7	25.0	-	
> 1 year	10	12.5	41.7	

*p value calculated after excluding unknown.

Supplementary Table 3. Cox model for univariate analyses for the factors associated with PFS

Factor	Palbociclib + letrozole (n=78)			Palbociclib + fulvestrant (n=31)		
	**HR**	**P value**	**95% CI**	**HR**	**P value**	**95% CI**
Age at diagnosis, years	1.0	0.06	0.9-1.0	1.0	1.0	0.9-1.1
Height (cm)	0.6	0.3	0.2-1.7	1.2	0.02	1.02-1.3
Weight	1.0	0.8	0.9-1.1	1.0	0.6	0.96-1.02
Race						
White	Referent			Referent		
Others	0.99	0.3	0.96-1.01	0.8	0.9	0.1-6.8
Clinical tumor stage at diagnosis						
0/I	Referent			Referent		
II	0.7	0.5	0.2-2.2	2.0	0.6	0.2-21.9
III	1.3	0.7	0.4-3.9	1.7	0.7	0.2-18.7
IV	0.1	0.02	0.01-0.6	6.1	0.1	0.7-55.0
Tumor Histology						
Ductal	Referent			Referent		
Others	0.9	0.9	0.2-3.8	1.4	0.7	0.3-7.1
Lymphatic/vascular invasion						
Yes	Referent			Referent		
No	1.8	0.2	0.7-4.2	0.9	0.8	0.2-3.3
Adjuvant Chemotherapy						
No	Referent			Referent		
Yes	3.4	0.007	1.4-8.2	0.3	0.09	0.1-1.2
Tumor grade						
I/II	Referent			Referent		
III	0.6	0.4	0.2-1.8	1.7	0.6	0.2-14.6
Progesterone receptor status						
Positive	Referent			Referent		
Negative	3.2	0.02	1.2-8.9		-	
**Rb status**						
Negative	Referent			Referent		
Positive	0.2	**0.04**	0.04-0.9	0.09	**0.09**	0.01-1.4
**LMWE status**						
Negative	Referent			Referent		
Positive	3.2	**0.02**	1.2-8.2	5.2	**0.02**	1.3-20.3
**Rb/LMWE status**						
Rb+/LMWE-	Referent			Referent		
Rb+/LMWE+	2.9	**0.03**	1.01-7.6	4.7	**0.03**	1.1-19.3
Rb-/LMWE+	9.2	**0.008**	1.8-47.4	23.8	**0.04**	1.3-450.1
Bone /soft tissue vs. any visceral						
Bone / soft tissue	Referent			Referent		
Any visceral	2.6	0.03	1.1-6.2	2.2	0.2	0.6-8.3
Prior therapy for metastatic disease						
None	Referent			Referent		
Hormonal	0.9	0.9	0.1-6.8	0.8	0.8	0.1-4.6
Chemotherapy and/or hormonal	2.6	0.04	1.03-6.7	0.6	0.7	0.1-5.5
Time from completion of adjuvant hormonal therapy (years)						
None	Referent			Referent		
< 1 year	7.32e-16	1.0	0-	2.1	0.4	0.3-13.0
>= 1 year	1.9	0.2	0.8-4.4	0.7	0.7	0.2-3.4

Supplementary Table 4. Cox model for multivariable analyses for the factors associated with PFS

	Palbociclib + letrozole			Palbociclib + fulvestrant		
**Factor**	HR	P value	95% CI	HR	P value	95% CI
Progesterone receptor status						
Positive	Referent			Referent		
Negative	4.5	0.007	1.5-13.4		NS	
Prior therapy for metastatic disease						
None	Referent			Referent		
Hormonal	2.4	0.4	0.3-21.4		NS	
Chemotherapy and/or hormonal	4.1	0.006	1.5-11.2		NS	
**Rb status**						
Negative	Referent			Referent		
Positive	0.2	**0.06**	0.04-1.03	0.2	**0.3**	0.01-3.3
**LMWE status**						
Negative	Referent			Referent		
Positive	3.2	**0.03**	1.1-8.7	4.7	**0.03**	1.1-19.3
**C-index**			0.76			
CPE			0.73			
AIC			153.3			
